# The miR-23a~27a~24-2 microRNA cluster buffers transcription and signaling pathways during hematopoiesis

**DOI:** 10.1371/journal.pgen.1006887

**Published:** 2017-07-13

**Authors:** Jeffrey L. Kurkewich, Justin Hansen, Nathan Klopfenstein, Helen Zhang, Christian Wood, Austin Boucher, Joseph Hickman, David E. Muench, H. Leighton Grimes, Richard Dahl

**Affiliations:** 1 Department of Biological Sciences, University of Notre Dame, Notre Dame, IN, United States of America; 2 Harper Cancer Research Institute, South Bend, IN, United States of America; 3 Department of Microbiology and Immunology, Indiana University School of Medicine, South Bend, IN, United States of America; 4 Division of Immunobiology, Cincinnati Children's Hospital Medical Center, Cincinnati, OH, United States of America; Stanford University School of Medicine, UNITED STATES

## Abstract

MicroRNA cluster *mirn23a* has previously been shown to promote myeloid development at the expense of lymphoid development in overexpression and knockout mouse models. This polarization is observed early in hematopoietic development, with an increase in common lymphoid progenitors (CLPs) and a decrease in all myeloid progenitor subsets in adult bone marrow. The pool size of multipotential progenitors (MPPs) is unchanged; however, in this report we observe by flow cytometry that polarized subsets of MPPs are changed in the absence of *mirn23a*. Additionally, in vitro culture of MPPs and sorted MPP transplants showed that these cells have decreased myeloid and increased lymphoid potential in vitro and in vivo. We investigated the mechanism by which *mirn23a* regulates hematopoietic differentiation and observed that *mirn23a* promotes myeloid development of hematopoietic progenitors through regulation of hematopoietic transcription factors and signaling pathways. Early transcription factors that direct the commitment of MPPs to CLPs (Ikzf1, Runx1, Satb1, Bach1 and Bach2) are increased in the absence of *mirn23a* miRNAs as well as factors that commit the CLP to the B cell lineage (FoxO1, Ebf1, and Pax5). Mirn23a appears to buffer transcription factor levels so that they do not stochastically reach a threshold level to direct differentiation. Intriguingly, *mirn23a* also inversely regulates the PI3 kinase (PI3K)/Akt and BMP/Smad signaling pathways. Pharmacological inhibitor studies, coupled with dominant active/dominant negative biochemical experiments, show that both signaling pathways are critical to *mirn23a*’s regulation of hematopoietic differentiation. Lastly, consistent with *mirn23a* being a physiological inhibitor of B cell development, we observed that the essential B cell transcription factor EBF1 represses expression of *mirn23*a. In summary, our data demonstrates that *mirn23a* regulates a complex array of transcription and signaling pathways to modulate adult hematopoiesis.

## Author summary

MicroRNAs (miRNAs) are small ~22 nucleotide long RNA molecules that are involved in regulating multiple cellular processes through inhibiting the expression of target proteins. We previously identified a gene (*mirn23a*) that codes for 3 miRNAs that control the development of immune cells in the bone marrow. The miRNAs promote the development of innate immune cells, macrophages and granulocytes, while repressing the development of B cells. Here we show that *mirn23a* miRNAs negatively affect the expression of multiple proteins that are involved in directing blood progenitor cells to become B cells. Additionally, we observed that modulation of FoxO1 and Smad proteins, downstream effectors of two signaling pathways (PI3 kinase/ Akt and BMP/ Smad), is critical to direct immune cell development. This is the first observation that these pathways are potentially coregulated during the commitment of blood progenitors to mature cells of the immune system. Consistent with *mirn23a* being a critical gene for committing progenitors to innate immune cells at the expense of B cells, we observed that a critical B cell protein represses the expression of *mirn23a*. In conclusion, we demonstrate the *mirn23a* regulation of blood development is due to a complex regulation of both transcription factors and signaling pathways.

## Introduction

Non-coding RNAs, including microRNAs (miRNAs), play a critical role in regulating hematopoietic gene expression networks. MiRNAs are ~22 nucleotide long RNA molecules that negatively regulate gene expression post-transcriptionally by binding to the 3’ untranslated region (UTR) of target mRNAs[1,2]. We previously screened for miRNAs regulated by the hematopoietic transcription factor PU.1 (Sfpi1) that could be involved in immune cell fate acquisition. We identified the *mirn23a* miRNA cluster as a PU.1 activated gene[3]. The *mirn23a* gene is located on murine chromosome 8 and codes for 3 pre-miRNAs: miR-23a, miR-24-2, and miR-27a. Overexpression of *mirn23a* in hematopoietic progenitors biases cell fate decisions towards the myeloid lineage at the expense of the lymphoid lineage both in vitro and in vivo[3]. Recently we reported that when *mirn23a* is deleted from mice, there is an increase in B cell development and a concomitant decrease in myelopoiesis in the bone marrow that persists in the periphery. This differentiation bias occurs early during hematopoietic development, as an increase in common lymphoid progenitors (CLPs) is observed in *mirn23a*^*-/-*^ bone marrow, as well as a decrease in common myeloid progenitors (CMPs), granulocyte/ monocyte progenitors (GMPs) and megakaryocyte/ erythroid progenitors (MEPs). No differences in hematopoietic stem cells (HSC) and multipotential potential progenitors (MPP) were observed. The overexpression and knockout results suggest that *mirn23a* miRNAs promote myelopoiesis through repressing lymphopoiesis. However, the hematopoietic targets that *mirn23a* regulates to drive myeloid cell development are not clear.

Commitment of bone marrow hematopoietic stem and progenitor cells (HSPCs) to specific lineages is tightly controlled by a complex network of cell intrinsic and extrinsic cues that regulate downstream signaling cascades and transcriptional pathways.[4–7] The initial differentiation event towards a committed hematopoietic lineage is from the MPP into the CLP or CMP. It has recently become appreciated that the MPP pool can be sub-divided into 4 distinct MPP subpopulations that have varying polarizations towards different hematopoietic lineages[8]. Commitment to the CLP from the MPP is heavily dependent on the expression of several hematopoietic transcription factors, including PU.1 (Sfpi1), Ikzf1 (Ikaros) Mef2c, Satb1 and Runx1[9–13]. Commitment to the B cell lineage from the CLP is dependent on B cell fate determinants E2A, EBF1, and FoxO1[14–16]. Pax5 along with Bach1 and Bach2, repress myeloid genes in CLPs and early B cell precursors to lock them into the lymphoid cell fate[17,18].

In this study, we follow up previous overexpression and knockout experiments in an attempt to elucidate hematopoietic pathways regulated by *mirn23a*. Although we previously reported that *mirn23a* loss does not alter the pool of MPPs, here we observe that it does result in changes in the polarized MPP subsets. In vitro culture of MPPs and in vivo transplants reveals that loss of *mirn23a* inhibits myeloid differentiation and promotes lymphoid development from the MPP pool. Gene and protein analysis from multipotential EML cell lines[19] generated from *mirn23a*^*+/+*^ and *mirn23a*^*-/-*^ mice show that misregulation of Ikzf1, Bach1, Satb1, and Runx1 in the absence of *mirn23a* contribute to an increased output of lymphoid cells. Additionally, we observed that *mirn23a* regulated downstream effectors of both the PI3K/Akt and BMP/Smad pathways, suggesting that, *mirn23a* agonizes PI3K/Akt while simultaneously antagonizing BMP/Smad signaling in order to promote myelopoiesis. Our data suggest a model where *mirn23a* regulates hematopoietic differentiation through buffering the expression of transcription factors and coupling the activation and repression of 2 critical signal transduction pathways.

## Results

### Mirn23a regulates MPP3/MPP4 progenitor populations to influence cell fate decisions

**Fig 1:**
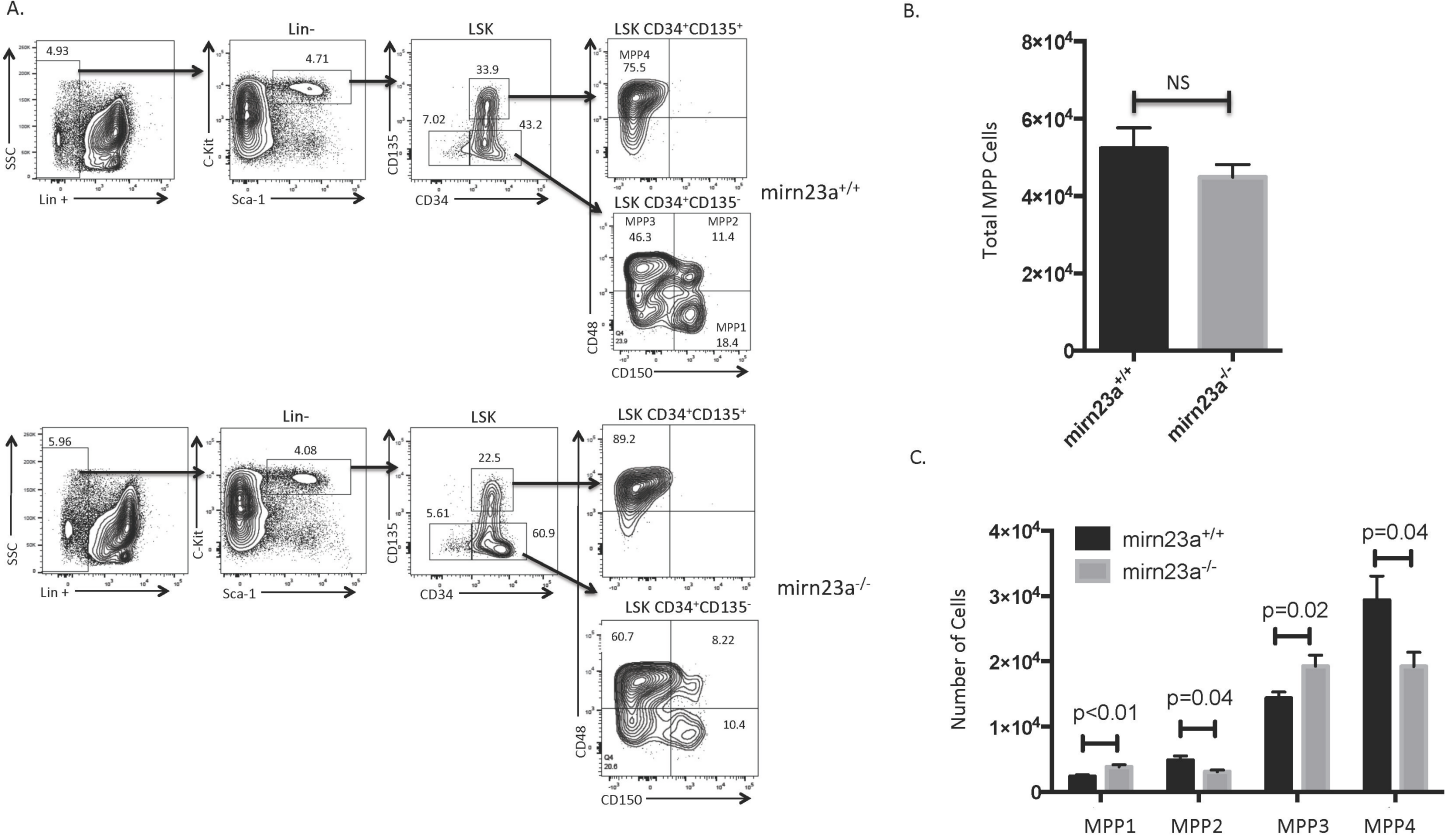
Mirn23a regulates balance of MPP3/MPP4 progenitor populations. Nucleated bone marrow was harvested from the femurs and tibias of wildtype and *mirn23a*^*-/-*^ mice and stained for cell surface markers. **A)** Multipotent progenitor populations MPP1-MPP4 were analyzed by flow cytometry. MPP3 represents a myeloid biased population, while MPP4 represents a lymphoid biased population. Representative plots are shown. **B)** The total MPP population was not significantly changed between wildtype and *mirn23a*^*-/-*^ mice. **C)** *Mirn23a*^*-/-*^ mice had significantly increased MPP1 and MPP3 populations and significantly decreased MPP2 and MPP4 populations. For all MPP analyses, N = 10 wildtype and N = 9 *mirn23a*^*-/-*^ mice were examined. Statistical analysis was performed by unpaired students t-test.

We previously observed that overexpression of *mirn23a* in hematopoietic progenitor’s increased myeloid development at the expense of B cell development[3]. Similarly, analysis of immune cell populations in *mirn23a*^*-/-*^ mice showed increased B cell development at the expense of myeloid development[20]. Analysis of hematopoietic stem and progenitor cell (HSPC) populations revealed increased CLPs and decreased CMPs in *mirn23a*^*-/-*^ mice, while MPP populations were unchanged. Since MPP populations were unchanged in *mirn23a*^*-/-*^ mice, this suggested that *mirn23a* null MPPs are more biased towards the CLP lineage than wildtype MPPs. It has recently become appreciated that the MPP pool consists of at least 4 distinct polarized populations, with the MPP3 being biased toward myeloid differentiation and the MPP4 being biased toward lymphoid differentiation[8]. To examine whether these populations were altered in the bone marrow *mirn23a*^*-/-*^ mice, we stained nucleated hematopoietic cells with the previously described markers to identify MPP1-4 populations (Fig 1A.) This analysis revealed no significant difference in the total MPP pool, consistent with our previous results (Fig 1B). However, when the MPP populations were compartmentalized into MPP1-4 populations, this surprisingly revealed a significant increase in the MPP1 and MPP3 (myeloid-biased) populations, and an accompanying decrease in the MPP2 and MPP4 (lymphoid-biased) bone marrow populations in the *mirn23a*^*-/-*^ mice compared to wildtype (Fig 1C).

**Fig 2:**
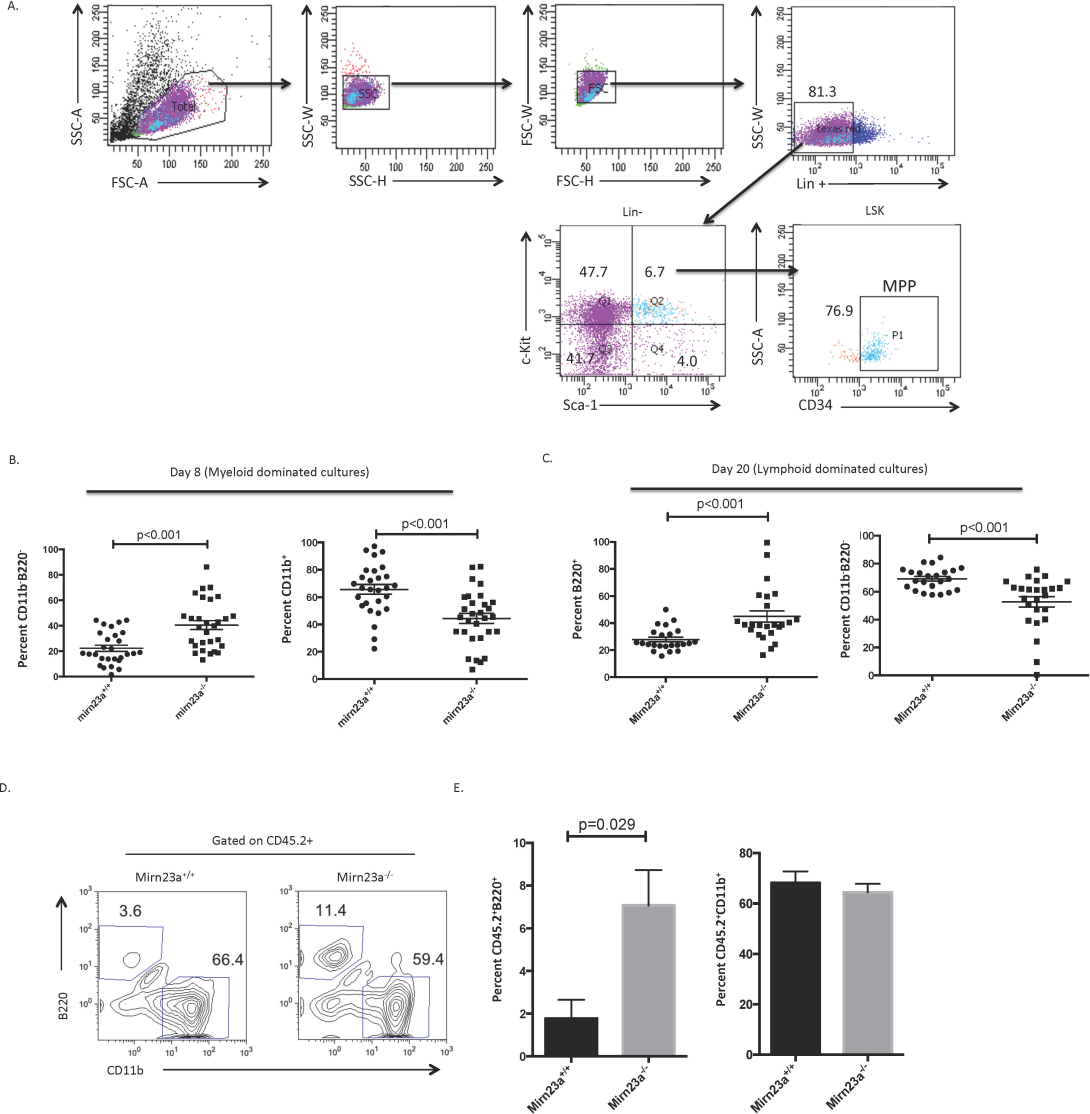
Mirn23a^-/-^ MPPs are functionally polarized to the lymphoid lineage. **A)** Wildtype and mirn23a^-/-^ MPPs were isolated by FACS and used in subsequent functional experiments. **B)** The differentiation potential of wildtype and *mirn23a*^*-/-*^ MPPs to the myeloid or lymphoid lineage was evaluated by sorting 10 MPP cells into a 96 well plate with OP9 stromal cells in the presence of IL-7 and Flt3L cytokines. *Mirn23a*^*-/-*^ MPP wells show a significant increase in their B220^-^CD11b^-^ population compared to wildtype MPPs after 8 days of culture. The increase in B220^-^CD11b^-^ populations in *mirn23a*^*-/-*^ cultures is accompanied by a significant decrease in differentiated CD11b^+^ populations. **C)** *Mirn23a*^*-/-*^ cultures have significantly increased B cell differentiation after 20 days of culture. The increase in B cell differentiation is accompanied by a significant decrease in B220^-^CD11b^-^ populations in *mirn23a*^*-/-*^ cultures. **D)** CD45.2 Wildtype and mirn23a^-/-^ MPPs were transplanted into lethally irradiated CD45.1 congenic recipients with CD45.1 support marrow. Contribution of MPPs to the lymphoid and myeloid lineage was analyzed 4 weeks after transplant. For transplant analysis, N = 4 wildtype and N = 4 *mirn23a*^*-/-*^ mice were examined **E)** Contribution to the lymphoid lineage was significantly increased in mirn23a^-/-^ MPPs. Statistical analysis was performed by unpaired students t-test.

To examine the differentiation potential of *mirn23a*^*-/-*^ MPPs, we performed in vitro differentiations. Previously we observed that culturing a pool of *mirn23a*^*-/-*^ stem and progenitor cells (Lineage negative) on OP9s resulted in increased B lymphopoiesis with decreased myelopoiesis compared to wildtype cells. To determine if MPPs were responsible for this, we sorted 1, 10, or 100 MPP (LSK CD34+) cells onto 96 well plates coated with OP9 stromal cells in the presence of IL-7 and Flt3L for 8 or 20d (Fig 2A). After 20d less than 5% of the wells in which 1 MPP was sorted gave rise to enough cells to analyze, which was not enough to generate any significant conclusions. After 8d, the 10 and 100 cell well cultures contained sufficient cells per well for analysis. Consistent with previous in vitro stromal culture of MPPs, the cultures were predominantly myeloid (CD11b+) at 8d[16]. At 8d, the 10 cell/well *mirn23a*^*+/+*^ cultures consist of ~65–70% CD11b^+^ myeloid cells, while *mirn23a*^*-/-*^ cultures only contain ~40–45% CD11b^+^ cells, suggesting that loss of *mirn23a* hinders or slows myeloid differentiation (Fig 2B). There are no detectable B220+ cells in either culture, but the CD11b^-^B220^-^ double negative population is significantly increased in *mirn23a*^*-/-*^ cells, further suggesting the inhibition of myeloid development from MPP (Fig 2B). Similar results were obtained with 100 cell/well cultures (S1A and S1B Fig). At 20d, there were enough cells in the 10 cell/well culture for flow cytometric analysis (100 cells/well were too dense with extensive cell death at this timepoint precluding meaningful analysis). After 20d, lymphoid cells were present in the cultures (B220+). Individual *mirn23a*^*+/+*^ MPP cultures generated ~25% B220^+^ cells, while mirn23a^-/-^ cultures generated ~45% B220^+^ B cells (Fig 2C). These cultures have no detectable CD11b^+^ populations. This suggests that loss of *mirn23a* promotes lymphoid differentiation from the MPP pool.

To investigate whether mirn23a null M5PPs have increased lymphoid potential in vivo, we sorted LSK CD34+ MPPs (same gating scheme in Fig 2A) from CD45.2 *mirn23a*^*-/-*^ mice and transplanted 2,000 MPPs with 2x10^5^ CD45.1 support marrow cells into lethally irradiated CD45.1 recipient mice. Contribution to the B cell and myeloid cell lineage was examined 4 weeks after transplant and revealed a significant increase in B220^+^ B cells from *mirn23a*^*-/-*^ MPPs (Fig 2D and 2E). Since the contribution to the lymphoid lineage was low compared to the myeloid lineage (~3% vs ~70% respectively in WT mice), decreases in the myeloid lineage were harder to observe, and although we observed a slight decrease in CD11b+ myeloid cells, these changes were not statistically significant (Fig 2E). To investigate whether defects in proliferation could contribute to these phenotypes, we conducted in vivo BrdU experiments that revealed no difference in proliferation between wildtype and mirn23a^-/-^ LSK cells (S2A and S2B Fig). Further analysis by FACS for cell surface markers Annexin V and 7AAD revealed no differences in apoptosis (S2C and S2D Fig). Together, this work shows that *mirn23a*^*-/-*^ MPPs have increased lymphoid potential in vitro and in vivo compared to wildtype MPPs, despite the population having an increase in myeloid-biased cells based on cell surface phenotype.

### Mirn23a loss results in increased lymphoid stem cell gene expression programs

**Fig 3:**
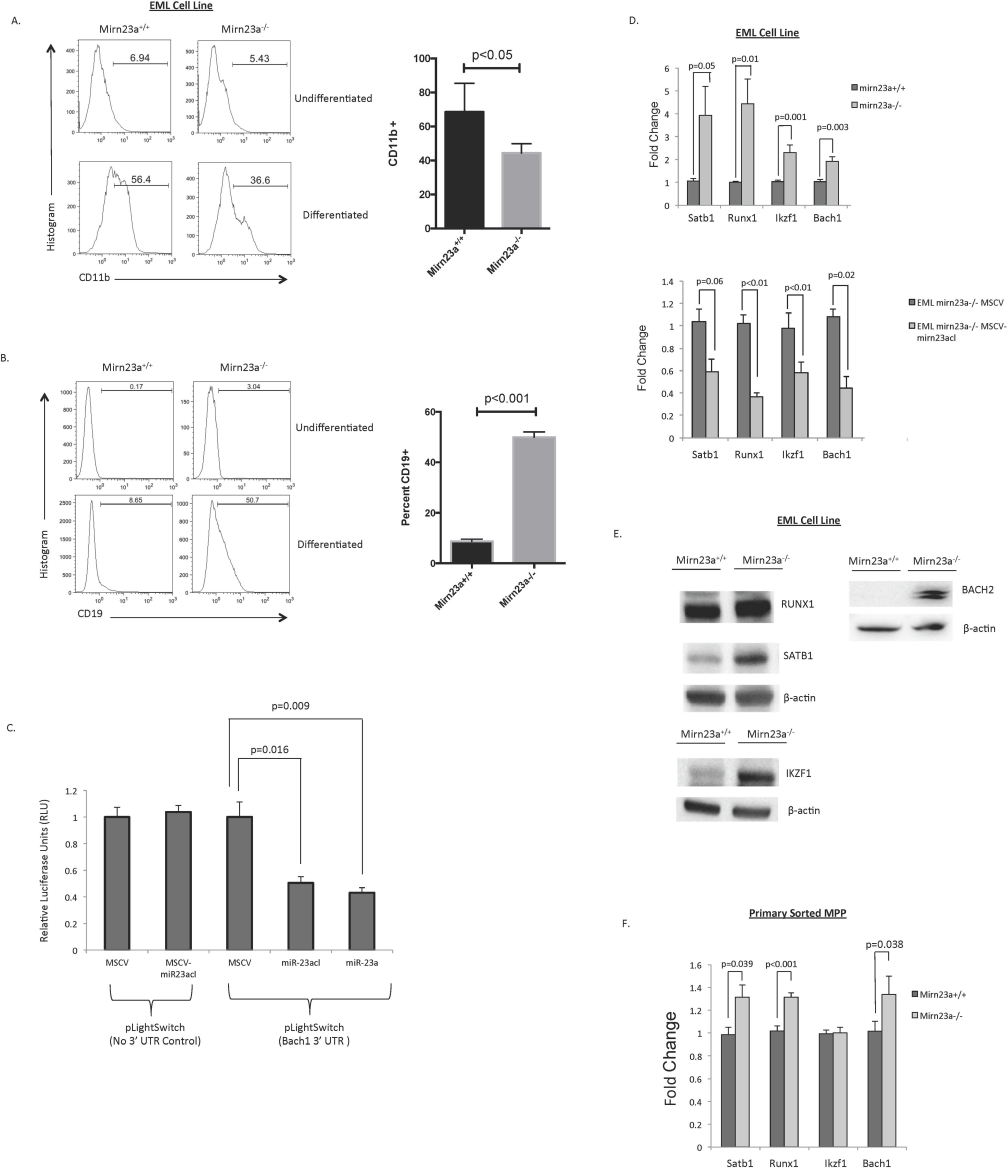
Mirn23a regulates critical hematopoietic gene expression networks to influence cell fate decisions. Primary multipotent EML cells were generated from wildtype and *mirn23a*^*-/-*^ mice. EML differentiation experiments were conducted to evaluate the lymphoid and myeloid potential of these populations. **A)** Mirn23a^-/-^ EMLs were differentiated to the myeloid lineage using ATRA, as well as WEHI-3B and HM5 conditioned media. Mirn23^-/-^ EMLs show decreased myeloid differentiation compared to wildtype EMLs. **B)** Differentiation to the lymphoid lineage was evaluated by OP9 coculture with exogenously supplied IL7 and Flt3L. Mirn23a^-/-^ cells have significantly increased lymphoid potential. **C)** To confirm mirn23a regulation of Bach1, LightSwitch luciferase reporter assays with the Bach1 3’UTR were conducted in 293T cells overexpressing empty vector control (MSCV), the entire mir23a cluster (MSCV-miR-23acl), or miR-23a alone (MSCV-miR-23a). Overexpression of miR-23acl or miR-23a alone results in significantly decreased expression of RLU when the Bach1 3’ UTR is present **D)** RNA was prepared from wildtype and *mirn23a*^*-/-*^ EML cells and analyzed by qRTPCR. Satb1, Runx1, Ikzf1, and Bach1 expression was significantly increased in *mirn23a*^*-/-*^ cells. **E)** Whole cell protein lysates were prepared from wildtype and *mirn23a*^*-/-*^ EML cells and probed for Runx1, Satb1, Ikzf1, and Bach1 expression by immunoblot. Numbers designate fold change in protein levels as determined by densitometry. **F)** RNA was harvested from primary LSK CD34+ MPPs from wildtype and mirn23a^-/-^ mice. Analysis of gene expression by qRT-PCR revealed significantly increased expression of SatB1, Runx1, and Bach1. Statistical analysis was performed by unpaired students t-test.

To elucidate the molecular mechanisms driving increased CLP production from the MPP pool in *mirn23*a null mice, we created multipotent erythroid-myeloid-lymphoid (EML) cell lines from wild type and *mirn23a*^*-/-*^ mouse bone marrow[19]. These cells, along with being multipotent, express stem cell markers c-Kit and CD34, while not expressing any committed lineage markers, including CD11b and B220, suggesting that they model MPPs (S3A Fig). Consistent with our primary bone marrow cell data, differentiation of wildtype and *mirn23a*^*-/-*^ EML cells to the myeloid lineage revealed that *mirn23a*^*-/-*^ EMLs have impaired myeloid differentiation (Fig 3A). Conversely, differentiation of EML cells to the lymphoid lineage was enhanced in *mirn23a*^*-/-*^ EMLs (Fig 3B).

Mechanistically, development of the MPP to the CLP is dependent on the presence of essential hematopoietic transcription factors. Among these critical transcription factors are Runx1, Satb1, Bach1, and Ikzf1, all of which are required for normal lymphoid development[9,11,17,21]. Runx1 and Ikzf1 have been shown to be targets of *mirn23a* miRNAs in hematopoietic cells, and Satb1 was shown to be a direct target in osteosarcoma cells [22–24]. Direct targeting by mirn23a miRNAs for each gene was validated by luciferase-UTR assays. The Bach1 3’UTRs contains a conserved targeting site for miR-23a/b as predicted by the targetscan algorithm[25]. We observe that expression of the entire mirn23a cluster or miR-23a alone represses expression of a luciferase transcript containing the Bach1 3’UTR (Fig 3C).

To determine if *mirn23a* loss affected expression of these factors in multipotent EML cells, we collected RNA from wildtype and *mirn23a*^*-/-*^ EML cells and analyzed for expression of transcription factors by qRT-PCR. This analysis revealed a significant increase in Satb1, Runx1, Bach1, and Ikzf1 mRNA expression in *mirn23a*^*-/-*^ EMLs (Fig 3D). Furthermore, reintroduction of *mirn23a* to mirn23a^-/-^ EMLs decreased expression of these critical lymphoid transcription factors (Fig 3D). To validate the increase in these factors at the protein level, we collected whole cell lysates from wildtype and *mirn23a* null EML cells and analyzed for protein expression by immunoblot. This analysis confirmed that BACH1, RUNX1, SATB1, and IKZF1 protein levels were all increased in *mirn23*^*-/-*^ EML cells (Fig 3E). To examine whether these critical factors were changed in vivo, we collected RNA from sorted LSK CD34+ MPP populations and analyzed gene expression by qRT-PCR. Bach1, Satb1, and Runx1 expression were all significantly increased, while no change was observed in Ikzf1 expression (Fig 3F). Taken together, these results suggest that loss of *mirn23a* results in increased expression of transcription factors that skew the MPP differentiation towards the CLP at higher frequencies than observed in wildtype cells.

### Mirn23a regulates critical B lymphocyte gene expression networks to sustain commitment to the B cell lineage

**Fig 4:**
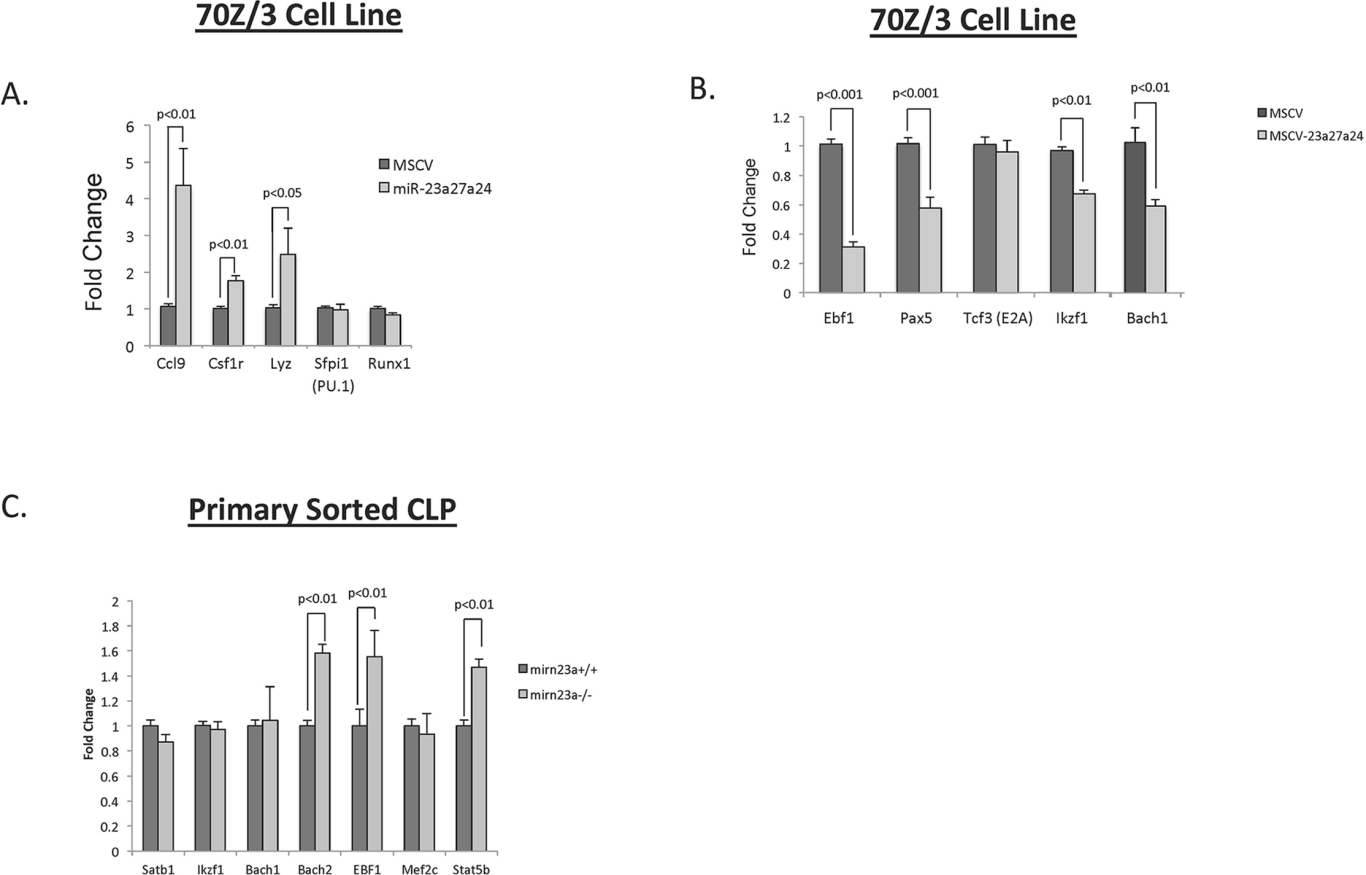
Mirn23a regulates essential B cell promoting genes to sustain commitment to the B cell lineage. To evaluate targets of mirn23a that could promote sustained commitment towards the B cell lineage downstream of the MPP, we overexpressed empty vector control (MSCV) or the mirn23a cluster (MSCV-23a27a24) into 70Z/3 Pre-B cells and evaluated gene expression by qRT-PCR. **A)** Myeloid genes Ccl9, Csf1r, and Lyz were significantly increased when the mirn23a cluster was overexpressed. **B)** Ebf1, Pax5, Ikzf1, and Bach1 gene expression was significantly decreased in 70Z/3 cells overexpressing mirn23a cluster. **C)** RNA was harvested from primary wildtype and mirn23a^-/-^ CLP populations and analyzed for lymphoid gene expression by qRT-PCR. Essential B cell promoting genes EBF1, Stat5b, and Bach2 were significantly increased in mirn23a^-/-^ cells. Statistical analysis was performed by unpaired students t-test.

Previously we observed that the majority of the increased CLP population consists of Ly6D+ B cell biased progenitors (BCPs)[20]. To further identify B cell transcription networks inhibited by *mirn23a* that direct commitment of the CLP to the B cell lineage, we overexpressed the individual cluster miRNAs in the 70Z/3 pre-B lymphoblast cell line using murine stem cell virus (MSCV) retroviruses co-expressing GFP. High expressing lines were generated through limiting dilution and 2 unique lines for each miRNA and a control line infected with empty retrovirus were analyzed for genome wide RNA expression by microarray analysis using Affymetrix Mouse Genome 430 2.0 Arrays (S1–S3 Tables). Examining genes differentially expressed 2 fold or more we observed that exogenous miR-24-2 expression results in upregulation of myeloid cell associated genes, including Lyz, Ccl9, and Csf1r. This is consistent with our previous study showing that miR-24 alone could enhance myeloid development in vitro[3]. To validate the upregulation of myeloid specific genes, RNA was prepared from control or *mirn23a* cluster (miRs-23a, -24-2, and -27a) overexpressing 70Z/3 cells and gene expression was assayed by qRTPCR. MiR-24 expression is ~8 fold overexpressed in these cells (S4A Fig). Myeloid genes Ccl9, Csf1r, and Lyz1 were upregulated in cells overexpressing all 3 cluster miRNAs (Fig 4A). We also examined expression of the transcription factors Sfpi1 (PU.1) and Runx1 that are known to regulate these genes, but did not observe significant changes. However we cannot rule out that activity and/ or protein expression of PU.1 and RUNX1 is affected. Overexpression of the individual cluster miRNAs demonstrated that miR-24 alone could enhance Ccl9 and Csf1r expression (S4B Fig).

The increased myeloid gene expression could potentially be due to decreased expression of essential B cell transcription factors E2A (Tcf3), Ebf1 and Pax5[16,26,27]. We isolated RNA from 70Z/3 MSCV, or MSCV-*mirn23a* cluster expressing cells and assayed for gene expression of E2A (Tcf3), Ebf1, Pax5, and Ikzf1. This analysis revealed significantly decreased Ebf1, Pax5, and Ikzf1 expression in *mirn23a* expressing cells, while no change was observed in E2A expression (Fig 4B). In addition, since the transcription factors Bach1 was shown to repress myeloid genes in lymphoid cells, we analyzed their expression in 70Z/3 cells and observed that it is downregulated by *mirn23a* expression (Fig 4B). In contrast to the myeloid genes examined above, the majority of the lymphoid genes were downregulated by the expression of a single miRNA member of the cluster (S4C Fig). To investigate whether lymphoid transcription factors were increased in primary cells lacking mirn23a, we sorted CLP populations from wildtype and mirn23a^-/-^ bone marrow and analyzed gene expression by qRT-PCR. This analysis revealed significantly increased expression of EBF1 (Fig 4C). Bach1 was not observed to be affected by loss of mirn23a, however the related factor Bach2, which also represses myeloid associated genes, was overexpressed in mutant CLPs[17].

To identify pathways affected by mirn23a that could promote lymphoid development, we performed a gene set enrichment analysis (GSEA)[28] with the expression data obtained from the 70Z/3 microarrays. A heat map of a ranked list of the top 50 upregulated and downregulated genes in the miRNA expressing 70Z/3 lines compared to the empty vector expressing lines is shown (S5 Fig). The miRNA expressing lines were enriched for genes associated with IL2/Stat5 signaling pathway in control cells compared to miRNA overexpressing cell lines (S6 Fig). Examination of gene expression in in *mirn23a*^*-/-*^ CLPs showed that Stat5b is upregulated in the absence of mirn23a miRNAs (Fig 4C). Previously Stat5b was shown to be critical downstream of the IL7 receptor for commitment of lymphoid progenitors to the B cell lineage[29].

### MiR-24 target Trib3 regulates hematopoietic gene expression networks

Exogenous expression of Ebf1 in hematopoietic progenitors increases B-cell development and antagonizes myeloid promoting transcription factors C/EBPα and PU.1.[16] Since Ebf1 is not a validated or predicted target of the mirn23a cluster, we examined the list of mirn23a downregulated genes in 70Z/ overexpressing lines that have the potential of regulating Ebf1 expression. Tribbles 3 (Trib3) was downregulated in the miR-24-2 expressing 70Z/3 cells as determined by microarray analysis, and has previously been shown to be a miR-24-2 target in vascular smooth muscle cells[30]. In addition, we previously observed that forced expression of Trib3 in OP9 cocultures results in increased B cell development at the expense of myeloid development, consistent with the phenotype of *mirn23a*^*-/-*^ mice. Trib3 has the potential to regulate Ebf1 through negative regulation of Akt[31], which antagonizes nuclear forkhead box protein O1 (FoxO1)[31]. FoxO1 has previously been shown to drive Ebf11 expression in hematopoietic cells, which is necessary for early B-cell maturation and peripheral immune cell function[15,31,32]. Trib3 also has the potential to regulate B cell development through negative regulation of Smurf1 and the BMP/SMAD pathway, as BMP4 promotes B cell development in ex vivo cultures[33]. Through these mechanisms, we hypothesized that mirn23a regulation of Trib3 could help sustain commitment to the B cell lineage (as opposed to T cell/ NK cell) downstream of the MPP.

**Fig 5:**
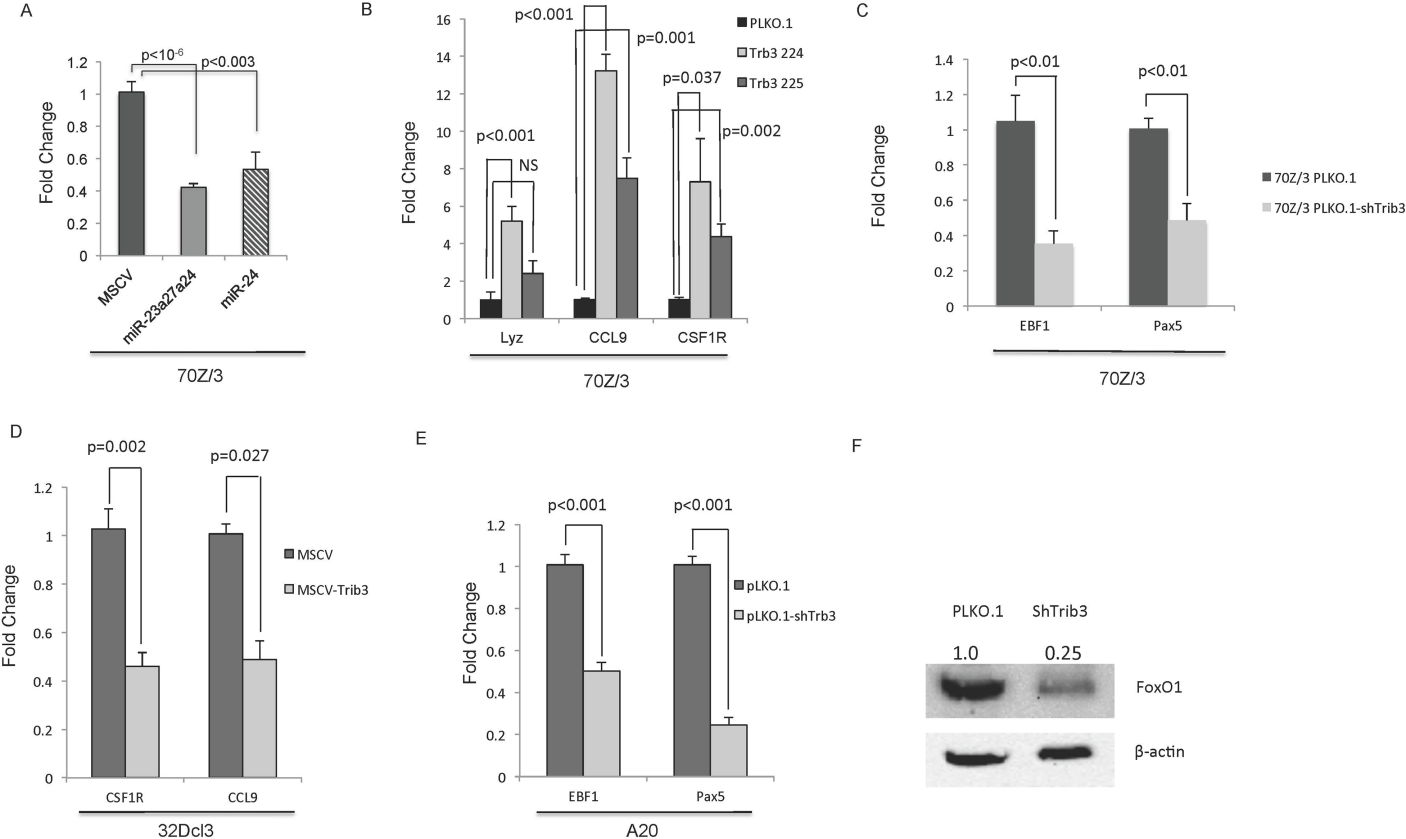
MiR-24 target Trib3 regulates hematopoietic gene expression networks. **A)** RNA from 70Z/3 Pre-B cells overexpressing MSCV, MSCV-miR-23a27a24, or MSCV-miR24 retrovirus was analyzed by qRTPCR for expression of miR-24 target Trib3. **B)** RNA was prepared from 70Z/3 cells expressing control (pLKO.1) or Trib3 shRNA knockdown (pLKO.1-shTrib3) vectors and analyzed by qRTPCR for myeloid specific gene expression. Myeloid genes Lyz, Ccl9, and Csf1r were all significantly increased in Trib3 knockdown lines. **C)** B cell genes EBF1 and Pax5 were significantly decreased in 70Z/3 cells with Trib3 knockdown. **D)** Trib3 was overexpressed in 32Dcl3 cells and analyzed by qRTPCR for expression of myeloid specific genes. Myeloid genes Csf1r and Ccl9 were significantly decreased when Trib3 is overexpressed **E**) A20 cells were transduced with control (pLKO.1) or shRNA knockdown (pLKO.1-shTrib3) of Trib3 and analyzed by qRTPCR for expression of B cell specific genes EBF1 and Pax5. EBF1 and Pax5 were significantly downregulated in ShTrb3 knockdown cells **F)** Trib3 knockdown in A20 cells resulted in decreased FoxO1 expression by immunoblot analysis. Statistical analysis was performed by unpaired students t-test.

We validated that miR-24-2 and *mirn23a* cluster expression in 70Z/3 cells decreases Trib3 expression using qRTPCR (Fig 5A). To examine if Trib3 alone had the potential to regulate myeloid gene expression networks, we generated 70Z/3 pLKO.1 control or pLKO.1-shTRIB3 knockdown cells and isolated RNA for qRTPCR analysis. These cells have 4–5 fold knockdown of Trib3 expression (S7A Fig). This revealed significantly increased expression of myeloid genes Lyz, Ccl9, and Csf1R, similar to what was observed with *mirn23a* overexpression (Fig 5B). Examination of B cell genes in these cells revealed decreased expression of Ebf1 and Pax5 (Fig 5C). To examine whether overexpression of Trib3 could lead to decreased myeloid gene expression, we switched to the 32Dcl myeloid cell line that has high levels of endogenous myeloid gene expression, making decreases in myeloid gene expression more readily observable (S7B Fig) Overexpression of Trib3 in 32D myeloid cells resulted in an ~2 fold reduction in Csf1R and Ccl9 expression, while Lyz did not amplify (Fig 5D).

We next wanted to confirm whether Trib3 could regulate essential B cell gene expression networks in an additional cell line, so we chose the A20 B lymphoma cell line since A20 cells express high levels of endogenous Ebf1 and Pax5. A20 pLKO.1-shTrib3 cells were generated and showed a ~3 fold decrease in Trib3 expression (S7C Fig). Consistent with *mirn23a* overexpression leading to decreased B cell gene expression, knockdown of Trib3 results in a >2-fold reduction in Ebf1 and Pax5 (Fig 5E). To examine the effect of Trib3 knockdown on FoxO1 protein expression in these cells, we performed immunoblots and observed decreased expression of FoxO1 in shTrib3 samples (Fig 5F). Together, these results suggest that miR-24 target Trib3 regulates immune cell gene expression networks that sustain lymphoid commitment while repressing myeloid development in mice.

### BMP/SMAD and AKT/FOXO1 signaling is critical for mirn23a mediated immune cell regulation

Trib3 has previously been shown to negatively regulate Smurf1, an E3 ubiquitin ligase capable of targeting SMAD1/5 and antagonizing the BMP/SMAD pathway[34]. Activation of the BMP/SMAD pathway has previously been shown to promote B cell development in ex vivo cultures[33]. To examine whether *mirn23a* expression affects Smurf1 protein levels in hematopoietic cells, we performed immunoblot analysis on *mirn23a*^*+/+*^ and *mirn23a*^*-/-*^ primary EML cells and observed decreased Smurf1 protein in *mirn23a*^*-/-*^ cells (S8A Fig). As expected decreased Smurf1 levels correlated with an increase in SMAD5 levels, while SMAD1 expression is not detectable in these cells (S8B Fig). As discussed above, *mirn23a* also has the potential to regulate immune cell development through the AKT/FoxO1 pathway, which is directly targeted by Trib3. Analysis of FoxO1 protein expression by immunoblot in primary EML cells revealed increased FoxO1 expression in *mirn23a*^-/-^ EMLs (S8C Fig). *Mirn23a*^*-/-*^ EMLs also have decreased expression of active p-AKT (inactivates FoxO1 through phosphorylation) and p-FoxO1 (the inactive cytoplasmic form) (S8D and S8E Fig).

**Fig 6:**
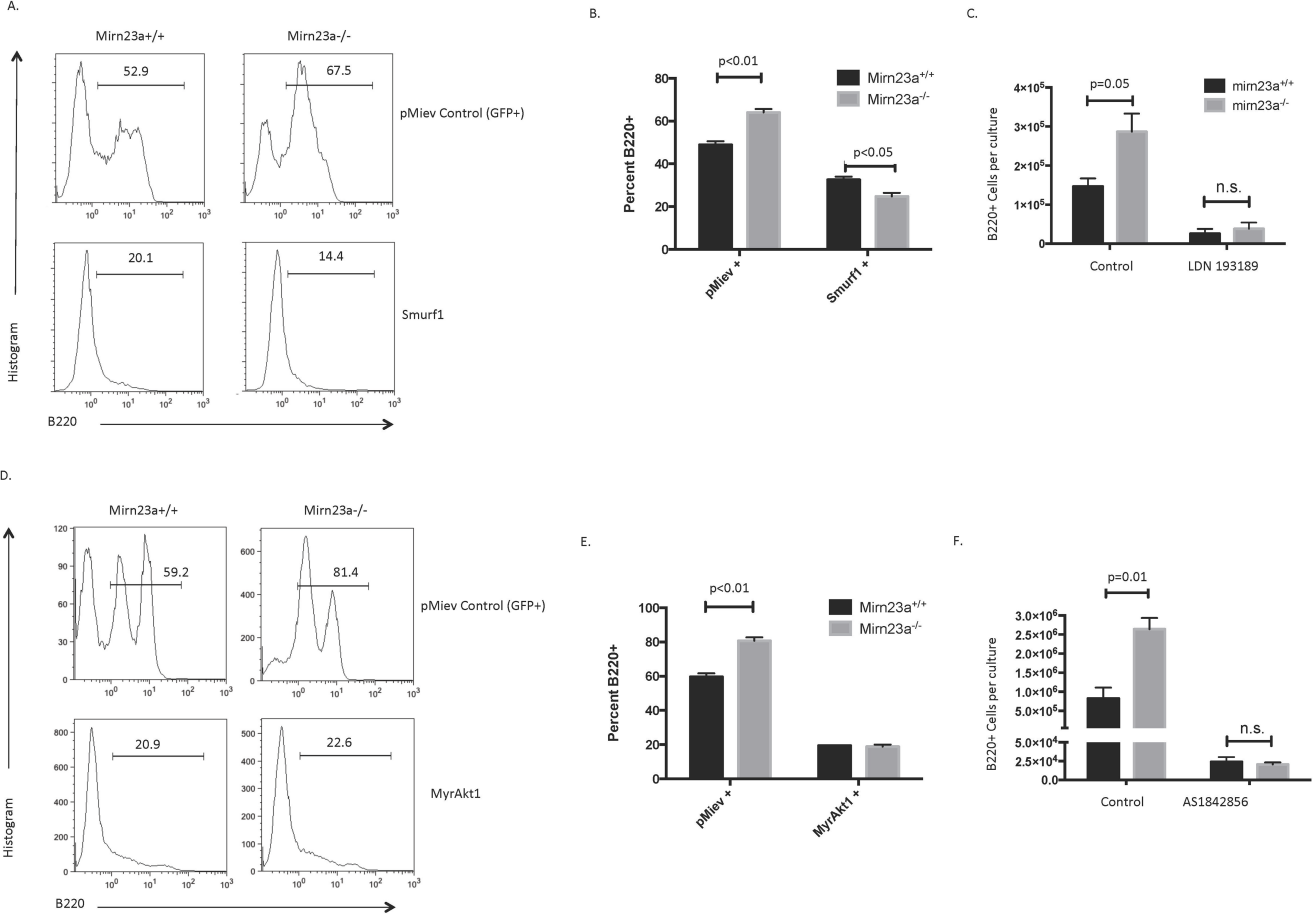
BMP/SMAD and AKT/FoxO1 signaling is critical for mirn23a mediated immune cell regulation. **A)** Primary lineage negative wildtype and mirn23a^-/-^ cells were transduced with empty vector control or Smurf1 and plated on OP9 cells with IL7 and Flt3L to evaluate the effect of Smurf1 overexpression on B cell development. Mirn23a^-/-^ cells show increased B cell development when transduced with empty vector control, while overexpression of Smurf1 results in decreased overall B cell development in both cultures, but abrogates the increased B cell development observed in mirn23a^-/-^ cultures. Representative plots are shown. **B)** The increased B cell development in mirn23a^-/-^ cultures transduced with empty vector control was statistically significant, while no difference was observed in Smurf1 overexpressing cultures. **C)** Primary Lin- hematopoietic cells from wildtype and mirn23a-/- mice were cocultured on OP9 stromal cells in the presence of IL-7, Flt3L, and with or without BMP inhibitor LDN 193189. B cells were significantly increased in mirn23a-/- cultures with DMSO vehicle control, while no difference was observed with the addition of BMP inhibitor. **D)** Similar to A, primary lineage negative cells were transduced with empty vector control or a constitutively active MyrAkt1. Overexpression of MyrAkt1 decreased B cell development and also abrogated the effect of mirn23a loss. Representative plots are shown. **E)** The increased B cell development was statistically significant in empty vector transduced cultures, while no difference was observed in MyrAkt1 cultures. **F)** OP9 cultures were performed as described in C, except with the addition of FoxO1 inhibitor AS1842856 and analyzed after 9 days of culture. B cells were significantly increased with vehicle control, while no difference was observed with FoxO1 inhibitor. Statistical analysis was performed by unpaired students t-test.

To functionally test whether these pathways were critical contributors to the *mirn23a* knockout mouse phenotype, we isolated primary Lin- cells from the BM of wildtype and *mirn23a*^*-/-*^ mice and transduced them with pMIEV empty vector (EV) control or Smurf1 overexpressing retrovirus. We then cocultured these cells on OP9 stromal cells for 9d with IL-7 and Flt3L. These conditions promote the growth of pro-B-cells from hematopoietic progenitors under normal conditions[35]. By 9d, there is a mix of both myeloid and lymphoid cell. Culturing Lin- hematopoietic cells with pMIEV EV control for 9d shows that *mirn23a* null cells have significantly increased B cell development when compared to wildtype cells (Fig 6A and 6B). However, overexpression of Smurf1 in these cells results in decreased overall B cell development and negates the phenotype of increased B cells in mirn23a^-/-^ cultures (Fig 6A and 6B). Additionally, directly antagonizing the BMP pathway with the LDN193189 BMP inhibitor during OP9 coculture (with no retroviral inserts) results in decreased B-cell development and similar numbers of B220^+^ B cells in both wildtype and *mirn23a*^*-/-*^ cultures, showing that inhibition of the BMP pathway abrogates the increased B cell development phenotype seen in the absence of mirn23a (Fig 6C). This suggests that BMP signaling is critical for both normal lymphoid development and the enhanced lymphoid development in *mirn23a*^*-/-*^ mice.

To test whether AKT/FoxO1 signaling contributes to the *mirn23a*-mediated phenotypes, we overexpressed a constitutively active AKT (myrAKT) in primary Lin- cells and observed B cell differentiation after 9d of OP9 coculture. In the absence of mirn23a, Trib3 levels rise repressing AKT activity leading to accumulation of FoxO1 in the nucleus. Expressing myrAKT was expected to reverse the stabilization of FoxO1 in *mirn23a*^*-/-*^ cells. Like Smurf1 overexpression, this analysis revealed that overexpression of activated AKT decreases B cell development and abrogates the effect of mirn23a loss on B cell development (Fig 6D and 6E). We conducted similar experiments with FoxO1 dominant negative (DN)[36] overexpression, but the effect of this overexpression was almost a complete block in B cell differentiation in both wildtype and mutant cultures. However, we performed OP9 cocultures with primary cells in the presence of DMSO or pharmacological FoxO1 inhibitor AS1842856, which revealed that FOXO1 inhibition also abrogates the increased B cell development observed in *mirn23a*^*-/-*^ cultures (Fig 6E and 6F). Together, these results suggest that the BMP/SMAD pathway and the AKT/FoxO1 pathway are both critical for *mirn23a’s* effect on B cell development.

### Essential B cell transcription factors bind to the mirn23a promoter and repress activity

**Fig 7:**
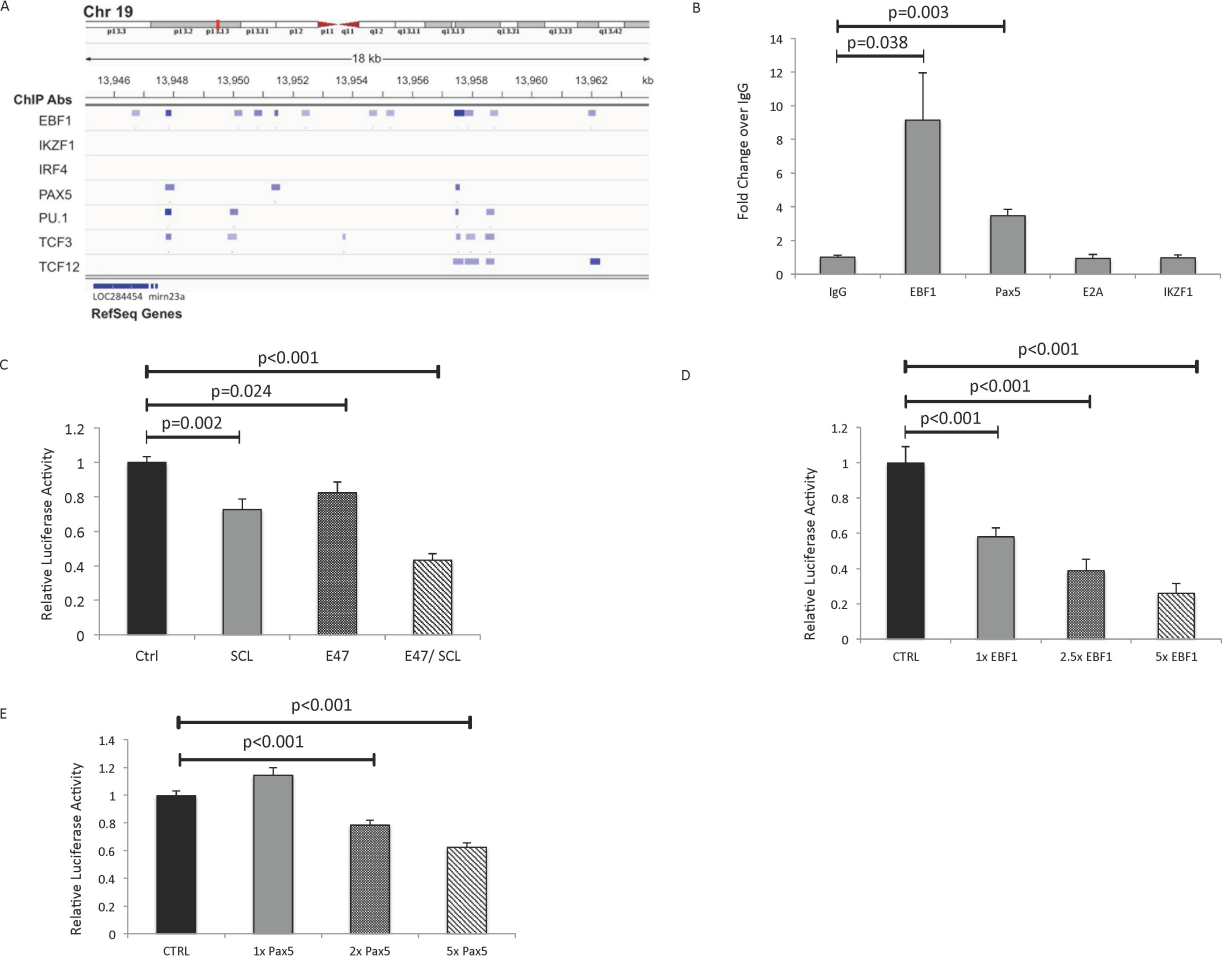
Essential B cell transcription factors bind to the mirn23a promoter and repress activity. **A)** Analysis of the ENCODE ChIP-seq database for B cell transcription factor binding to mirn23a gene regulatory regions showed binding of EBF1, PAX5, PU.1, TCF3, and TCF12 to regulatory elements of the mirn23a gene, while other transcription factors (such as IKZF1 and IRF4) did not interact with the mirn23a gene. Base pair positions refer to Human Feb. 2009 (GRCh37/hg19) Genome Assembly. **B)** Confirmation of DNA-protein interactions was performed by chromatin IPs in A20 cells. EBF1 and Pax5 binding to the mirn23a upstream region was significantly increased over IgG control, while no difference was observed for E2A. **C)** An 888 promoter fragment from the mirn23a promoter was cloned into a luciferase reporter construct and transfected with E47 proteins SCL, E47, or E47/SCL combined. Relative luciferase activity was measured. The same 888 bp construct was used to evaluate the effect of increasing concentrations of **D)** EBF1 or **E)** PAX5 on the mirn23a promoter. Experiments were performed in triplicate and statistical analysis was performed by unpaired students t-test.

We previously observed that *mirn23a* gene expression is positively regulated by the transcription factor PU.1. We reasoned that if *mirn23a* repressed B cell development, it should be a target for repression by B cell specific transcription factors. Examining ENCODE ChIP-seq[37] data for binding of B cell transcription factors binding to the *mirn23a* locus revealed that E2A (Tcf3), EBF1, and PAX5 associated with the *mirn23a* locus, whereas other factor such as IRF4 and IKZF1 are not detected at the locus (Fig 7A). Additionally, a ChIP on chip experiment with a chromosome 19 array had identified a conserved region upstream of *mirn23a* that bound the E2A related transcription factor Heb (Tcf12)[38]. To confirm binding to the mirn23a promoter, we collected chromatin from A20 cells and performed ChIPs with pulldowns for IgG (negative control), EBF1, E2A, PAX5, and IKZF1 (additional negative control). Analysis by qRTPCR revealed increased binding of EBF1 and PAX5 over IgG control, while no differences were observed in E2A or Ikzf1 binding (Fig 7B).

In addition, we examined the ability of these B cell transcription factors to regulate mirn23a regulatory regions in luciferase reporter assays. We generated a luciferase reporter plasmid with an 888bp murine *mirn23a* promoter fragment cloned upstream of the luciferase gene transcription start site. In addition to examine E2A regulation of mirn23a we isolated a conserved upstream region of mirn23a reported to bind HEB and E2A, and cloned it upstream of the mirn23 promoter in the luciferase reporter plasmid. In transient transfections, E2A protein E47 had a modest effect on activity of the luciferase reporter containing both the promoter and conserved upstream element with a reduction of ~20% (Fig 7C). E2A protein E47 can heterodimerize with the transcription factor SCL[39]. The two proteins are co-expressed together in early B cell progenitors where downregulation of *mirn23a* would be necessary[40]. SCL interacts with the corepressor ETO2, which allows it to repress gene transcription[41]. Coexpressing E47 and SCL had an enhanced effect on repression of *mirn23a* regulation with an ~50% reduction in reporter activity. SCL alone generated similar results as expression of E47 alone.

The effects of E47/SCL were rather modest so we also tested B cell factors EBF1 and PAX5. Both PAX5 and EBF1 are implicated in repressing myeloid gene expression in developing B lymphocytes[16,27]. Transient transfections were performed with a reporter gene regulated by only the 888bp *mirn23a* reporter. Increasing amounts of Ebf1 plasmid in transient transfections resulted in a graded decrease of *mirn23a* promoter activity (Fig 7D), whereas increasing Pax5 plasmid had a more modest effect on activity (Fig 7E).

### EBF1 negatively regulates mirn23a gene expression

**Fig 8:**
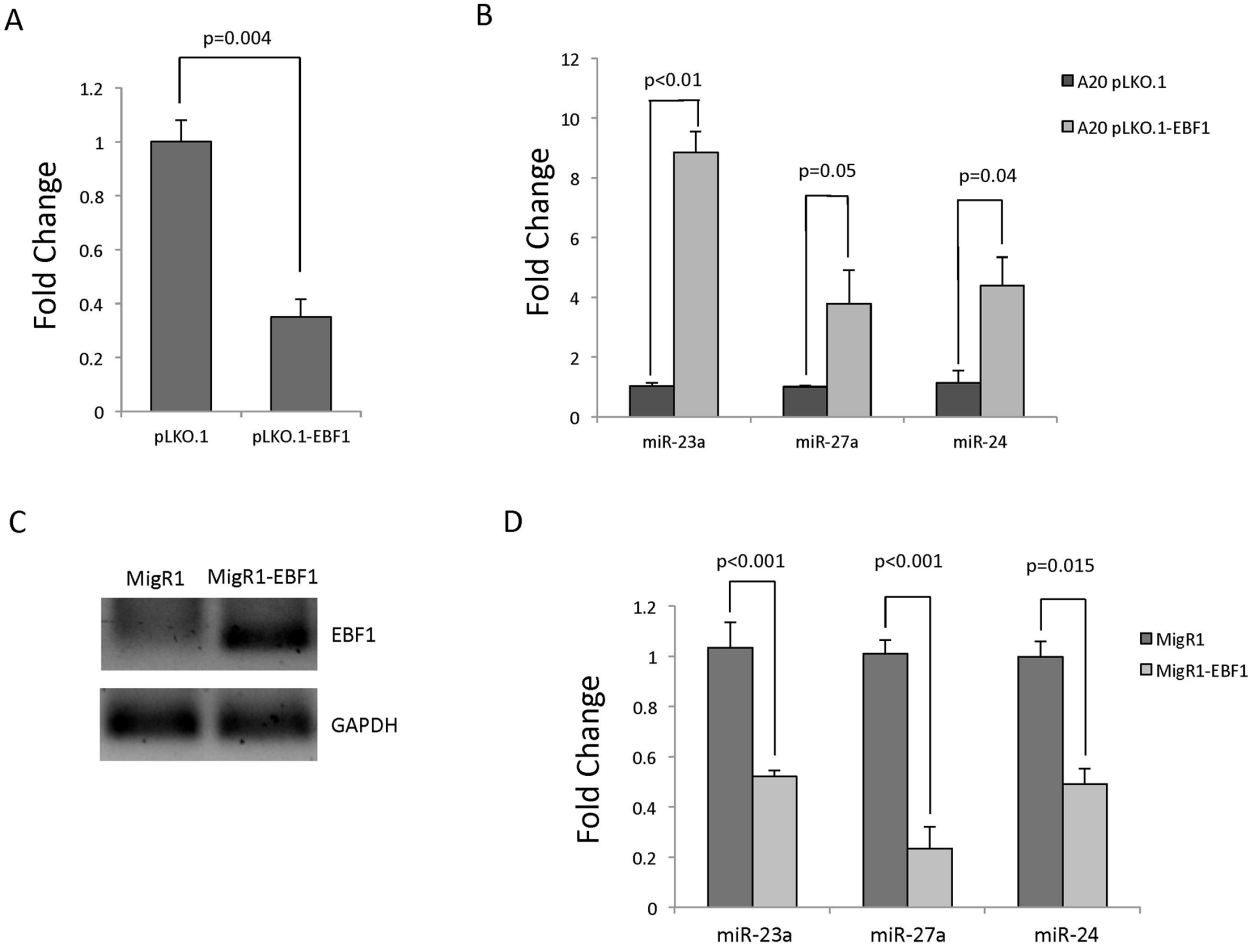
EBF1 negatively regulates mirn23a gene expression. RNA was collected from A20 cells stably transduced with control (pLKO.1) or EBF1 shRNA knockdown (pLKO.1-shEBF1). **A)** EBF1 knockdown was confirmed by qRTPCR. **B)** *Mirn23a* cluster expression was analyzed by qRTPCR. **C)** NIH/3T3 cells were transiently transfected with control (MigR1) or EBF1 overexpressing (Mig8-EBF1) plasmid DNA for 72 hours. RNA was then harvested and EBF1 overexpression was confirmed by RT-PCR. **D)** *Mirn23a* cluster expression was analyzed by qRTPCR. Statistical analysis done by unpaired students t-test.

Since EBF1 had the strongest effect in transient transfection, we followed up this observation by examining the effects on endogenous *mirn23a* expression when EBF1 levels were modified. We knocked down EBF1 in A20 B lymphoma cells using lentiviral shRNA and collected RNA for analysis by qRTPCR. A20 cells expressing shEBF1 had ~30% expression of control Ebf1 levels (Fig 8A). Knockdown of EBF1 resulted in significantly increased expression of miR-23a, miR-24, and miR-27a, consistent with EBF1 negatively regulating *mirn23a* (Fig 8B). To evaluate whether overexpression of EBF1 leads to decreased expression of *mirn23a* miRNAs, we transiently transfected EBF1 into NIH/3T3 fibroblasts and collected RNA 48hr post transfection. Overexpression of Ebf1 in transfected cells was confirmed by RT-PCR (Fig 8C). Cells overexpressing EBF1 showed a significant decrease in miR-23a, miR-27a, and miR-24 expression (Fig 8D). Taken together, these results show that EBF1 represses *mirn23a* transcription, creating a regulatory feedback loop between *mirn23a* and EBF1.

## Discussion

The miR-23a miRNA cluster promotes myeloid development at the expense of B cell development, as evidenced by overexpression and genetic knockout studies[20,42]. However, the pathways targeted by *mirn23a* that are critical to this process were previously unknown. In this study, we used a combination of bioinformatics, gene expression analysis, and functional primary cell studies to identify critical *mirn23a* targets and pathways that drive myeloid development at the expense of B cell development.

Our results revealed that the *mirn23a* miRNA cluster regulates several transcription factor genes in multipotent cells, including Runx1, Satb1, Ikzf1, and Bach1. For these gene expression studies, we utilized multipotent EML cell lines derived from wildtype and mirn23a^-/-^ mice, as well as primary sorted MPPs. While we observed 2–4 fold changes in target gene expression in the EML cell line model, this did not directly correlate with the primary MPP gene expression analysis. Increased expression of all transcription factors seen in the mutant EML cells were observed in mirn23a^-/-^ MPPs except for Ikzf1. However, the increases though significant, were only in the range of 1.4–1.6 fold. The less robust increase in gene expression observed in primary MPPs is likely due to the differentiation block in EML cell lines, which allows them to accumulate levels of essential transcription factors beyond what induces differentiation in vivo. The EML cells require strong differentiation promoting conditions, such as high levels of all trans retinoic acid for myeloid development or coculture with stromal cells with exogenously supplied cytokines for B cell development. However, it is important to note that these experiments, along with previous overexpression and knockout experiments with these lymphoid promoting transcription factors, do not define the precise levels of expression needed to direct differentiation.

While the precise levels needed to direct differentiation have not been established, we postulate that *mirn23a* miRNAs buffer the levels of these transcription factors in MPPs within a narrow threshold to influence differentiation, which is likely less than 2 fold changes in expression. This is based on growing evidence that modest differences in concentration of transcription factors have distinct effects on cellular differentiation, and thus, their levels of expression must be tightly controlled[43]. This was first shown with Oct-4 in embryonic stem cells where less than a 2-fold increase in protein would direct differentiation into primitive mesoderm and endoderm, whereas a decrease would lead to development of trophectoderm[44]. This showed that a precise level of Oct4 is required to maintain pluripotency. We have also previously showed the importance of levels of PU.1 in GMP cells in directing differentiation into monocytes and granulocytes[45]. Additionally, B cell promoting factors Bach1/2, Ikzf1, and Ebf1 have been shown to compete with myeloid factors to direct MPPs to differentiate into B cell progenitors, demonstrating that their concentrations relative to myeloid transcription factors is critical for B cell commitment[16,46–48]. Lastly, Satb1 is expressed in both HSCs and CLPs and is critical for the function of both cell types[11,49]. However, as mentioned previously, these studies do not delineate the precise level of lymphoid gene expression necessary for differentiation from multipotent progenitors. Currently, our model suggests that *mirn23a* miRNAs are part of a mechanism that maintains levels of B cell commitment factors to allow the cells to remain multipotent. In the absence of *mirn23a*, we postulate that normal fluctuations of protein levels are able to accumulate above a threshold level more often to commit MPPs to CLPs.

Previously we observed that the majority of the CLP population in *mirn23a*^*-/-*^ mice consists of B cell biased progenitors (BCPs)[20]. This bias toward B cells appears to be occurring through *mirn23a* inversely regulating 2 signaling pathways: PI3K/Akt and BMP/Smad. Trib3 modulation is in part responsible for this effect. Trib3 is an important downstream mediator of *mirn23a* as we previously reported that Trib3 overexpression could enhance in vitro B cell development similar to loss of *mirn23a*[20]. A recent genetic knockout study of Trib3 revealed no differences in myeloid vs lymphocyte composition, suggesting that Trib3 effects on B cell development are likely downstream of the MPP[50]. The B cell developmental populations, as well as B cell versus T cell cell fate decisions from the CLP stage, remain unknown in *Trib3*^*-/-*^ mice. This data suggests, however, that mirn23a regulation of Trib3 is likely critical in the CLP/Pro-B stage, while the initial differentiation from the MPP is dependent on transcription factors Bach1, Ikzf1, Satb1, and Runx1.

In addition, here we show that knockdown of Trib3 expression has similar effect on gene expression as overexpressing the *mirn23a* miRNAs (Fig 3). *Mirn23a* miRNA miR-24 targets Trib3, which represses Akt kinase and Smurf1 E3 ubiquitinase activity[20,30,31,34,51]. Trib3 repression of Smurf1 leads to increased levels of BMP regulated Smads 1 and 5 in vascular smooth muscle[34,52]. This regulation also occurs in hematopoietic cells as loss of *mirn23a* results in decreased levels of Smurf1 and increased levels of SMAD1 in EML cells (Fig 4). Consistent with repression of Akt, we observed an increase in the downstream-regulated protein FoxO1 in mutant cells. *Mirn23a* also regulates these pathways independent of Trib3. The PI3K/Akt pathway can be upregulated by *mirn23a* targeting the pathway inhibitors Pten (miR-23), and PPP2RSE (regulatory subunit of PP2A, miR-23)[53,54]. MiR-27a synergizes with Akt by targeting transcription factors FoxO1 (miR-27) that is repressed by Akt phosphorylation[55,56]. *Mirn23a* downregulates the BMP/Smad pathway through the targeting of the common Smad4 (mir-27a, miR-24), which is an obligate heterodimer partner for activated Smad1 and 5[57,58]. Additionally, miR-23a has been shown to target Smad5[59].

*Mirn23a*’s regulation of these pathways is consistent with the promotion of myelopoiesis over B lymphopoiesis. Several lines of evidence support a role for PI3K/Akt in promoting myelopoiesis. Pten deletion or expression of a dominant active Akt results in a decrease in lymphoid development and enhancement of monocyte/granulocyte development in vivo[60,61]. Additionally, deletion of the Akt-repressed targets FoxO1, O3, and O4 in HSCs results in increased myeloid progenitors[62]. Two studies report a role for BMP/Smad signaling in the commitment of MPPs to the myeloid and lymphoid lineage. Overexpression of Smad7 (inhibitory Smad) in human hematopoietic progenitors blocks B cell development and enhances monocyte/ granulocyte development similar to *mirn23a* overexpression[63]. Secondly OP9 cells support in vitro B cell growth through secretion of BMP4[33]. Our inhibitor studies suggest that both pathways are critical to mediating the differentiation phenotype observed in *mirn23a*^*-/-*^ mice. Absence of *mirn23a* results in decreased Akt activity and increased FoxO1. Treatment of wildtype and *mirn23a*^*-/-*^ cells with FoxO1 inhibitor or myrAKT (phosphorylates FoxO1 to reduce nuclear accumulation) results in an ablation of the differences in B lymphopoiesis seen in OP9 cultures (Fig 4). Similarly, inhibiting BMP signaling with LDN193189 or Smurf1 E3 Ubiquitinase expression ablates the differences in lymphoid development versus myeloid development in wildtype versus *mirn23a*^*-/-*^ cultures. This study adds to the mounting evidence that BMP signaling is critical for early B lymphopoiesis[33,63]. It will be important to address the molecular mechanisms regulated by SMAD transcription factors during adult B lymphopoiesis and examine whether FOXO1 and SMAD1/5 cooperate to regulate B lineage genes.

Comparing the MPP populations between wildtype and mutant mice, we were expecting that the lymphoid biased MPP4 population would be increased in the mutant bone marrow, but were surprised to observe that the myeloid biased MPP3 population was enhanced and the MPP4 population decreased in the mutants (Fig 1). However, the in vitro and in vivo functional results with pooled MPPs, as well as molecular data from EML cells and primary MPPs, may explain these findings. Loss of *mirn23a* may be increasing MPP4 polarization through increased levels of key transcription factors while simultaneously decreasing Akt activity, leading to increased nuclear FoxO1 accumulation, which stimulates the transcription of B cell fate determinant Ebf1. This may be speeding up differentiation of the MMP4 to B cell biased progenitors, making the MPP4 population unstable. Similarly, higher levels of transcription factor Bach1, which inhibits myeloid genes, may be slowing down the differentiation of the MPP3 cell to the myeloid lineage, explaining their accumulation in the mutant mouse. The accumulation of MPP3 may become more pronounced as the mouse tries to compensate for the decreased mature myeloid population. Consistent with the idea that differentiation of MPP3 is slowed down, we observed an increase in the population of cells not expressing myeloid or lymphoid markers in the OP9 cultures of *mirn23a*^*-/-*^ MPPs in the early stages of these cultures when myeloid development is predominant(Fig 2). This interpretation would explain why transplanted MPPs isolated from *mirn23a*^*-/-*^ mice have increased contribution to the B lymphoid lineage compared to wildtype MPPs although initially consisting of more myeloid biased MPP3s.

Lastly, the importance of *mirn23a* in commitment of progenitors to the myeloid lineages is underscored by the negative transcriptional regulation of *mirn23a* by key B cell transcription factors. If *mirn23a* regulates myeloid commitment through repression of B cell specific genes, one would expect that it would be repressed by B cell promoting factors. Analysis of ChIP-seq data from the ENCODE project demonstrated that E2A, EBF1, and PAX5 are associated with the promoter and conserved regions of the *mirn23a* locus. This was confirmed by ChIP assays in this report. Furthermore, transient transfection analysis demonstrated that E2A, EBF1, and PAX5 all could repress transcriptional activity with EBF1 having the most potent effect. Both overexpression and underexpression of EBF1 confirmed its ability to negatively regulate the endogenous *mirn23a* gene.

Overall, this study shows that *mirn23a* regulates several complex pathways to drive immune cell fate decisions in the mouse. In the MPP, many critical factors are targeted by all members of the *mirn23a* cluster. However, sustained commitment to the lymphoid lineage appears heavily dependent on miR-24 and its direct target Trib3. Trib3 modulates this sustained commitment by regulation of both the AKT/FoxO1 and BMP/SMAD pathways, which appear to synergistically work together to reinforce B cell commitment. The inverse regulation by *mirn23a* of the PI3K/Akt and BMP/ SMAD pathways is intriguing and may be also of great importance in other developmental systems.

The *mirn23a* regulation of lymphoid and myeloid gene expression has potentially useful medical implications. MiRNAs appear to be promising therapeutic targets based on their small size, high conservation, and ability to manipulate gene expression in a physiological range[64]. Based on its role in immune cell development, *mirn23a* mimics and antagonists may be used to treat disorders such as cytopenias, immune deficiencies, and hematological malignancies. Understanding the role of miRNAs in hematopoiesis and their mechanism of action will be critical to manipulating miRNA expression in the treatment of human disease.

## Methods

### Cell culture

70Z/3 and A20 cell lines were obtained from ATCC (Manassas, VA). 32Dcl3 cells were a gift from Allan Friedman (Johns Hopkins). Unless stated otherwise, cell culture media and additives were obtained from Invitrogen (Carlsbad, CA). 70Z/3 cells were grown in RPMI-1640 media (Sigma, St. Louis, MO) supplemented with 10% FBS, 0.1 mM glutamax, 10 mM HEPES, 1 mM sodium pyruvate, and 55 uM 2-Mercaptoethanol (BME). A20 cells were grown in RPMI supplemented with 10% FBS and 55uM BME. 32Dcl3 cells were cultured in IMDM supplemented with 10% FBS, 10% Wehi-3B conditioned media and 55μM BME. Generation of cell lines overexpressing *mirn23a* miRNAs was described previously[65]. All media contained 50 U/ml penicillin and 50mg/ml streptomycin.

### Generation of primary EML cell lines from mouse bone marrow

Wildtype and *mirn23a*^*-/-*^ C57BL/6 mice were intraperitoneally injected with 5mg 5-fluorouracil in 100 uL 1X phosphate buffered saline (PBS). Bone marrow was harvested 3 days later and red blood cells were removed by ammonium chloride lysis. Nucleated blood cells were cultured in IMDM supplemented with 20% horse serum, 10% HM3 conditioned media (as a source of GM-CSF), 20ng/mL human IL-6, and 10 ng/mL murine IL-1b. Recombinant mouse cytokines obtained from R&D Systems (Minneapolis, MN, USA) or Invitrogen. Cells were then spin transduced with RARα403 from supernatants of GP + E86 producer lines for 2 hours at 32 degrees Celsius at 3200 RPM with 5ug/mL polybrene. Following spin transduction, cells were transferred to IMDM supplemented with 20% horse serum, 10% KSL CM (as a source of SCF), 10% WEHI-3B (as a source of IL-3), and 8 U/mL human erythropoietin. Cells were then passaged every 2–3 days in IMDM supplemented with 20% horse serum, 10% COS-KSL (as a source of SCF). Cells were selected for neomycin resistance by treating cells with G418 for 10 days and continuing cultures with the live cells. EML phenotype was confirmed by flow cytometry.

### EML differentiation assays

Multipotent EML cell lines were generated from wildtype and *mirn23a*^*-/-*^ mice as previously described[19,66]. These cells were differentiated to the myeloid lineage by plating 50,000 cells/mL in EML media (IMDM supplemented with 20% horse serum, 10% COS-KSL) supplemented with 10uM ATRA and 10% WEHI-3B conditioned media for 48h. Following 48h, cells were washed out of WEHI-3B conditioned media and replated with EML media supplemented with 10uM ATRA and 10% HM5 conditioned media for 5 more days. Cells were analyzed on day 7 for CD11b cell surface expression. For B cell differentiations, EML cells were plated onto 30,000 OP9 cells in EML media supplemented with 2ng/mL IL7 and 10ng/mL Flt3L. Cells were passaged onto fresh OP9 every 3 days and B cell differentiation was analyzed 7 days after culture.

### Retroviral/ lentiviral transduction

Construction of MSCV-23a, 27a, 24–2, and mirn23a cluster retroviral plasmids were previously described[3]. MSCV-myrAKT[60], pBABE-FOXO1dn[36], and pRL-Smurf1[67] were obtained from Addgene. pMIEV-Smurf1 was generated by subcloning the Smurf1 cDNA from pRL-Smurf1 to the MSCV-based retroviral vector pMIEV which co-expresses GFP. Retroviruses were generated by co-transfecting 293FT cells with the retroviral plasmid along with retroviral packaging vector pCL-Eco (Imgenex, San Diego, CA) using Lipofectamine 2000 (Invitrogen, Carlsbad, CA). Lentiviral shRNA vectors pLKO.1, pLKO.1-shTrib3, and pLKO.1shEBF1 (Dharmacon, Inc., Lafayette, CO) were co transfected into 293FT cells with pVSV-G, pMDL, and pRSV-REV plasmids (National Gene Vector Biorepository, Indianapolis, IN) using a Calcium Phosphate Transfection Kit according to manufacturer’s instructions (Invitrogen, Carlsbad, CA). For both retroviral and lentiviral preparations, 48h and 72 h post-transfection viral supernatants were harvested and concentrated using Centricon Plus-70 filters (Millipore, Billerica, MA). Cell lines were transduced by spin transduction for 2 hours at 32 degrees Celsius at 3200 RPM with 5μg/mL polybrene. Stable cell lines were generated by sorting for GFP+ cells or selecting for puromycin resistance. pBabe DN-FOXO1-HA neo was a gift from Kevin Janes (Addgene plasmid # 45814). pRK-Myc-Smurf1 was a gift from Ying Zhang (Addgene plasmid # 13676). pMSCV-flag-myr-Akt1-IRES-GFP was a gift from Kira Gritsman & Jean Zhao (Addgene plasmid # 65063).

### Transfection

NIH/3T3 fibroblasts (5x10^5^) were plated onto 10cm plates the night before transfection. Cells were then treated with Lipofectamine LTX (Invitrogen, Carlsbad, CA) transfection reagent with the addition of 10μg MigR1-GFP or Mig8-EBF1 DNA, along with 5μg of Helper II DNA. Cells were cultured in Opti-MEM media overnight and replaced with DMEM media with 10% FBS the following day. Transfection efficiency was assessed by expression of GFP under a fluorescent microscope and RNA was collected 72 hours post transfection. RNA was collected by TRIzol extraction and used in subsequent qRTPCR assays.

### Ex vivo hematopoietic OP9 cultures

Nucleated cells from the femur and tibia of 5–6 week old mice were lineage depleted with a MACS lineage cell separation kit according to manufacturer’s instructions (Miltenyi Biotec, Auburn, CA). Lineage depleted cells were cultured onto 30,000 OP9 cells (plated night before) in IMDM supplemented with 10% defined FBS, 55mM BME, 50 U/ml penicillin, 50mg/mL streptomycin, 0.1mM Glutamax, 5ng/mL Flt3L, and 1 ng/mL IL-7. Cultures were treated with DMSO control, 1 uM of BMP inhibitor LDN 193189 (Abcam, Cambridge, MA), or 100nM FoxO1 inhibitor AS1842856 (Calbiochem, Billerica, MA). Mouse cytokines were obtained from R&D Systems or Invitrogen. Cells were transferred onto fresh OP9 cells every 3 days. To evaluate myeloid and B-cell differentiation in cultures, cells were analyzed 6 or 9 days after culture with B220-APC and CD11b-APC/cy7 antibodies (BioLegend, San Diego, CA) by flow cytometry on the Beckman Coulter FC500 flow cytometer.

### Microarray analyses

Independently derived 70Z/3 lines infected with MSCV, or MSCV-miR-24-2 were generated previously[65]. Four MSCV lines and 2 MSCV-miR-24-2 lines were examined. Total RNA was prepared using Trizol reagent (Invitrogen, Carlsbad, CA). RNA quality was evaluated using the Lab-on-a-Chip Bioanalyzer 2100 (Agilent, Palo Alto, CA). Biotinylated cRNA was prepared according to the standard Affymetrix protocol using the Superscript Choice System (Invitrogen) and the RNA transcript labeling kit (ENZO, Farmingdale, NY) for cRNA preparation. Following fragmentation 10ug of cRNA was hybridized to Affymetrix Mouse 430 2.0 Gene Chip. Microarray analysis was performed using the Affymetrix GeneArray Scanner G2500A. The Affymetrix Expression Console Software Version 1.4.0 was used to create summarized expression values (CHP-files) from expression array feature intensities (CEL-files). Raw data were normalized with robust multichip analysis (RMA) with Affymetrix transcriptome analysis console (TAC) software. DNA microarray and sample annotation data were deposited in GEO under the accession number GSE65874.

### Quantitative reverse-transcriptase PCR (qRTPCR)

Total RNA was isolated from in vitro cell lines using TRIzol (Invitrogen, Carlsbad, CA) according to the manufacturer’s protocol. Complementary DNA (cDNA) was reverse transcribed from 1ug of RNA using Taqman microRNA reverse transcription kit according to manufacturer's protocol (Applied Biosystems, Carlsbad, CA). Quantitative analysis was performed using gene specific Taqman (Applied Biosystems, Foster City, CA) or SYBR Green (Applied Biosystems) reagents. All experiments were performed in triplicate using BioRad CFX96 C1000 System (BioRad, Hercules, CA). Relative gene expression was calculated using the ΔΔCT method. GAPDH was used to normalize expression across different RNA preparations. Relative values are presented as SEM of three independent experiments.

### Generation of mirn23a luciferase reporter constructs

An 888bp fragment of the murine *mirn23a* promoter was amplified from NIH3T3 genomic DNA with PCR using the following primers: GAGCTCTAAACGTGAGCCACCAACTG and AAGCTTGCACAGGGTCAGTTGGAAAT. Restriction sites SacI and HindIII were included in the primers. PCR fragment was cloned into pCR2.1 with TOPO TA Cloning Kit (Invitrogen, Carlsbad, CA). The *mirn23a* promoter fragment was subcloned into the pGL3-basic luciferase (Promega) plasmid into the SacI and HindIII sites to generate pGL3-mirn23aPR. A putative upstream *mirn23a* enhancer was isolated by PCR using the primers: GGTACCCTGTGACTCAGCCTCATT and GGACACTTGTGGAAGCTGGA and was cloned into pCR2.1. A KpnI/ SacI fragment was isolated and subcloned into pGL3-mirn23aPR to generate pGL3-mirn23aPRcns.

### Luciferase assays

Putative transcription factors that regulate mirn23a were identified by examining ENCODE ChIP-seq data using the Integrative Genomic Viewer (IVG) software version 2.3.68[68]. All transient transfections of 293T cells were carried out in 24 well plates with Lipofectamine 2000 according to manufacturer’s instructions. For E47/ SCL assays, cells were co-transfected with 100ug pGL3-CNS14mirn23apr888, 350ug MigR1-E47 and/or 350ug pCAPP-SCL, and 5 ng pRL-tk. Total DNA content for each transfections was kept constant with 350ug of MigR1 and/or 350ug pCAPP. For EBF1 experiments, cells were co-transfected with 200ug pGL3-mirn23apr888, 0-750ng pSport-hEBF1, and 5ng tkRL. For each condition DNA content was kept constant with 0-750ng pcDNA3.1 (CMV promoter). For PAX5 experiments, cells were co-transfected with 200ug pGL3-mirn23apr888, 0-750ng MigR1-Pax5, and 5ng tkRL. For each condition DNA content was kept constant with 0-750ng MigR1. Each experimental condition was performed with 3 independent transfections. For all conditions, 48h post-transfection cell lysates were harvested using Promega cell lysis buffer. Firefly and renilla luciferase activity was measured using the Dual-Luciferase Assay System (Promega). Firefly luciferase values were normalized to renilla luciferase values in order to adjust for potential differences in transfection efficiency. For Bach1 3’ UTR luciferase assays, a LightSwitch reporter assay (Activ Motif, Carlsbad, CA) containing the human 3’ UTR of Bach1 was used. 1x10^5^ 293T cells were transfected in a 24 well plate with a LightSwitch control (no 3’ UTR) or experimental (with Bach1 3’ UTR) reporter, along with an empty vector miRNA control (MSCV), MSCV-miR23a27a24, or MSCV-miR23a. Cells were transfected using Lipofectamine 2000 according to manufacturer’s protocol. 48 hours after transfection, luciferase activity was determined using the MISSION LightSwitch luciferase assay reagent (Sigma Aldrich) kit according to manufacturer’s protocol.

### Immunoblotting

Whole-cell extracts were prepared by lysing cells in RIPA buffer (50 mM Tris pH 7.5, 150 mM NaCl, 1% NP-40, 0.5% EDTA, 0.1% SDS) and protease inhibitor cocktail (Roche). Protein concentration was determined using the Bicinchoninic Acid (BCA) protein assay (Pierce, Rockford, IL). 75 μg of whole cell lysates was separated by SDS-PAGE and transferred to nitrocellulose membrane. Membranes were blotted with the following antibodies: Ikzf, FoxO1, Smurf1, Smad1, Satb1, β-actin (Cell Signaling, Danvers, MA), Ebf1, and Runx1 (Santa Cruz, Dallas, TX). Horseradish peroxidase conjugated secondary antibodies were purchased from GE Healthcare UK Ltd (Buckinghamshire, England). Clarity Western ECL substrate enabled detection of antibodies (Thermo Scientific, Rockford, IL). Analysis was performed using BIORAD Chemidoc XRS+ System using Imager Lab Software (Hercules, CA) or ImageJ (NIH, Bethesda, MD).

### Chromatin immunoprecipitations (ChIPs)

ChIP assays were performed using Active Motif (Carlsbad, CA) ChIP-IT Express kit according to manufacturer’s protocol. A20 cells were grown in 15cm plates to ~80–90% confluency before crosslinking cells. Chromatin was sheared by sonication (10 pulses, 25% Amp, 20 seconds/pulse, with 30 seconds rest between pulses). IPs were done using antibodies against IgG (p120-101-Bethyl laboratories), E47 (clone G127-32 –BD Biosciences), EBF1 (Clone C-8, Santa Cruz Biotechnology), Pax5 (clone A-11, Santa Cruz Biotechnology), and Ikzf1 (clone H-100, Santa Cruz Biotechnology). Following crosslink reversal, relative DNA was analyzed by qRT-PCR. Primers (Probe: 5’-/56-FAM/CATTTGGCC/ZEN/TGCTTTGGGCTCAG/3lABkFQ/-3’ primer 1: 5’-CCTCCCTCAGCTTCCTCT-3’ primer 2: 5’-GCTTCCCACTCTGCTTCTATC-3’)were obtained from IDT (Coralville, Iowa).

### MPP transplants

CD45.1 recipient mice were irradiated by X-ray irradiation (1000 gy) the night before transplant. MPPs (LSK CD34+) were collected from CD45.2 wildtype and mirn23a^-/-^ mice through FACS sorting. 2000 MPP cells were retro-orbitally injected along with 2x10^5^ CD45.1 support marrow cells into the lethally irradiated CD45.1 recipient mice. Mice were sacrificed 4 weeks after transplant and analyzed by flow cytometry for contribution to the B cell (B220) and myeloid (CD11b) lineages.

### BrdU assays

Mice were injected with 2ug of BrdU (BD Biosciences, Billerica, MA) diluted in 200uL of sterile phosphate buffered saline by intraperitoneal injection 16 hours prior to analysis. Bone marrow cells were harvested from the femurs and tibias of these mice and cell fixation and permeabilization was done according to BD Pharmingen BrdU flow kit protocol (BD Biosciences, Billerica, MA). BrdU incorporation in HSPCs was evaluated by flow cytometry using a panel including Sca1 (D7)-FITC, Lineage cocktail- Biotin, Avidin-Texas Red, BrdU-APC, and c-Kit (2B8)-APC/Cy7.

### Annexin V/ 7AAD analysis

Primary bone marrow cells were harvested from the femurs and tibias of mice 5–6 weeks of age. Staining for annexin V and 7AAD was done according to the FITC Annexin V apoptosis detection kit with 7-AAD (Biolegend, San Diego, CA). Analysis by flow cytometry was done using a combination of antibodies including Annexin V- FITC, Sca1- PE, Lineage cocktail-biotin, TR-Avidin, 7AAD, and c-Kit-APC/Cy7.

### Statistical analysis

Statistical data are presented as the mean +/- standard error of the mean (SEM). Differences between sample groups were determined by performing an unpaired student t-test. Analysis was performed using PRISM software version 6.0 (Graphpad software).

### Ethics statement

The use of mice in these experiments was approved by the Indiana University School of Medicine and University of Notre Dame Institutional Animal Care and Use Committees (Protocols 13–017 and 16–022). Mice used for this study were euthanized using a commercial Euthanex CO2 machine, ensuring ethical euthanasia of all mice.

## Supporting information

S1 Table Genes significantly changed in miR-24 overexpressing 70Z/3 Pre-B Cells. Two unique MiR-24 overexpressing 70Z/3 cell lines were generated through limiting dilution along with a control line infected with empty retrovirus. Cell lines were analyzed for genome wide RNA expression by microarray analysis using Affymetrix Mouse Genome 430 2.0 Arrays. Genes differentially regulated >2 fold between control and miR-24 overexpressing cell lines are shown.
(PDF)

S2 Table Genes significantly changed in miR-23a overexpressing 70Z/3 Pre-B Cells. Two unique MiR-23a overexpressing 70Z/3 cell lines were generated through limiting dilution along with a control line infected with empty retrovirus. Cell lines were analyzed for genome wide RNA expression by microarray analysis using Affymetrix Mouse Genome 430 2.0 Arrays. Genes differentially regulated >2 fold between control and miR-23a overexpressing cell lines are shown.
(PDF)

S3 Table Genes significantly changed in miR-27a overexpressing 70Z/3 Pre-B Cells. Two unique MiR-24 overexpressing 70Z/3 cell lines were generated through limiting dilution along with a control line infected with empty retrovirus. Cell lines were analyzed for genome wide RNA expression by microarray analysis using Affymetrix Mouse Genome 430 2.0 Arrays. Genes differentially regulated >2 fold between control and miR-24 overexpressing cell lines are shown.
(PDF)

S1 Fig Mirn23a^-/-^ stem cells show impaired differentiation to the myeloid lineage. ) The differentiation potential of wildtype and *mirn23a*^*-/-*^ MPPs to the myeloid lineage was evaluated by sorting 100 MPP cells into a 96 well plate with OP9 stromal cells in the presence of IL-7 and Flt3L cytokines for 7 days. A) *Mirn23a*^*-/-*^ MPP wells show a significant increase in their undifferentiated stem cell population (B220^-^CD11b^-^) compared to wildtype MPPs after 8 days of culture. B) *Mirn23a*^*-/-*^ MPPs show a significant decrease in their differentiated myeloid cell population. Statistical analysis done by unpaired students t-test.
(TIFF)

S2 Fig Mirn23a^-/-^ LSK populations show no gross defects in proliferation of apoptosis. **A)** Wildtype and mirn23a^-/-^ mice were injected intraperitoneally with BrdU 16 hours prior to sacrifice. Bone marrow was then harvested and cells were fixed and permeabilized then analyzed for LSK and BrdU expression by FACS. Representative plots are shown. **B)** No significant differences were observed between wildtype and mirn23a^-/-^ mice. **C)** Bone marrow was harvested from wildtype and mirn23a^-/-^ mice and stained for LSK surface markers along with annexin V and 7AAD. Representative plots are shown. **D)** A slight decrease was observed in mirn23a^-/-^ mice that was statistically significant. Statistical analysis done by unpaired students t-test.
(TIFF)

S3 Fig Multipotent EML cell lines generated from wildtype and mirn23a^-/-^ mice express no committed lineage markers. **A)** EML cells derived from wildtype and *mirn23a*^*-/-*^ mice express stem cell markers and do not express any committed lineage markers.
(TIFF)

S4 Fig Generation of 70Z/3 overexpression lines and contribution of individual miRNAs to gene expression. **A)** 70Z/3 pre B cells were transduced with control, mirn23a cluster, or miR-24 alone overexpression vectors. MiR-24 expression was analyzed by qRT-PCR to validate overexpression. **B)** The contribution of each individual miRNA to myeloid gene expression was evaluated by qRTPCR. MiR-24 had the ability to upregulate critical myeloid genes Ccl9 and Csf1r. **C)** The contribution of each individual miRNA to lymphoid gene expression was evaluated by qRTPCR. All three miRNAs had the ability to significantly downregulate lymphoid gene expression. Statistical analysis done by unpaired students t-test.
(TIFF)

S5 Fig Heat map of top 50 upregulated and downregulated genes from microarray analysis. Two unique lines for each miRNA and a control line infected with empty retrovirus were generated in 70Z/3 cells and analyzed for genome wide RNA expression by microarray analysis using Affymetrix Mouse Genome 430 2.0 Arrays. A heat map of a ranked list of the top 50 upregulated and downregulated genes in the miRNA expressing 70Z/3 lines compared to the empty vector expressing lines is shown.
(TIFF)

S6 Fig Mirn23a regulates the B cell promoting IL2/Stat5 signaling pathway. **A)** A gene set enrichment analysis was performed on the microarray data from 70Z/3 cells overexpressing individual components of the mirn23a cluster. The enrichment plot for IL2/Stat5 signaling is shown. **B)** Results summary for IL2/Stat5 signaling from the gene set enrichment analysis. **C)** Heat map showing the individual components of the IL2/Stat5 signaling pathways affected by miR-23a, miR-24, or miR-27a expression.
(TIFF)

S7 Fig Generation of Trib3 overexpression and knockdown cell lines. **A)** 70Z/3 pre B cells were transduced with control or Trib3 shRNA knockdown vectors. Trib3 knockdown was confirmed by qRT-PCR. **B)** 32Dcl cells were transduced with control or Trib3 overexpression vectors. Confirmation of Trib3 overexpression was done using RT-PCR. **C)** A20 cells were transduced with control or Trib3 knockdown vectors. Confirmation of Trib3 knockdown was done using qRT-PCR. Statistical analysis done by unpaired students t-test.
(TIFF)

S8 Fig Mirn23a regulates critical components of the BMP/Smad and AKT/FoxO1 pathways. Whole cell lysates were collected from primary multipotent EML cell lines derived from wildtype and *mirn23a*^*-/-*^ mice. Numbers designate fold change in protein levels as determined by densitometry. **A)** Protein expression of Smurf1 was analyzed by immunoblot in wildtype and *mirn23a*^*-/-*^ EML cells. **B)** SMAD1 protein expression was analyzed by immunoblot in wildtype or *mirn23a*^*-/-*^ EMLs. **C)** FoxO1 protein expression was analyzed in wildtype and *mirn23a*^*-/-*^ EML cells. **D)** p-AKT protein expression was analyzed by immunoblot. **E)** p-FoxO1 protein expression was analyzed by immunoblot.
(TIFF)
