# Supplementary material for: The miR-23a~27a~24-2 microRNA cluster buffers transcription and signaling pathways during hematopoiesis
Source: PLoS Genet. 2017 Jul 13;13(7):e1006887. doi: 10.1371/journal.pgen.1006887 (PMC5531666; doi:10.1371/journal.pgen.1006887)
Supplement: S1 Table — Two unique MiR-24 overexpressing 70Z/3 cell lines were generated through limiting dilution along with a control line infected with empty retrovirus. Cell lines were analyzed for genome wide RNA expression by microarray analysis using Affymetrix Mouse Genome 430 2.0 Arrays. Genes differentially regulated >2 fold between control and miR-24 overexpressing cell lines are shown. (PDF) [file pgen.1006887.s001.pdf]

Supplementary Table 1. Genes significantly changed in miR-24 overexpressing 70Z/3 Pre B Cells

Transcript Cluster ID	Transcript ID	Fold Change	ANOVA p-value		Gene Symbol	Description
1419331_at	Mm.33402.1	5.69	0.00358	0.49	Cdh17	cadherin 17
1417936_at	Mm.2271.1	5.05	0.00005	0.16	Ccl9	chemokine (C-C motif) ligand 9
1448898_at	Mm.2271.1	4.99	0.00063	0.37	Ccl9	chemokine (C-C motif) ligand 9
1419219_at	Mm.137441.1	4.18	0.00021	0.37	Cyp4f18	cytochrome P450, family 4, subfamily f, polypeptide 18
1432826_a_at	Mm.89474.8	4.09	0.00934	0.57	Cd80	CD80 antigen
1454711_at	Mm.28367.1	3.43	0.04645	0.71	Trio	triple functional domain (PTPRF interacting)
1419872_at	Mm.201971.1	3.33	0.00039	0.37	Csf1r	colony stimulating factor 1 receptor
1438687_at	Mm.36534.1	3.23	0.00650	0.54	Lax1	lymphocyte transmembrane adaptor 1
1424542_at	Mm.3925.1	3.09	0.00142	0.43	S100a4	S100 calcium binding protein A4
1423754_at	Mm.141021.1	3.05	0.00055	0.37	Ifitm3	interferon induced transmembrane protein 3
1434322_at	Mm.89789.1	2.88	0.03772	0.69	Micall2	MICAL-like 2
1439189_at	Mm.215803.1	2.88	0.00172	0.43	Fnip2	folliculin interacting protein 2
1452217_at	Mm.203866.1	2.77	0.00140	0.43	Ahnak	AHNAK nucleoprotein (desmoyokin)
1451950_a_at	Mm.89474.5	2.57	0.02413	0.66	Cd80	CD80 antigen
1426936_at	Mm.220992.1	2.53	0.01845	0.63	BC005512	cDNA sequence BC005512; RIKEN cDNA F630007L15
1428372_at	Mm.1075.1	2.48	0.00893	0.57	St5	suppression of tumorigenicity 5
1431464_a_at	Mm.9699.2	2.46	0.01255	0.59	Pmm2	phosphomannomutase 2
1425214_at	Mm.32929.1	2.45	0.00414	0.50	P2ry6	pyrimidinergic receptor P2Y, G-protein coupled, 6
1421182_at	Mm.30700.1	2.44	0.02019	0.64	Clec1b	C-type lectin domain family 1, member b
1453304_s_at	Mm.204648.1	2.42	0.02820	0.67	Ly6e	lymphocyte antigen 6 complex, locus E
1426063_a_at	Mm.4362.2	2.36	0.02054	0.64	Gem	GTP binding protein (gene overexpressed in skeletal
1436448_a_at	Mm.2792.2	2.35	0.00404	0.50	Ptgs1	prostaglandin-endoperoxide synthase 1
1419206_at	Mm.3689.1	2.32	0.03132	0.68	Cd37	CD37 antigen
1435288_at	Mm.27317.3	2.25	0.02471	0.66	Coro1a	coronin, actin binding protein 1A
1448698_at	Mm.22288.1	2.24	0.01433	0.61	Ccnd1	cyclin D1
1448005_at	Mm.24385.1	2.22	0.02140	0.64	Sash1	SAM and SH3 domain containing 1
1420699_at	Mm.132943.1	2.18	0.03078	0.68	Clec7a	C-type lectin domain family 7, member a
1419480_at	Mm.1461.1	2.16	0.00812	0.55	Sell	selectin, lymphocyte
1418003_at	Mm.29811.1	2.15	0.00627	0.54	Rgcc	regulator of cell cycle
1418102_at	Mm.4451.1	2.13	0.00044	0.37	Hes1	hairy and enhancer of split 1 (Drosophila)
1424246_a_at	Mm.88645.1	2.12	0.02818	0.67	Tes	testis derived transcript
1452657_at	Mm.12390.1	2.11	0.02879	0.67	Ap1s2	adaptor-related protein complex 1, sigma 2 subunit
1450276_a_at	Mm.2416.1	2.1	0.00048	0.37	Scin	scinderin
1438511_a_at	Mm.29811.2	2.06	0.00485	0.52	Rgcc	regulator of cell cycle
1451344_at	Mm.41681.1	2.06	0.00770	0.55	Tmem119	transmembrane protein 119
1434800_at	Mm.39888.1	2.06	0.00367	0.50	Sv2b	synaptic vesicle glycoprotein 2 b
1448665_at	Mm.742.1	2.04	0.01010	0.58	Dmd	dystrophin, muscular dystrophy
1439426_x_at	Mm.45436.3	2.03	0.04229	0.71	Lyz1	lysozyme 1
1422542_at	Mm.89979.1	2.02	0.03721	0.69	Gpr34	G protein-coupled receptor 34
1418340_at	Mm.22673.1	2.01	0.00433	0.51	Fcer1g	Fc receptor, IgE, high affinity I, gamma polypeptide
1421400_at	Mm.6206.1	2.01	0.01064	0.59	Kcnmb1	potassium large conductance calcium-activated channel
1432686_at	Mm.159245.1	-2.04	0.00095	0.40	4833406M21Rik	RIKEN cDNA 4833406M21 gene
1417434_at	Mm.3711.1	-2.08	0.00010	0.26	Gpd2	glycerol phosphate dehydrogenase 2, mitochondrial
1436993_x_at	Mm.20399.2	-2.09	0.04682	0.71	Pfn2	profilin 2
1417585_at	Mm.28162.1	-2.1	0.00075	0.37	Nup210	nucleoporin 210
1450871_a_at	Mm.4606.1	-2.11	0.00780	0.55	Bcat1	branched chain aminotransferase 1, cytosolic
1441977_at	Mm.101520.2	-2.11	0.01557	0.62	9630023C09Rik	RIKEN cDNA 9630023C09 gene

1437856_at	Mm.12055.2	-2.12	0.00266	0.45	Ipmk	inositol polyphosphate multikinase
1450783_at	Mm.6718.1	-2.14	0.02235	0.65	Ifit1	interferon-induced protein with tetratricopeptide repeats
1437285_at	Mm.42110.2	-2.14	0.01198	0.59	Nadk2	NAD kinase 2, mitochondrial
1459311_at	Mm.210629.1	-2.14	0.00197	0.45	Pde4d	phosphodiesterase 4D, cAMP specific
1435774_at	Mm.46750.1	-2.15	0.03630	0.69	LOC106740	uncharacterized LOC106740; PHD finger protein 10
1439554_at	Mm.83648.1	-2.15	0.02097	0.64	Scmh1	sex comb on midleg homolog 1
1428323_at	Mm.41718.1	-2.17	0.00063	0.37	Gpd2	glycerol phosphate dehydrogenase 2, mitochondrial
1446974_at	Mm.217894.1	-2.18	0.03601	0.69	Pikfyve	phosphoinositide kinase, FYVE finger containing
1456200_at	Mm.12055.2	-2.2	0.00277	0.45	Ipmk	inositol polyphosphate multikinase
1440443_at	Mm.131090.1	-2.25	0.00589	0.53	E030016H06Rik	RIKEN cDNA E030016H06 gene
1457186_at	Mm.131416.1	-2.25	0.01702	0.63	Tbl1xr1	transducin (beta)-like 1X-linked receptor 1
1452608_at	Mm.220922.1	-2.26	0.00004	0.16	Mycbp	c-myc binding protein
1427940_s_at	Mm.220922.1	-2.27	0.00025	0.37	Mycbp	c-myc binding protein
1438957_x_at	Mm.196477.3	-2.27	0.00030	0.37	Cds2	CDP-diacylglycerol synthase
1416686_at	Mm.79983.1	-2.33	0.01648	0.63	Plod2	procollagen lysine, 2-oxoglutarate 5-dioxygenase 2
1437221_at	Mm.115282.1	-2.39	0.04472	0.71	Rrm2b	ribonucleotide reductase M2 B (TP53 inducible)
1453512_at	Mm.167748.1	-2.42	0.04457	0.71	5830407P18Rik	RIKEN cDNA 5830407P18 gene
1418210_at	Mm.20399.1	-2.49	0.00665	0.54	Pfn2	profilin 2
1451526_at	Mm.22834.1	-2.49	0.00293	0.46	Arhgap12	Rho GTPase activating protein 12
1430111_a_at	Mm.4606.3	-2.5	0.00666	0.54	Bcat1	branched chain aminotransferase 1, cytosolic
1423597_at	Mm.33083.1	-2.51	0.00707	0.54	Atp8a1	ATPase, aminophospholipid transporter (APLT), class I
1452741_s_at	Mm.41718.1	-2.63	0.03691	0.69	Gpd2	glycerol phosphate dehydrogenase 2, mitochondrial
1416687_at	Mm.79983.1	-2.82	0.02765	0.67	Plod2	procollagen lysine, 2-oxoglutarate 5-dioxygenase 2
1437287_at	Mm.42110.2	-2.87	0.00013	0.30	Nadk2	NAD kinase 2, mitochondrial
1418209_a_at	Mm.20399.1	-2.93	0.01096	0.59	Pfn2	profilin 2
1439036_a_at	Mm.4550.6	-2.97	0.03059	0.68	Atp1b1	ATPase, Na+/K+ transporting, beta 1 polypeptide
