# Supplementary material for: The miR-23a~27a~24-2 microRNA cluster buffers transcription and signaling pathways during hematopoiesis
Source: PLoS Genet. 2017 Jul 13;13(7):e1006887. doi: 10.1371/journal.pgen.1006887 (PMC5531666; doi:10.1371/journal.pgen.1006887)
Supplement: S2 Table — Two unique MiR-23a overexpressing 70Z/3 cell lines were generated through limiting dilution along with a control line infected with empty retrovirus. Cell lines were analyzed for genome wide RNA expression by microarray analysis using Affymetrix Mouse Genome 430 2.0 Arrays. Genes differentially regulated >2 fold between control and miR-23a overexpressing cell lines are shown. (PDF) [file pgen.1006887.s002.pdf]

Supplementary Table 2. Genes significantly changed in miR-23a overexpressing 70Z/3 Pre B Cells

Transcript Cluster ID	Transcript ID	Fold Change	ANOVA p-value	Gene Symbol	Description
1427409_at	Mm.39741.1	6.88	0.009117		membrane-associated ring finger (C3HC4) 9
1419611_at	Mm.37735.1	-2.17	0.016543	4632415L05Rik	RRS1 ribosome biogenesis regulator homolog pseudogene
1433082_at	Mm.159285.1	3.11	0.013689	4930448K20Rik	glyceraldehyde-3-phosphate dehydrogenase pseudogene
1447937_a_at	Mm.41016.1	5.73	0.017454	4933409K07Rik	RIKEN cDNA 4933409K07 gene; predicted gene 10590; predicted gene, 21093
1435098_at	Mm.55513.1	5.11	0.023283	AA474331	expressed sequence AA474331
1420025_s_at	Mm.221003.1	-2.37	0.007042	Aak1	AP2 associated kinase 1
1423685_at	Mm.24174.1	-2.03	0.008902	Aars	alanyl-tRNA synthetase
1434474_at	Mm.41942.1	5.17	0.036011	Abca5	ATP-binding cassette, sub-family A (ABC1), member 5
1452233_at	Mm.196634.1	-8.15	0.034669	Abcc1	ATP-binding cassette, sub-family C (CFTR/MRP), member 1
1443046_at	Mm.194462.1	-4.8	0.045874	Abcd3	ATP-binding cassette, sub-family D (ALD), member 3
1428276_at	Mm.142570.1	-2.14	0.023571	Abhd13	abhydrolase domain containing 13
1417946_at	Mm.7100.1	12.37	0.000125	Abhd3	abhydrolase domain containing 3
1445754_at	Mm.173067.1	3.3	0.029888	Acacb	acetyl-Coenzyme A carboxylase beta
1452138_a_at	Mm.13451.2	-2.62	0.047715	Ace2	angiotensin I converting enzyme (peptidyl-dipeptidase A) 2
1446315_at	Mm.208396.1	-3.17	0.020521	Acly	ATP citrate lyase
1452415_at	Mm.214752.1	4.97	0.047279	Actn1	actinin, alpha 1
1435293_at	Mm.40034.1	-7.23	0.007239	Adam22	a disintegrin and metallopeptidase domain 22
1452495_at	Mm.157910.2	-4.36	0.005592	Adam28	a disintegrin and metallopeptidase domain 28
1444628_at	Mm.108550.1	-7.64	0.023432	Adam33	a disintegrin and metallopeptidase domain 33
1428030_at	Mm.55652.1	-7.71	0.001062	Adam34	a disintegrin and metallopeptidase domain 34
1419476_at	Mm.36742.1	4.45	0.009971	Adamdec1	ADAM-like, decysin 1
1420336_at	Mm.175389.1	-4.39	0.009188	Adamtsl5	ADAMTS-like 5
1429022_at	Mm.41600.1	-4.72	0.004701	Adcyap1r1	adenylate cyclase activating polypeptide 1 receptor 1
1451914_a_at	Mm.104155.2	-2.53	0.046772	Add2	adducin 2 (beta)
1429608_at	Mm.46265.1	2.48	0.031931	Adh6a	alcohol dehydrogenase 6A (class V)
1424729_at	Mm.28800.1	-15.3	0.000373	Adig	adipogenin
1460183_at	Mm.20047.1	-2.4	0.041541	Adprh	ADP-ribosylarginine hydrolase
1427440_a_at	Mm.29117.1	-8.31	0.001373	Afm	afamin
1440914_s_at	Mm.202171.1	-2.23	0.007697	Aftph	aftiphilin
1422841_at	Mm.200898.1	-4.52	0.001142	Agpat6	1-acylglycerol-3-phosphate O-acyltransferase 6 (lysophosphatidic acid
1432385_a_at	Mm.45991.2	-2.42	0.027451	Agtpbp1	ATP/GTP binding protein 1
1452217_at	Mm.203866.1	2.58	0.008122	Ahnak	AHNAK nucleoprotein (desmoyokin)
1439796_at	Mm.35543.1	-5.27	0.022227	AI747448	expressed sequence AI747448
1445173_at	Mm.150204.1	-5.35	0.046119	AK129341	cDNA sequence AK129341
1419136_at	Mm.41337.1	-3.58	0.005065	Akr1c18	aldo-keto reductase family 1, member C18
1437736_at	Mm.148007.2	-3.56	0.02795	Akt1s1	AKT1 substrate 1 (proline-rich)
1430219_at	Mm.88777.3	13.36	0.022915	Aktip	thymoma viral proto-oncogene 1 interacting protein
1459337_at	Mm.209992.1	-6.78	0.000419	Alg6	asparagine-linked glycosylation 6 (alpha-1,3,-glucosyltransferase)
1422699_at	Mm.12286.1	-7.95	0.014226	Alox12	arachidonate 12-lipoxygenase
1441962_at	Mm.41072.1	-2.47	0.016349	Alox5	arachidonate 5-lipoxygenase
1449417_at	Mm.8437.1	-3.36	0.008967	Ambn	ameloblastin
1444117_at	Mm.44915.1	-3.74	0.026511	Amigo1	adhesion molecule with Ig like domain 1
1452387_a_at	Mm.21145.2	-2.55	0.044202	Amotl2	angiomotin-like 2
1441713_at	Mm.37341.1	-5.11	0.016818	Anapc7	anaphase promoting complex subunit 7
1459317_at	Mm.208496.1	-3.46	0.02372	Ank2	ankyrin 2, brain
1434265_s_at	Mm.41182.1	-5.35	0.046227	Ank2	ankyrin 2, brain
1443654_at	Mm.212632.1	-2.26	0.02681	Ankfy1	ankyrin repeat and FYVE domain containing 1
1459952_at	Mm.42670.1	-2.07	0.032747	Ankrd17	ankyrin repeat domain 17
1429300_at	Mm.35977.1	-8.32	0.038057	Ankrd9	ankyrin repeat domain 9
1453042_at	Mm.23165.1	4.96	0.015141	Anks4b	ankyrin repeat and sterile alpha motif domain containing 4B
1452657_at	Mm.12390.1	2.08	0.013004	Ap1s2	adaptor-related protein complex 1, sigma 2 subunit
1424739_at	Mm.87037.1	-7.61	0.007938	Ap5m1	adaptor-related protein complex 5, mu 1 subunit
1450497_at	Mm.57247.1	4.61	0.045064	Apc2	adenomatosis polyposis coli 2
1419059_at	Mm.2165.1	3.02	0.032539	Apcs	serum amyloid P-component
1419232_a_at	Mm.26743.1	10.52	0.006373	Apoa1	apolipoprotein A-I
1419233_x_at	Mm.26743.1	-2.24	0.016221	Apoa1	apolipoprotein A-I
1418278_at	Mm.178973.1	-2.12	0.009096	Apoc3	apolipoprotein C-III
1420621_a_at	Mm.15571.1	2.49	0.018655	App	amyloid beta (A4) precursor protein
1434464_at	Mm.46277.1	6.38	0.0372	Aqp12	aquaporin 12
1421605_a_at	Mm.22477.1	-10.7	0.000614	Aqp9	aquaporin 9
1434074_x_at	Mm.1486.2	-9.65	0.03371	Arf4	ADP-ribosylation factor 4
1451526_at	Mm.22834.1	-2.42	0.001113	Arhgap12	Rho GTPase activating protein 12
1429918_at	Mm.93461.1	-6.74	0.01034	Arhgap20	Rho GTPase activating protein 20
1440688_at	Mm.183885.1	-2.28	0.02731	Arhgap26	Rho GTPase activating protein 26
1420973_at	Mm.46544.1	-2.4	0.027452	Arid5b	AT rich interactive domain 5B (MRF1-like)
1458238_at	Mm.213295.1	-9.64	0.022382	Arid5b	AT rich interactive domain 5B (MRF1-like)
1431945_at	Mm.159203.1	-16.12	0.000966	Arl13a	ADP-ribosylation factor-like 13A
1441175_at	Mm.212864.1	-3.3	0.015516	Arx	aristaless related homeobox
1426116_at	Mm.215164.1	3.7	0.021659	Asb18	ankyrin repeat and SOCS box-containing 18
1452958_at	Mm.151745.1	-5.34	0.000209	Asphd2	aspartate beta-hydroxylase domain containing 2
1458366_at	Mm.35769.1	-3.25	0.003418	Asxl2	additional sex combs like 2 (Drosophila)
1443146_at	Mm.214676.1	-5.3	0.005627	Atad2	ATPase family, AAA domain containing 2
1452979_at	Mm.82354.1	4.76	0.028904	Atat1	alpha tubulin acetyltransferase 1
1439457_x_at	Mm.9852.4	-2.4	0.026631	Atg12	autophagy related 12
1439036_a_at	Mm.4550.6	-4.07	0.039663	Atp1b1	ATPase, Na+/K+ transporting, beta 1 polypeptide
1434798_at	Mm.19298.1	-3.1	0.02089	Atp6v0d2	ATPase, H+ transporting, lysosomal V0 subunit D2
1423597_at	Mm.33083.1	-2.45	0.016541	Atp8a1	ATPase, aminophospholipid transporter (APLT), class I, type 8A, member 1
1431953_at	Mm.220136.1	-4.23	0.037645	Atp8a2	ATPase, aminophospholipid transporter-like, class I, type 8A, member 2
1425838_at	Mm.10288.2	-2.66	0.043585	Atp9a	ATPase, class II, type 9A
1427604_a_at	Mm.10288.3	-13.64	0.013404	Atp9a	ATPase, class II, type 9A
1442486_at	Mm.35025.1	11.25	0.049229	AU015336	expressed sequence AU015336
1444674_at	Mm.130323.2	-8.67	0.009439	AU015680	expressed sequence AU015680
1446901_at	Mm.173498.1	5.18	0.018881	AU022077	expressed sequence AU022077
1446403_at	Mm.173577.1	4.68	0.031756	AU022899	expressed sequence AU022899
1418603_at	Mm.4351.1	-11.95	0.00383	Avpr1a	arginine vasopressin receptor 1A
1441664_at	Mm.44379.1	2.84	0.046577	AW049021	expressed sequence AW049021
1440993_at	Mm.36341.1	5.31	0.000007	AW111846	expressed sequence AW111846
1445526_at	Mm.26887.1	7.66	0.005915	AY512915	cDNA sequence AY512915
1440124_at	Mm.117810.1	9.28	0.022747	B230334C09Rik	RIKEN cDNA B230334C09 gene
1459801_at	Mm.121375.1	-3.18	0.009806	B3galt5	UDP-Gal:betaGlcNAc beta 1,3-galactosyltransferase, polypeptide 5
1435913_at	Mm.28436.1	-2.02	0.002606	B4galnt4	beta-1,4-N-acetyl-galactosaminyl transferase 4
1435957_at	Mm.59156.1	-2.06	0.003721	B830032F12	uncharacterized protein B830032F12
1445714_at	Mm.125803.1	-11.48	0.000289	B930042K01Rik	RIKEN cDNA B930042K01 gene
1455363_at	Mm.43133.1	-2.54	0.043566	Bai1	brain-specific angiogenesis inhibitor 1
1455716_at	Mm.65350.1	-9.11	0.023624	BB187676	expressed sequence BB187676
1435598_at	Mm.129506.1	-5.33	0.011772	BB319198	expressed sequence BB319198
1426936_at	Mm.220992.1	2.31	0.039403	BC005512	cDNA sequence BC005512; RIKEN cDNA F630007L15 gene; predicted gene 695
1454761_at	Mm.29678.1	-4.76	0.024377	BC005764	cDNA sequence BC005764
1425807_at	Mm.216458.1	-2.41	0.039453	BC021891	cDNA sequence BC021891

1427662_at	Mm.218881.1	-2.43	0.014731	BC025933	cDNA sequence BC025933
1435175_at	Mm.29270.1	-3.35	0.022299	BC034090	cDNA sequence BC034090
1445849_at	Mm.26025.1	9.82	0.016233	BC080696	cDNA sequence BC080696
1451681_at	Mm.214923.1	-3.28	0.048923	BC089597	cDNA sequence BC089597
1458557_at	Mm.161857.1	6.96	0.003163	Bcas3	breast carcinoma amplified sequence 3
1450871_a_at	Mm.4606.1	-2.01	0.018319	Bcat1	branched chain aminotransferase 1, cytosolic
1430111_a_at	Mm.4606.3	-3	0.004778	Bcat1	branched chain aminotransferase 1, cytosolic
1450812_at	Mm.193421.1	-6.71	0.001739	Bcl2a1a	B cell leukemia/lymphoma 2 related protein A1a
1422169_a_at	Mm.1442.1	-5.52	0.015776	Bdnf	brain derived neurotrophic factor
1435351_at	Mm.45533.1	2.88	0.025552	Bend5	BEN domain containing 5
1417388_at	Mm.94160.1	5.29	0.002518	Bex2	brain expressed X-linked 2
1418271_at	Mm.143811.1	-12.74	0.007205	Bhlhe22	basic helix-loop-helix family, member e22
1421099_at	Mm.89873.1	-4.79	0.007353	Bhlhe41	basic helix-loop-helix family, member e41
1419616_at	Mm.7106.1	-4.15	0.016991	Bmpr2	bone morphogenetic protein receptor, type II (serine/threonine kinase)
1422084_at	Mm.504.1	-5.37	0.000394	Bmx	BMX non-receptor tyrosine kinase
1427516_a_at	Mm.41561.2	-5.66	0.00208	Boc	biregional cell adhesion molecule-related/down-regulated by oncogenes(Cdon)
1420347_at	Mm.28438.1	4.85	0.043121	Bpifa1	BPI fold containing family A, member 1
1419348_at	Mm.4539.1	-4.39	0.037342	Bpifa2	BPI fold containing family A, member 2
1456525_at	Mm.38123.2	-2.55	0.000162	Brat1	BRCA1-associated ATM activator 1
1449453_at	Mm.24612.1	-10.44	0.001732	Bst1	bone marrow stromal cell antigen 1
1443142_at	Mm.119717.1	-2.34	0.014353	Btrc	beta-transducin repeat containing protein
1454517_at	Mm.159894.1	-5.93	0.005703	C030011I16Rik	RIKEN cDNA C030011I16 gene
1435894_at	Mm.54201.1	16.79	0.02778	C030014L02	uncharacterized protein C030014L02
1456828_at	Mm.39935.1	-7.74	0.012042	C030023E24Rik	RIKEN cDNA C030023E24 gene
1441489_at	Mm.102655.1	3.53	0.004394	C130023C23Rik	Riken cDNA C130023C23 gene
1453791_at	Mm.53761.1	-6.28	0.00133	C130071C03Rik	RIKEN cDNA C130071C03 gene
1441632_at	Mm.88458.1	-3.33	0.021742	C130079B09Rik	RIKEN cDNA C130079B09 gene
1416887_at	Mm.2485.1	2.21	0.036026	C1d	C1D nuclear receptor co-repressor
1425175_at	Mm.81159.1	-3.87	0.024366	C1ql3	C1q-like 3
1451620_at	Mm.81159.1	-7.74	0.025773	C1ql3	C1q-like 3
1441912_x_at	Mm.73650.1	-2.09	0.02236	C2	complement component 2 (within H-2S)
1446236_at	Mm.215887.1	-5.53	0.03145	C230072F16Rik	RIKEN cDNA C230072F16 gene
1440842_at	Mm.38162.1	-7.99	0.006547	C230085N15Rik	RIKEN cDNA C230085N15 gene
1438125_at	Mm.111724.1	-8.08	0.000742	C230085N15Rik	RIKEN cDNA C230085N15 gene
1458487_at	Mm.74627.2	2.45	0.042828	C230096K16Rik	RIKEN cDNA C230096K16 gene; Kruppel-like factor 3 (basic)
1442082_at	Mm.118523.1	-8.9	0.028915	C3ar1	complement component 3a receptor 1
1439171_at	Mm.101504.1	5.66	0.000316	C530008M17Rik	RIKEN cDNA C530008M17 gene
1444370_at	Mm.173815.1	-7.99	0.002777	C77058	expressed sequence C77058
1442937_at	Mm.172622.1	-8.61	0.009377	C77190	expressed sequence C77190
1442500_at	Mm.172632.1	-5.62	0.049651	C77406	expressed sequence C77406
1446575_at	Mm.172646.1	2.69	0.028028	C77583	expressed sequence C77583
1445490_at	Mm.166448.1	-2.48	0.007859	C77805	expressed sequence C77805
1458862_at	Mm.5740.1	-2.59	0.026067	C78704	expressed sequence C78704
1445156_at	Mm.172124.1	-3.74	0.012475	C81001	expressed sequence C81001
1449689_at	Mm.6062.1	-5.69	0.019797	C81600	expressed sequence C81600
1445810_at	Mm.172961.1	-7.09	0.049456	C85163	expressed sequence C85163
1446033_at	Mm.25515.1	-4.91	0.041814	C87882	expressed sequence C87882
1436602_x_at	Mm.91991.1	2.78	0.020602	Cacna1b	calcium channel, voltage-dependent, N type, alpha 1B subunit
1419225_at	Mm.42133.1	-4.18	0.030801	Cacna2d3	calcium channel, voltage-dependent, alpha2/delta subunit 3
1459579_at	Mm.59119.1	8.85	0.008835	Cacng8	calcium channel, voltage-dependent, gamma subunit 8
1435146_s_at	Mm.129786.1	-7.7	0.006388	Cadm2	cell adhesion molecule 2
1451499_at	Mm.41732.1	2.82	0.025224	Cadps2	Ca2+-dependent activator protein for secretion 2
1458836_at	Mm.182847.1	-5.93	0.023492	Calb1	calbindin 1
1427355_at	Mm.4361.2	-14.4	0.00886	Calca	calcitonin/calcitonin-related polypeptide, alpha
1437125_at	Mm.102244.1	-2.87	0.034436	Camk2a	calcium/calmodulin-dependent protein kinase II alpha
1444031_at	Mm.208056.1	-2.36	0.031963	Camk2d	calcium/calmodulin-dependent protein kinase II, delta
1440455_at	Mm.22965.1	-3.35	0.015992	Camk2n1	calcium/calmodulin-dependent protein kinase II inhibitor 1
1439843_at	Mm.45058.1	-4.23	0.036401	Camk4	calcium/calmodulin-dependent protein kinase IV
1456930_at	Mm.100960.1	-2.27	0.001146	Camsap1	calmodulin regulated spectrin-associated protein 1
1441845_at	Mm.84450.1	-3.05	0.02969	Caps2	calcyphosphine 2
1447928_at	Mm.77753.1	4.01	0.011587	Car5b	carbonic anhydrase 5b, mitochondrial
1443787_x_at	Mm.183131.1	-2.76	0.002953	Casp14	caspase 14
1438705_at	Mm.12429.2	21.8	0.029296	Cbfa2t3	core-binding factor, runt domain, alpha subunit 2, translocated to, 3 (human)
1453918_at	Mm.85005.1	-4.71	0.04556	Ccbe1	collagen and calcium binding EGF domains 1
1456101_at	Mm.28555.2	17.03	0.004934	Ccdc175	coiled-coil domain containing 175
1453330_at	Mm.45291.1	-2.41	0.047611	Ccdc88c	coiled-coil domain containing 88C
1429635_at	Mm.87424.1	-2.13	0.024477	Ccdc89	coiled-coil domain containing 89
1448898_at	Mm.2271.1	6.68	0.000217	Ccl9	chemokine (C-C motif) ligand 9
1417936_at	Mm.2271.1	4.97	0.000959	Ccl9	chemokine (C-C motif) ligand 9
1448698_at	Mm.22288.1	2.63	0.011428	Ccnd1	cyclin D1
1430645_at	Mm.103181.1	5.59	0.007408	Cd200r3	CD200 receptor 3
1427994_at	Mm.22903.1	2.22	0.010431	Cd300lf	CD300 antigen like family member F
1419206_at	Mm.3689.1	2.33	0.032116	Cd37	CD37 antigen
1451950_a_at	Mm.89474.5	3.74	0.014358	Cd80	CD80 antigen
1432826_a_at	Mm.89474.8	3.22	0.031732	Cd80	CD80 antigen
1441908_x_at	Mm.138523.1	-2.99	0.012138	Cd93	CD93 antigen
1442667_at	Mm.156928.1	-7.34	0.008647	Cdc40	cell division cycle 40
1425092_at	Mm.40247.1	-4.73	0.023603	Cdh10	cadherin 10
1419331_at	Mm.33402.1	4.67	0.004424	Cdh17	cadherin 17
1449637_at	Mm.22789.1	6.28	0.006679	Cdh4	cadherin 4
1447767_at	Mm.165569.1	-2.15	0.044132	Cdkl4	cyclin-dependent kinase-like 4
1434256_s_at	Mm.196477.2	-2.2	0.004059	Cds2	CDP-diacylglycerol synthase (phosphatidate cytidylyltransferase) 2
1438957_x_at	Mm.196477.3	-2.21	0.000282	Cds2	CDP-diacylglycerol synthase (phosphatidate cytidylyltransferase) 2
1427712_at	Mm.14114.6	-3.09	0.034004	Ceacam1	carcinoembryonic antigen-related cell adhesion molecule 1
1448281_a_at	Mm.21925.1	-11.78	0.048095	Cela2a	chymotrypsin-like elastase family, member 2A
1452240_at	Mm.29205.1	12.01	0.003645	Celf4	CUGBP, Elav-like family member 4
1425067_at	Mm.39945.1	-4.34	0.015592	Celsr3	cadherin, EGF LAG seven-pass G-type receptor 3 (flamingo homolog, Drosophil
1424266_s_at	Mm.29110.1	-7.76	0.00542	Ces1f	carboxylesterase 1F
1452279_at	Mm.8308.1	10.33	0.004286	Cfp	complement factor properdin
1424529_s_at	Mm.45127.1	2.1	0.019637	Cgref1	cell growth regulator with EF hand domain 1
1418149_at	Mm.4137.1	-2.69	0.01122	Chga	chromogranin A
1459584_at	Mm.212781.1	-4.79	0.025455	Chmp3	charged multivesicular body protein 3
1428574_a_at	Mm.34715.2	8.23	0.010163	Chn2	chimerin (chimaerin) 2
1450299_at	Mm.4611.1	3.87	0.017697	Chrna7	cholinergic receptor, nicotinic, alpha polypeptide 7
1457008_at	Mm.32694.1	3.95	0.023427	Chrnb4	cholinergic receptor, nicotinic, beta polypeptide 4
1420560_at	Mm.4980.1	-6.38	0.001576	Chrne	cholinergic receptor, nicotinic, epsilon polypeptide
1422758_at	Mm.12968.1	-3.61	0.044962	Chst2	carbohydrate sulfotransferase 2
1437271_at	Mm.32518.2	2.06	0.011657	Clcf1	cardiotrophin-like cytokine factor 1
1427591_at	Mm.22885.1	-3.03	0.04365	Clcn1	chloride channel 1
1434651_a_at	Mm.28921.5	-3.44	0.046432	Cldn3	claudin 3

1421182_at	Mm.30700.1	2.93	0.041192	Clec1b	C-type lectin domain family 1, member b
1424673_at	Mm.197689.1	4.57	0.048225	Clec2h	C-type lectin domain family 2, member h
1422166_at	Mm.197579.1	-3.99	0.041418	Clec2i	C-type lectin domain family 2, member i
1427428_at	Mm.109183.1	-10.26	0.015619	Clec4g	C-type lectin domain family 4, member g
1420699_at	Mm.132943.1	2.3	0.029644	Clec7a	C-type lectin domain family 7, member a
1430240_a_at	Mm.27026.2	2.85	0.011405	Clgn	calmegin
1457829_at	Mm.101027.1	-5.65	0.03128	Clgn	calmegin
1454866_s_at	Mm.44747.1	-8.4	0.01479	Clic6	chloride intracellular channel 6
1435504_at	Mm.141984.1	-5.56	0.009136	Clip4	CAP-GLY domain containing linker protein family, member 4
1443170_at	Mm.103106.1	-6.13	0.001287	Cnnm1	cyclin M1
1425476_at	Mm.155579.1	-3.87	0.029964	Col4a5	collagen, type IV, alpha 5
1452250_a_at	Mm.1949.1	-3.2	0.044335	Col6a2	collagen, type VI, alpha 2
1448514_at	Mm.180182.1	4.34	0.000997	Cox5b	cytochrome c oxidase subunit Vb
1449931_at	Mm.23178.1	-9.99	0.005141	Cpeb4	cytoplasmic polyadenylation element binding protein 4
1421583_at	Mm.1376.1	6.34	0.000797	Creb1	cAMP responsive element binding protein 1
1431468_at	Mm.99740.1	4.63	0.016643	Crisp4	cysteine-rich secretory protein 4
1418306_at	Mm.29488.1	2.35	0.032445	Crybb1	crystallin, beta B1
1419872_at	Mm.201971.1	4.31	0.000329	Csf1r	colony stimulating factor 1 receptor
1455579_at	Mm.4908.3	9.13	0.021419	Csn1s2a	casein alpha s2-like A
1418221_at	Mm.4886.1	-5.93	0.014225	Csn1s2b	casein alpha s2-like B
1416811_s_at	Mm.30144.1	2.05	0.015192	Ctla2a	cytotoxic T lymphocyte-associated protein 2 alpha; cytotoxic T lymphocyte-
1437275_at	Mm.18962.2	6.95	0.015525	Ctnna1	catenin (cadherin associated protein), alpha 1
1453996_a_at	Mm.46079.2	7.55	0.016129	Cts3	cathepsin 3
1446266_at	Mm.215909.1	-3.53	0.013997	Cts8	cathepsin 8
1451019_at	Mm.29561.1	-2.55	0.019145	Ctsf	cathepsin F
1421314_at	Mm.206790.1	-5.36	0.002183	Cttn	cortactin
1451610_at	Mm.10545.1	-7.14	0.010748	Cxcl17	chemokine (C-X-C motif) ligand 17
1431021_at	Mm.53836.1	-2.75	0.030584	Cyb561d1	cytochrome b-561 domain containing 1
1422185_a_at	Mm.22560.1	2.18	0.003191	Cyb5r3	cytochrome b5 reductase 3
1450752_at	Mm.4872.1	-7.55	0.031473	Cyct	cytochrome c, testis
1457134_at	Mm.151188.1	5.2	0.006971	Cyfip1	cytoplasmic FMR1 interacting protein 1
1423630_at	Mm.34598.1	2.52	0.019723	Cygb	cytoglobin
1448804_at	Mm.108678.1	3.03	0.010028	Cyp11a1	cytochrome P450, family 11, subfamily a, polypeptide 1
1450574_at	Mm.215158.1	-2.31	0.01386	Cyp11b2	cytochrome P450, family 11, subfamily b, polypeptide 2
1432081_at	Mm.4443.2	-3.77	0.001431	Cyp1b1	cytochrome P450, family 1, subfamily b, polypeptide 1
1422230_s_at	Mm.154643.1	-6.96	0.00063	Cyp2a4	cytochrome P450, family 2, subfamily a, polypeptide 4; cytochrome P450
1429994_s_at	Mm.34966.1	-5.4	0.003039	Cyp2c65	cytochrome P450, family 2, subfamily c, polypeptide 65
1421741_at	Mm.30303.1	6.21	0.034356	Cyp3a16	cytochrome P450, family 3, subfamily a, polypeptide 16
1423257_at	Mm.7459.1	-3.59	0.038202	Cyp4a14	cytochrome P450, family 4, subfamily a, polypeptide 14
1419219_at	Mm.137441.1	4.44	0.001195	Cyp4f18	cytochrome P450, family 4, subfamily f, polypeptide 18
1422100_at	Mm.57029.1	-7.88	0.014506	Cyp7a1	cytochrome P450, family 7, subfamily a, polypeptide 1
1421074_at	Mm.4781.1	-9.47	0.00307	Cyp7b1	cytochrome P450, family 7, subfamily b, polypeptide 1
1459475_at	Mm.155229.1	5.63	0.000365	D10Ertd761e	DNA segment, Chr 10, ERATO Doi 761, expressed
1457304_at	Mm.26254.1	-2.49	0.047937	D13Ertd787e	DNA segment, Chr 13, ERATO Doi 787, expressed
1446118_at	Mm.154700.1	-2.6	0.023794	D17Ertd165e	DNA segment, Chr 17, ERATO Doi 165, expressed
1447984_at	Mm.34935.1	-2.21	0.045838	D1Ertd75e	DNA segment, Chr 1, ERATO Doi 75, expressed
1441672_at	Mm.17084.1	5.37	0.002418	D3Ertd547e	DNA segment, Chr 3, ERATO Doi 547, expressed
1446856_at	Mm.24493.1	-10.97	0.028863	D3Wsu167e	DNA segment, Chr 3, Wayne State University 167, expressed
1454249_at	Mm.160117.1	-9.23	0.044365	D530049N12Rik	RIKEN cDNA D530049N12 gene
1458531_at	Mm.155057.1	-3.92	0.004812	D6Ertd588e	DNA segment, Chr 6, ERATO Doi 588, expressed
1458040_at	Mm.33177.1	3.56	0.010207	D7Wsu130e	DNA segment, Chr 7, Wayne State University 130, expressed
1459932_at	Mm.219138.1	-7.7	0.003593	D8Ertd28e	DNA segment, Chr 8, ERATO Doi 28, expressed
1456998_at	Mm.26365.1	-2.01	0.043251	Dbpht2	DNA binding protein with his-thr domain
1451289_at	Mm.44752.2	4.06	0.01403	Dclk1	doublecortin-like kinase 1
1458596_at	Mm.35262.1	-3.66	0.024763	Dctn4	dynactin 4
1429299_at	Mm.30203.1	5.38	0.000408	Ddah1	dimethylarginine dimethylaminohydrolase 1
1427289_at	Mm.24760.1	-5.31	0.024819	Ddhd1	DDHD domain containing 1
1436268_at	Mm.157859.1	-7.64	0.032579	Ddn	dendrin
1422738_at	Mm.4999.1	-6.71	0.000954	Ddr2	discoidin domain receptor family, member 2
1456076_at	Mm.82820.1	2.72	0.015743	Defb19	defensin beta 19
1427612_at	Mm.171224.1	7.13	0.00127	Defb9	defensin beta 9
1426732_at	Mm.6712.1	-7.75	0.003594	Des	desmin
1439986_at	Mm.139159.1	-8.83	0.000484	Dgki	diacylglycerol kinase, iota
1420768_a_at	Mm.170797.1	-8.06	0.001937	Dhx58	DEXH (Asp-Glu-X-His) box polypeptide 58
1420595_at	Mm.5165.1	-8.47	0.048812	Dlx4	distal-less homeobox 4
1439376_x_at	Mm.22480.4	-3.56	0.001386	Dmtf1	cyclin D binding myb-like transcription factor 1
1437094_x_at	Mm.79127.1	-3.28	0.001417	Dnaic1	dynein, axonemal, intermediate chain 1
1421961_a_at	Mm.20437.1	-3.99	0.033723	Dnajb5	DnaJ (Hsp40) homolog, subfamily B, member 5
1447649_x_at	Mm.70400.1	-3.2	0.049559	Dnajc1	DnaJ (Hsp40) homolog, subfamily C, member 1
1423671_at	Mm.39067.1	-4.02	0.035175	Dner	delta/notch-like EGF-related receptor
1445232_at	Mm.174042.1	-2.06	0.017055	Dock9	dedicator of cytokinesis 9
1442855_at	Mm.210850.1	-6.75	0.023353	Dopey1	dopey family member 1
1451394_at	Mm.42078.1	-12.21	0.042034	Dpp6	dipeptidylpeptidase 6
1458000_at	Mm.107228.1	-3.38	0.012261	Dsg1a	desmoglein 1 alpha
1435494_s_at	Mm.203911.1	-7.43	0.001667	Dsp	desmoplakin
1421117_at	Mm.25326.1	3.5	0.038497	Dst	dystonin
1447677_x_at	Mm.166571.1	6.61	0.017951	Dtd1	D-tyrosyl-tRNA deacylase 1
1425822_a_at	Mm.1645.2	-4.03	0.007112	Dtx1	deltex 1 homolog (Drosophila)
1426189_at	Mm.219646.1	-7.27	0.043714	Dusp15	dual specificity phosphatase-like 15
1440443_at	Mm.131090.1	-2.29	0.012966	E030016H06Rik	RIKEN cDNA E030016H06 gene
1441143_at	Mm.39381.1	-2.87	0.045817	E130309D14Rik	RIKEN cDNA E130309D14 gene
1448039_at	Mm.26748.2	-4.5	0.031541	E430024I08Rik	RIKEN cDNA E430024I08 gene
1422412_x_at	Mm.195444.1	12.41	0.000143	Ear3	eosinophil-associated, ribonuclease A family, member 3
1457441_at	Mm.192344.1	-2.73	0.015623	Ebf1	early B cell factor 1
1451924_a_at	Mm.14543.2	-3.94	0.026453	Edn1	endothelin 1
1438696_at	Mm.9478.2	-6.67	0.034185	Edn3	endothelin 3
1447668_x_at	Mm.148079.1	-4.45	0.001392	Efemp2	epidermal growth factor-containing fibulin-like extracellular matrix protein 2
1419474_a_at	Mm.10724.1	-2.33	0.044016	Ehf	ets homologous factor
1434323_at	Mm.183083.2	4.31	0.024621	Eif3h	eukaryotic translation initiation factor 3, subunit H
1438686_at	Mm.3428.3	-2.08	0.015154	Eif4g1	eukaryotic translation initiation factor 4, gamma 1
1455484_at	Mm.3477.3	-9.91	0.001507	Elavl3	ELAV (embryonic lethal, abnormal vision, Drosophila)-like 3 (Hu antigen C)
1451392_at	Mm.24440.1	2.25	0.04938	Elmod3	ELMO/CED-12 domain containing 3
1441091_at	Mm.212628.1	-8.83	0.02279	Elovl7	ELOVL family member 7, elongation of long chain fatty acids (yeast)
1448649_at	Mm.1193.1	-2.01	0.003412	Enpep	glutamyl aminopeptidase
1427302_at	Mm.53371.1	-5	0.003192	Enpp3	ectonucleotide pyrophosphatase/phosphodiesterase 3
1428088_at	Mm.23672.1	3.47	0.01253	Enthd2	ENTH domain containing 2
1452565_x_at	Mm.1232.4	2.41	0.001371	Env	uncharacterized LOC641050
1457758_at	Mm.117577.1	-4.18	0.003035	Eny2	enhancer of yellow 2 homolog (Drosophila)
1449888_at	Mm.1415.1	2.8	0.032224	Epas1	endothelial PAS domain protein 1
1418051_at	Mm.1480.1	2.04	0.03699	Ephb6	Eph receptor B6

1449136_at	Mm.1315.1	-7.19	0.006309	Epx	eosinophil peroxidase
1456511_x_at	Mm.4881.2	-14.08	0.003722	Eras	ES cell-expressed Ras
1429439_at	Mm.46510.2	-7.69	0.038411	Ercc8	excision repaiross-complementing rodent repair deficiency, complementation
1419816_s_at	Mm.200246.2	-3.06	0.046783	Errfi1	ERBB receptor feedback inhibitor 1
1433683_at	Mm.3107.3	-2.22	0.007071	Esrp2	epithelial splicing regulatory protein 2
1441921_x_at	Mm.127529.1	-2.71	0.044695	Esrrb	estrogen related receptor, beta
1440064_at	Mm.169632.1	10.56	0.021111	Etl4	enhancer trap locus 4
1416268_at	Mm.22365.1	2.6	0.027936	Ets2	E26 avian leukemia oncogene 2, 3' domain
1418993_s_at	Mm.2578.1	3.78	0.001238	F10	coagulation factor X
1447530_at	Mm.165807.1	-10.57	0.036153	F8a	factor 8-associated gene A
1448764_a_at	Mm.22126.1	3.67	0.049816	Fabp1	fatty acid binding protein 1, liver
1434203_at	Mm.40773.1	-3.25	0.019751	Fam107a	family with sequence similarity 107, member A
1446891_at	Mm.173737.1	-7.25	0.001273	Fam117b	family with sequence similarity 117, member B
1453955_a_at	Mm.84527.1	2.8	0.026316	Fam122c	family with sequence similarity 122, member C
1454942_at	Mm.23983.1	5.33	0.000557	Fam129a	family with sequence similarity 129, member A
1457076_at	Mm.82549.1	-4.19	0.016658	Fam131c	family with sequence similarity 131, member C
1443569_at	Mm.131739.1	-2.47	0.000003	Fam161a	family with sequence similarity 161, member A
1419171_at	Mm.68155.1	-2.4	0.035578	Fam174a	family with sequence similarity 174, member A
1453510_s_at	Mm.159618.1	3.39	0.001077	Fam184a	family with sequence similarity 184, member A
1438987_at	Mm.56514.1	-3.74	0.001279	Fam71d	family with sequence similarity 71, member D
1426996_at	Mm.24508.1	6.89	0.01667	Fam83h	family with sequence similarity 83, member H
1424583_at	Mm.192479.1	-4.36	0.033621	Farp2	FERM, RhoGEF and pleckstrin domain protein 2
1458413_at	Mm.131256.1	-3.35	0.011529	Fbxw8	F-box and WD-40 domain protein 8
1425062_at	Mm.123525.1	2.25	0.048033	Fcrl1	Fc receptor-like 1
1438919_x_at	Mm.3204.4	-2.55	0.036251	Fdft1	farnesyl diphosphate farnesyl transferase 1
1430012_at	Mm.158223.1	-2.02	0.009941	Fendrr	Foxf1 adjacent non-coding developmental regulatory RNA
1437369_at	Mm.8142.2	-4.89	0.032131	Fgd1	FYVE, RhoGEF and PH domain containing 1
1454593_at	Mm.159673.1	-2.87	0.043769	Fgd3	FYVE, RhoGEF and PH domain containing 3
1420690_at	Mm.25003.1	-4.08	0.002814	Fgf10	fibroblast growth factor 10
1440270_at	Mm.51802.1	7.34	0.000531	Fgf12	fibroblast growth factor 12
1426186_a_at	Mm.5055.2	-3.87	0.023517	Fgf5	fibroblast growth factor 5
1453006_at	Mm.41674.1	3	0.034212	Fgfbp3	fibroblast growth factor binding protein 3
1427846_x_at	Mm.4912.3	-4.69	0.042078	Fgfr4	fibroblast growth factor receptor 4
1419184_a_at	Mm.6799.1	7.9	0.002107	Fhl2	four and a half LIM domains 2
1419376_at	Mm.23947.1	3.3	0.041971	Fibin	fin bud initiation factor homolog (zebrafish)
1437536_at	Mm.39703.1	6.43	0.014179	Fkrp	fukutin related protein
1427268_at	Mm.4685.1	-8.59	0.015676	Flg	filaggrin
1429310_at	Mm.66061.1	-2.04	0.041498	Flrt3	fibronectin leucine rich transmembrane protein 3
1428579_at	Mm.31405.1	-4.83	0.002278	Fmnl2	formin-like 2
1421709_a_at	Mm.1668.1	4.59	0.043689	Fmo5	flavin containing monooxygenase 5
1451806_at	Mm.2035.1	-2.25	0.011418	Fnbp1	formin binding protein 1
1430119_at	Mm.195526.1	-4.35	0.003066	Fndc1	fibronectin type III domain containing 1
1445930_at	Mm.36448.1	5.08	0.006283	Fndc7	fibronectin type III domain containing 7
1430915_at	Mm.160153.1	4.83	0.014489	Fndc8	fibronectin type III domain containing 8
1439189_at	Mm.215803.1	2.49	0.021137	Fnip2	folliculin interacting protein 2
1456552_at	Mm.129645.1	5.15	0.011117	Foxl2os	forkhead box L2 opposite strand transcript
1456815_at	Mm.29347.1	-6.28	0.037305	Foxn1	forkhead box N1
1431066_at	Mm.219652.1	-2.37	0.025821	Fut11	fucosyltransferase 11
1450044_at	Mm.4770.1	-7.34	0.033862	Fzd7	frizzled homolog 7 (Drosophila)
1435021_at	Mm.32758.1	-6.91	0.011938	Gabrb3	gamma-aminobutyric acid (GABA) A receptor, subunit beta 3
1452583_s_at	Mm.29098.1	-8.5	0.034857	Galm	galactose mutarotase
1457045_at	Mm.156869.1	-4.03	0.045943	Galnt13	UDP-N-acetyl-alpha-D-galactosamine:polypeptide N-acetylgalactosaminyltra
1417399_at	Mm.3982.1	-10.04	0.01116	Gas6	growth arrest specific 6
1423992_at	Mm.21919.1	2.65	0.014383	Gatad2a	GATA zinc finger domain containing 2A
1421104_at	Mm.5057.1	-2.04	0.032898	Gbp4	guanylate binding protein 4; guanylate-binding protein 8
1450238_at	Mm.23803.1	10.61	0.005017	Gcnt2	glucosaminyl (N-acetyl) transferase 2, I-branching enzyme
1451733_at	Mm.26734.1	-2.19	0.015476	Gcnt2	glucosaminyl (N-acetyl) transferase 2, I-branching enzyme
1426063_a_at	Mm.4362.2	2.17	0.041712	Gem	GTP binding protein (gene overexpressed in skeletal muscle)
1425579_at	Mm.41886.2	-2.46	0.003483	Gfra2	glial cell line derived neurotrophic factor family receptor alpha 2
1424374_at	Mm.28395.1	2.04	0.041987	Gimap4	GTPase, IMAP family member 4
1448766_at	Mm.21198.1	6.77	0.001392	Gjb1	gap junction protein, beta 1
1448767_s_at	Mm.21198.1	2.58	0.049596	Gjb1	gap junction protein, beta 1
1450483_at	Mm.40016.1	-2.25	0.041165	Gjc2	gap junction protein, gamma 2
1449006_at	Mm.1114.1	-3.71	0.028292	Gla	galactosidase, alpha
1422330_at	Mm.140772.1	-10.97	0.001304	Glp1r	glucagon-like peptide 1 receptor
1422277_at	Mm.89320.1	-2.9	0.029108	Glra1	glycine receptor, alpha 1 subunit
1438827_at	Mm.35082.1	7.3	0.018633	Gls	glutaminase
1457211_at	Mm.209932.1	-2.23	0.017295	Gls	glutaminase
1441786_at	Mm.44801.1	-2.46	0.034084	Gm10125	predicted gene 10125
1441759_at	Mm.211606.1	-5.03	0.002767	Gm10804	predicted gene 10804
1430740_at	Mm.160290.1	3.39	0.04322	Gm11651	predicted gene 11651
1459089_at	Mm.28381.1	-5.1	0.008801	Gm11756	predicted gene 11756; predicted gene 11757; predicted gene 11758; predicted
1430731_at	Mm.51510.1	-8	0.010219	Gm11937	predicted gene 11937; predicted gene 11938; keratin associated protein 2-4
1437823_at	Mm.186038.1	2.8	0.026389	Gm12406	predicted gene 12406
1457274_at	Mm.17793.1	2.52	0.000005	Gm13103	predicted gene 13103
1444837_at	Mm.78350.1	3.01	0.032441	Gm13261	predicted gene 13261
1454275_at	Mm.158300.1	4.34	0.02597	Gm13594	predicted gene 13594
1443256_at	Mm.137360.1	12.65	0.007867	Gm16233	predicted gene 16233
1457363_at	Mm.211685.1	-5.72	0.025627	Gm17753	predicted gene, 17753
1439958_at	Mm.212423.1	2.01	0.049366	Gm1995	REX1, RNA exonuclease 1 homolog pseudogene
1446351_at	Mm.216036.1	4.24	0.005852	Gm20555	predicted gene, 20555
1445021_at	Mm.207785.1	-5.24	0.045589	Gm3650	predicted gene 3650; spastic paraplegia 11
1458529_at	Mm.185947.1	3.92	0.047942	Gm4793	predicted gene 4793
1446745_at	Mm.769.1	-3.18	0.035483	Gm5547	predicted gene 5547
1452554_at	Mm.143873.1	-2.89	0.014243	Gm8479	thiopurine S-methyltransferase pseudogene
1450373_at	Mm.3320.1	-12.65	0.003114	Gm8882	predicted gene 8882; uncharacterized LOC100502592; proline-rich protein
1460523_at	Mm.195803.1	-8.03	0.002826	Gm9777	predicted gene 9777
1443375_at	Mm.218225.1	-4.25	0.009048	Gnas	GNAS (guanine nucleotide binding protein, alpha stimulating) complex locus
1435268_at	Mm.191884.1	7.54	0.029036	Gnaz	guanine nucleotide binding protein, alpha z subunit
1425908_at	Mm.190621.1	2.54	0.006407	Gnb1	guanine nucleotide binding protein (G protein), beta 1
1448942_at	Mm.25547.1	4.03	0.017824	Gng11	guanine nucleotide binding protein (G protein), gamma 11
1417943_at	Mm.39861.1	-3.57	0.048777	Gng4	guanine nucleotide binding protein (G protein), gamma 4
1421088_at	Mm.1528.1	-8.85	0.001591	Gpc4	glypican 4
1428323_at	Mm.41718.1	-2.18	0.003693	Gpd2	glycerol phosphate dehydrogenase 2, mitochondrial
1417434_at	Mm.3711.1	-2.38	0.000105	Gpd2	glycerol phosphate dehydrogenase 2, mitochondrial
1452741_s_at	Mm.41718.1	-2.8	0.033695	Gpd2	glycerol phosphate dehydrogenase 2, mitochondrial
1453022_at	Mm.46367.1	-9.25	0.002714	Gpihbp1	GPI-anchored HDL-binding protein 1
1426442_at	Mm.178672.1	8.69	0.024064	Gpm6a	glycoprotein m6a
1421443_at	Mm.159950.1	-6.56	0.044624	Gpr110	G protein-coupled receptor 110
1421755_at	Mm.20455.1	-4.5	0.028332	Gpr132	G protein-coupled receptor 132

1442261_at	Mm.167172.1	-4.61	0.003166	Gpr150	G protein-coupled receptor 150
1442116_at	Mm.70979.1	5.28	0.006287	Gpr176	G protein-coupled receptor 176
1440623_at	Mm.208740.1	-4.89	0.00844	Gpr26	G protein-coupled receptor 26
1422542_at	Mm.89979.1	2.12	0.030327	Gpr34	G protein-coupled receptor 34
1440148_at	Mm.39665.1	-3.58	0.045194	Gpr6	G protein-coupled receptor 6
1424896_at	Mm.44103.1	-5.01	0.006976	Gpr85	G protein-coupled receptor 85
1447689_at	Mm.180744.1	-9.88	0.001251	Gprasp1	G protein-coupled receptor associated sorting protein 1
1424450_at	Mm.103456.1	2.7	0.008752	Gprc5c	G protein-coupled receptor, family C, group 5, member C
1420698_at	Mm.1332.1	-5.87	0.014363	Gpx5	glutathione peroxidase 5
1418492_at	Mm.25760.1	-2.24	0.002621	Grem2	gremlin 2 homolog, cysteine knot superfamily (Xenopus laevis)
1452643_at	Mm.219673.1	4.39	0.017164	Grhl2	grainyhead-like 2 (Drosophila)
1434728_at	Mm.32184.1	-5.92	0.001743	Gria3	glutamate receptor, ionotropic, AMPA3 (alpha 3)
1442328_at	Mm.40608.1	-7.28	0.020156	Grin2d	glutamate receptor, ionotropic, NMDA2D (epsilon 4)
1423634_at	Mm.86870.1	-2.39	0.01339	Gsdma	gasdermin A
1436013_at	Mm.39599.1	-2.93	0.003289	Gsg1l	GSG1-like
1444302_at	Mm.61160.1	-3.12	0.035576	Gxylt2	glucoside xylosyltransferase 2
1448194_a_at	Mm.14802.1	-2.52	0.047372	H19	H19 fetal liver mRNA
1435650_at	Mm.152048.1	-3.18	0.000899	Hapln4	hyaluronan and proteoglycan link protein 4
1418102_at	Mm.4451.1	2.36	0.001025	Hes1	hairy and enhancer of split 1 (Drosophila)
1442884_at	Mm.209954.1	3.53	0.008204	Hgf	hepatocyte growth factor
1425636_at	Mm.196830.1	-3.11	0.040042	Hhat	hedgehog acyltransferase
1440330_at	Mm.169486.1	-7.25	0.02309	Hist1h2bc	histone cluster 1, H2bc; histone cluster 1, H2be; histone cluster 1, H2bg
1422155_at	Mm.15595.1	2.17	0.025672	Hist1h3b	histone cluster 1,
1442798_x_at	Mm.154523.1	3.67	0.040801	Hk3	hexokinase 3
1438494_at	Mm.57240.2	-5.55	0.004121	Hrh1	histamine receptor H1
1450047_at	Mm.41264.1	-6.16	0.04522	Hs6st2	heparan sulfate 6-O-sulfotransferase 2
1428640_at	Mm.75856.1	9.28	0.009808	Hsf2bp	heat shock transcription factor 2 binding protein
1422207_at	Mm.4835.1	-5.97	0.043611	Htr5a	5-hydroxytryptamine (serotonin) receptor 5A
1444279_at	Mm.67947.1	-2.27	0.004782	Huwe1	HECT, UBA and WWE domain containing 1
1449954_at	Mm.10305.1	-2.03	0.045589	Hyal1	hyaluronoglucosaminidase 1; N-acetyltransferase 6
1450783_at	Mm.6718.1	-2.24	0.035042	Ifit1	interferon-induced protein with tetratricopeptide repeats 1
1423754_at	Mm.141021.1	2.87	0.000972	Ifitm3	interferon induced transmembrane protein 3
1416473_a_at	Mm.143741.1	2.57	0.02091	Igdcc4	immunoglobulin superfamily, DCC subclass, member 4
1452108_at	Mm.10226.1	-7.17	0.018308	Igf1r	insulin-like growth factor I receptor
1447998_at	Mm.207568.1	3.1	0.008701	Ighg3	Immunoglobulin heavy constant gamma 3
1426196_at	MmAffx.1.27	5	0.028396	Ighm	immunoglobulin heavy constant mu
1460423_x_at	Mm.104747.25	4.77	0.040059	Igk-V1	immunoglobulin kappa chain variable 1 (V1); immunoglobulin kappa chain
1425871_a_at	Mm.104747.8	-3.76	0.046017	Igk-V28	immunoglobulin kappa chain variable 28 (V28)
1447918_x_at	Mm.182172.1	-7.61	0.000936	Iglc2	immunoglobulin lambda constant 2
1456545_at	Mm.189286.1	2.07	0.048345	Il18rap	interleukin 18 receptor accessory protein
1459777_at	Mm.122420.1	5.37	0.000539	Il1r1	interleukin 1 receptor, type I
1425145_at	Mm.35692.2	-6.75	0.003895	Il1rl1	interleukin 1 receptor-like 1
1449864_at	Mm.371.1	-7.72	0.000864	Il4	interleukin 4
1426859_at	Mm.3092.1	-3.27	0.010172	Inhbb	inhibin beta-B
1457359_at	Mm.170349.1	-6.49	0.046423	Inpp4b	inositol polyphosphate-4-phosphatase, type II
1437856_at	Mm.12055.2	-2.2	0.000007	Ipmk	inositol polyphosphate multikinase
1456200_at	Mm.12055.2	-2.35	0.003165	Ipmk	inositol polyphosphate multikinase
1429577_at	Mm.75065.1	-3.17	0.033205	Iqcf4	IQ motif containing F4
1441429_at	Mm.146397.1	-2.72	0.005945	Irs4	insulin receptor substrate 4
1426298_at	Mm.28888.1	-4.45	0.014272	Irx2	Iroquois related homeobox 2 (Drosophila)
1432281_a_at	Mm.116903.3	4.03	0.046338	Itgb6	integrin beta 6
1421678_at	Mm.88406.1	-2.13	0.009905	Itpr2	inositol 1,4,5-triphosphate receptor 2
1435624_at	Mm.32206.1	-2.62	0.034984	Kazn	kazrin, periplakin interacting protein
1426070_a_at	Mm.56904.2	-4.71	0.024706	Kcnd3	potassium voltage-gated channel, Shal-related family, member 3
1459656_at	Mm.150277.1	4.19	0.000121	Kcng1	potassium voltage-gated channel, subfamily G, member 1
1450370_a_at	Mm.160172.1	-5.58	0.011249	Kcnip4	Kv channel interacting protein 4
1424848_at	Mm.4123.2	6.88	0.001688	Kcnma1	potassium large conductance calcium-activated channel, subfamily M,alpha me
1421400_at	Mm.6206.1	2.06	0.008658	Kcnmb1	potassium large conductance calcium-activated channel, subfamily M, beta me
1420800_a_at	Mm.40615.1	-3.53	0.036741	Kcnq2	potassium voltage-gated channel, subfamily Q, member 2
1459971_at	Mm.53643.1	-2.22	0.010708	Kcnt2	potassium channel, subfamily T, member 2
1453273_at	Mm.76079.1	-7.66	0.02458	Kcnv1	potassium channel, subfamily V, member 1
1458449_at	Mm.210513.2	-10.9	0.017712	Kdm2a	lysine (K)-specific demethylase 2A
1424747_at	Mm.2204.1	-2.14	0.026687	Kif1c	kinesin family member 1C
1427635_at	Mm.41576.2	10.98	0.008925	Kif5a	kinesin family member 5A
1420395_a_at	Mm.42170.1	-2.65	0.049588	Kif9	kinesin family member 9
1422227_at	Mm.42225.1	7.46	0.00489	Klf12	Kruppel-like factor 12
1456561_s_at	Mm.12014.1	-2.02	0.008464	Klf17	Kruppel-like factor 17
1441200_at	Mm.209826.1	-5.61	0.013585	Klf3	Kruppel-like factor 3 (basic)
1453495_at	Mm.20163.1	-2.26	0.012403	Klk12	kallikrein related-peptidase 12
1451607_at	Mm.143833.1	-3.81	0.020411	Klk1b21	kallikrein 1-related peptidase b21
1425259_x_at	Mm.160169.1	4.49	0.010997	Klra12	killer cell lectin-like receptor subfamily A, member 12
1426171_x_at	Mm.193478.4	3.55	0.007145	Klra7	killer cell lectin-like receptor, subfamily A, member 7
1422065_at	Mm.160364.1	-7.76	0.0006	Klrb1b	killer cell lectin-like receptor subfamily B member 1B
1419231_s_at	Mm.4201.1	-7.02	0.004557	Krt12	keratin 12
1422454_at	Mm.4646.1	-3.8	0.02304	Krt13	keratin 13
1422783_a_at	Mm.22629.1	-3.6	0.049865	Krt6a	keratin 6A
1435989_x_at	Mm.6800.3	5.15	0.02911	Krt8	keratin 8
1427801_at	Mm.215251.1	4.37	0.011612	Krtap19-1	keratin associated protein 19-1
1421691_at	Mm.215252.1	-31.77	0.028008	Krtap21-1	keratin associated protein 21-1
1444860_at	Mm.174029.1	-4.7	0.02492	Lama3	laminin, alpha 3
1427009_at	Mm.4339.1	2.5	0.034576	Lama5	laminin, alpha 5
1451758_at	Mm.88735.1	-3.51	0.019671	Lamc3	laminin gamma 3
1438687_at	Mm.36534.1	5.38	0.00382	Lax1	lymphocyte transmembrane adaptor 1
1447520_at	Mm.59984.1	-7.14	0.008447	Lbp	lipopolysaccharide binding protein
1456248_at	Mm.46390.1	4.47	0.011788	Lce3f	late cornified envelope 3F; late cornified envelope protein 3C-like
1418641_at	Mm.1781.1	2.23	0.002523	Lcp2	lymphocyte cytosolic protein 2
1439557_s_at	Mm.200482.1	-7.89	0.033004	Ldb2	LIM domain binding 2
1433783_at	Mm.29733.3	-10.06	0.030865	Ldb3	LIM domain binding 3
1459700_at	Mm.40774.1	-2.64	0.019739	Lemd3	LEM domain containing 3
1440191_s_at	Mm.45066.1	-10.53	0.027737	Leng9	leukocyte receptor cluster (LRC) member 9
1422071_at	Mm.210336.1	-2.29	0.037385	Lgals6	lectin, galactose binding, soluble 6
1433776_at	Mm.29465.1	-7.12	0.024671	Lhfp	lipoma HMGIC fusion partner
1429592_at	Mm.101478.1	-12.22	0.002118	Lhfpl3	lipoma HMGIC fusion partner-like 3
1430803_at	Mm.53701.1	10.96	0.032423	Lin28b	lin-28 homolog B (C. elegans)
1447665_at	Mm.168334.1	3.47	0.043374	Lingo2	leucine rich repeat and Ig domain containing 2
1432180_at	Mm.158548.1	-7.69	0.034594	Lipe	lipase, hormone sensitive
1452212_at	Mm.3438.3	-5.19	0.004993	Lmna	lamin A
1426868_x_at	Mm.3438.3	-10	0.022489	Lmna	lamin A
1444573_at	Mm.151794.1	-3.52	0.026654	Lmtk3	lemur tyrosine kinase 3
1421769_at	Mm.57111.1	-4.57	0.000792	Lmx1b	LIM homeobox transcription factor 1 beta

1434500_at	Mm.41871.2	-11.05	0.008152	LOC100862618	protein tweety homolog 2-like; tweety homolog 2 (Drosophila)
1432055_at	Mm.158329.1	-3.34	0.030266	LOC101056021	uncharacterized LOC101056021; SAM and SH3 domain containing 1
1435774_at	Mm.46750.1	-2.34	0.039961	LOC106740	uncharacterized LOC106740; PHD finger protein 10
1458505_at	Mm.102811.1	5.52	0.042625	LOC552901	uncharacterized LOC552901
1445422_at	Mm.169657.1	-2.01	0.002361	LOC621549	uncharacterized LOC621549
1455665_at	Mm.41111.1	-4.92	0.006885	Lonrf1	LON peptidase N-terminal domain and ring finger 1
1418723_at	Mm.155520.1	2.4	0.010402	Lpar3	lysophosphatidic acid receptor 3
1434945_at	Mm.21463.1	-5.86	0.003982	Lpcat2	lysophosphatidylcholine acyltransferase 2
1429368_at	Mm.124830.1	2.47	0.036735	Lrig3	leucine-rich repeats and immunoglobulin-like domains 3
1459446_at	Mm.209717.1	-5.71	0.002234	Lrrc41	leucine rich repeat containing 41
1432716_at	Mm.158868.1	4.97	0.045549	Lrrn1	leucine rich repeat protein 1, neuronal
1456637_at	Mm.18962.4	5.38	0.041392	Lrrtm2	leucine rich repeat transmembrane neuronal 2
1417756_a_at	Mm.2183.1	2.29	0.022062	Lsp1	lymphocyte specific 1
1420353_at	Mm.87787.1	4.34	0.013721	Lta	lymphotoxin A
1459552_at	Mm.210132.1	-4.38	0.047302	Lurap1l	leucine rich adaptor protein 1-like
1453304_s_at	Mm.204648.1	2.66	0.022126	Ly6e	lymphocyte antigen 6 complex, locus E
1422625_at	Mm.22154.1	-5.06	0.049341	Ly6h	lymphocyte antigen 6 complex, locus H
1449328_at	Mm.2074.1	-2.23	0.022609	Ly75	lymphocyte antigen 75
1451715_at	Mm.67919.1	-2.33	0.031824	Mafb	v-maf musculoaponeurotic fibrosarcoma oncogene family, protein B (avian)
1423284_at	Mm.57648.1	-6.59	0.004074	Mansc1	MANSC domain containing 1
1421850_at	Mm.4323.1	2.27	0.044711	Map1b	microtubule-associated protein 1B
1448871_at	Mm.27970.1	2.04	0.018996	Mapk13	mitogen-activated protein kinase 13
1442553_at	Mm.209148.1	-10.28	0.039095	Mapre2	microtubule-associated protein, RP/EB family, member 2
1453264_at	Mm.31998.1	-6.88	0.013356	Marveld3	MARVEL (membrane-associating) domain containing 3
1433394_at	Mm.101107.1	-5.9	0.000767	Mast4	microtubule associated serine/threonine kinase family member 4
1421739_a_at	Mm.2918.1	3.82	0.04646	Matk	megakaryocyte-associated tyrosine kinase
1447846_x_at	Mm.161902.1	-5.64	0.003443	Mboat7	membrane bound O-acyltransferase domain containing 7
1427755_at	Mm.42052.1	7.89	0.001298	Mcpt-ps1	mast cell protease, pseudogene 1
1451719_at	Mm.44151.1	-2.05	0.032003	Med17	mediator complex subunit 17
1440091_at	Mm.209554.1	-6.89	0.014185	Meis2	Meis homeobox 2
1439351_at	Mm.208445.1	-3.48	0.024152	Mfsd4	major facilitator superfamily domain containing 4
1458903_at	Mm.191681.1	11.95	0.010284	Miat	myocardial infarction associated transcript (non-protein coding)
1435645_at	Mm.139683.1	2.09	0.029464	Mmd	monocyte to macrophage differentiation-associated
1418193_at	Mm.3759.1	-5.7	0.000662	Mnt	max binding protein
1452804_at	Mm.33810.1	-3.21	0.02615	Morn5	MORN repeat containing 5
1450775_at	Mm.26262.1	4.55	0.022495	Mos	Moloney sarcoma oncogene
1444306_at	Mm.209316.1	5.03	0.035066	Msi1	musashi RNA-binding protein 1
1421687_at	Mm.2540.1	-4	0.039383	Msmb	beta-microseminoprotein
1452990_at	Mm.24388.1	-3.45	0.029438	Mtf1	metal response element binding transcription factor 1
1446284_at	Mm.215481.1	-2.9	0.006372	Mtss1	metastasis suppressor 1
1451905_a_at	Mm.33996.2	-3.01	0.023097	Mx1	myxovirus (influenza virus) resistance 1
1427940_s_at	Mm.220922.1	-2.34	0.001046	Mycbp	c-myc binding protein
1452608_at	Mm.220922.1	-2.5	0.000025	Mycbp	c-myc binding protein
1435463_s_at	Mm.6493.1	-2.36	0.032574	Myo1d	myosin ID
1439708_at	Mm.66385.1	2.33	0.008547	Myom3	myomesin family, member 3
1444657_at	Mm.208892.1	-3.04	0.02983	N4bp2	NEDD4 binding protein 2
1437287_at	Mm.42110.2	-3.23	0.000063	Nadk2	NAD kinase 2, mitochondrial
1454748_at	Mm.25245.1	7.38	0.020466	Naprt1	nicotinate phosphoribosyltransferase domain containing 1
1442918_at	Mm.71029.1	2.75	0.026214	Nav3	neuron navigator 3
1422044_at	Mm.42245.1	4.87	0.020561	Ndst1	N-deacetylase/N-sulfotransferase (heparan glucosaminyl) 1
1453777_a_at	Mm.132056.2	-5.87	0.00997	Ndst3	N-deacetylase/N-sulfotransferase (heparan glucosaminyl) 3
1434905_at	Mm.45843.1	-2.18	0.019682	Ndufa4l2	NADH dehydrogenase (ubiquinone) 1 alpha subcomplex, 4-like 2
1423561_at	Mm.3959.1	3.18	0.027466	Nell2	NEL-like 2
1436309_at	Mm.27143.1	3.05	0.019296	Neto2	neuropilin (NRP) and tolloid (TLL)-like 2
1453527_a_at	Mm.103587.4	2.58	0.046061	Neurl1a	neuralized homolog 1A (Drosophila)
1448966_a_at	Mm.34530.1	5.86	0.014593	Nfat5	nuclear factor of activated T cells 5
1426031_a_at	Mm.116802.4	-3.73	0.040326	Nfatc2	nuclear factor of activated T cells, cytoplasmic, calcineurin dependent 2
1456087_at	Mm.31274.1	2.5	0.005351	Nfia	nuclear factor I/A
1429148_at	Mm.34042.1	3.14	0.036223	Nfic	nuclear factor I/C
1422565_s_at	Mm.5104.1	-8.99	0.004609	Nfic	nuclear factor I/C
1448728_a_at	Mm.3732.1	-2.38	0.009853	Nfkbiz	nuclear factor of kappa light polypeptide gene enhancer in B cells inhibitor,zeta
1423516_a_at	Mm.20348.2	-2.11	0.017786	Nid2	nidogen 2
1417278_a_at	Mm.30219.1	-3.68	0.033776	Nkd1	naked cuticle 1 homolog (Drosophila)
1422050_at	Mm.4293.1	-3.78	0.037098	Nkx1-2	NK1 transcription factor related, locus 2 (Drosophila)
1438077_at	Mm.22313.1	-3.83	0.009805	Nlrp4a	NLR family, pyrin domain containing 4A
1447503_at	Mm.45363.1	-3.5	0.017176	Nmrk1	nicotinamide riboside kinase 1
1422300_at	Mm.39094.1	-5.11	0.025689	Nog	noggin
1447268_at	Mm.212449.1	2.25	0.040106	Nol12	nucleolar protein 12
1437605_at	Mm.220908.1	-14.21	0.002135	Nphs2	nephrosis 2 homolog, podocin (human)
1419008_at	Mm.10685.1	13.3	0.004309	Npy5r	neuropeptide Y receptor Y5
1451807_at	Mm.8509.1	-2.18	0.026861	Nr1i2	nuclear receptor subfamily 1, group I, member 2
1438796_at	Mm.38274.1	7.52	0.016476	Nr4a3	nuclear receptor subfamily 4, group A, member 3
1436399_s_at	Mm.200428.1	-11.36	0.026147	Nrk	Nik related kinase
1447717_x_at	Mm.131014.1	-8.26	0.012018	Nrsn2	neurensin 2
1439358_a_at	Mm.39843.2	-2.47	0.013457	Nrxn1	neurexin I
1432931_at	Mm.159202.1	6.23	0.027112	Nrxn3	neurexin III
1424882_a_at	Mm.30083.1	7.48	0.000385	Nt5dc2	5'-nucleotidase domain containing 2
1456305_x_at	Mm.26391.3	2.97	0.012293	Obox2	oocyte specific homeobox 2
1443632_at	Mm.33325.1	-5.07	0.035416	Obscn	obscurin, cytoskeletal calmodulin and titin-interacting RhoGEF
1443671_x_at	Mm.46278.2	-3.87	0.024272	Odf3b	outer dense fiber of sperm tails 3B
1437060_at	Mm.26456.1	5.21	0.021513	Olfm4	olfactomedin 4
1450600_at	Mm.88823.1	-2.93	0.018166	Olfr1508	olfactory receptor 1508
1443936_at	Mm.100857.1	3.54	0.049626	Olfr976	olfactory receptor 976
1422200_at	Mm.57036.1	-3.09	0.004379	Omp	olfactory marker protein
1455041_at	Mm.3765.2	-9.46	0.040345	Omt2b	oocyte maturation, beta
1424359_at	Mm.41308.1	4.52	0.00367	Oplah	5-oxoprolinase (ATP-hydrolysing)
1451054_at	Mm.4777.1	6.58	0.041045	Orm1	orosomucoid 1
1450611_at	Mm.57239.1	5.89	0.027523	Orm3	orosomucoid 3
1457654_at	Mm.87508.1	-5.84	0.006645	Osgep	O-sialoglycoprotein endopeptidase
1449350_at	Mm.45055.1	-13.21	0.001351	Osr1	odd-skipped related 1 (Drosophila)
1446023_at	Mm.198025.1	-2.84	0.03588	Ostf1	osteoclast stimulating factor 1
1419052_at	Mm.38323.1	-5.85	0.003652	Ovol1	OVO homolog-like 1 (Drosophila)
1460719_a_at	Mm.25722.1	2.85	0.039197	P2rx1	purinergic receptor P2X, ligand-gated ion channel, 1
1450318_a_at	Mm.3000.1	-2.16	0.029387	P2ry2	purinergic receptor P2Y, G-protein coupled 2
1425214_at	Mm.32929.1	10.78	0.043074	P2ry6	pyrimidinergic receptor P2Y, G-protein coupled, 6
1417923_at	Mm.3392.1	-8.48	0.017256	Pak3	p21 protein (Cdc42/Rac)-activated kinase 3
1455847_at	Mm.124661.1	-2.01	0.023532	Pak7	p21 protein (Cdc42/Rac)-activated kinase 7
1448134_at	Mm.3437.1	-4.36	0.009423	Pald1	phosphatase domain containing, paladin 1
1432860_at	Mm.158741.1	4.85	0.007871	Panx3	pannexin 3
1432548_at	Mm.158798.1	-5.38	0.048617	Parp14	poly (ADP-ribose) polymerase family, member 14

1446561_at	Mm.216055.1	-6.07	0.042952	Pax8	paired box gene 8
1449054_a_at	Mm.29707.1	2.09	0.034377	Pcbp4	poly(rC) binding protein 4
1441358_at	Mm.183574.1	-14.46	0.000573	Pcdhb16	protocadherin beta 16
1421548_at	Mm.197508.1	-5.89	0.017606	Pcdhb2	protocadherin beta 2
1452913_at	Mm.28040.1	-8.79	0.001653	Pcp4l1	Purkinje cell protein 4-like 1
1431741_a_at	Mm.988.4	-3.09	0.034419	Pdzd9	PDZ domain containing 9
1459398_at	Mm.209921.1	-8.58	0.000674	Peli1	pellino 1
1418210_at	Mm.20399.1	-2.61	0.005872	Pfn2	profilin 2
1436993_x_at	Mm.20399.2	-2.61	0.012233	Pfn2	profilin 2
1418209_a_at	Mm.20399.1	-3	0.009242	Pfn2	profilin 2
1437871_at	Mm.86380.1	2.07	0.000131	Pgm5	phosphoglucomutase 5
1449002_at	Mm.34346.1	-2.29	0.009894	Phlda3	pleckstrin homology-like domain, family A, member 3
1453839_a_at	Mm.30950.2	4.51	0.049566	Pi16	peptidase inhibitor 16
1431917_at	Mm.6354.3	9.55	0.010882	Pigq	phosphatidylinositol glycan anchor biosynthesis, class Q
1421704_a_at	Mm.10301.1	-2.35	0.027501	Pik3c2g	phosphatidylinositol 3-kinase, C2 domain containing, gamma polypeptide
1446974_at	Mm.217894.1	-2.62	0.016305	Pikfyve	phosphoinositide kinase, FYVE finger containing
1427614_at	Mm.214755.1	-4.87	0.026896	Pip	prolactin induced protein
1427438_at	Mm.207042.1	-12.91	8.33E-07	Pip4k2b	phosphatidylinositol-5-phosphate 4-kinase, type II, beta
1451502_at	Mm.4214.1	-2.16	0.012525	Pla2g10	phospholipase A2, group X
1420674_at	Mm.89992.1	-2.14	0.039244	Pla2g2e	phospholipase A2, group IIE
1417814_at	Mm.153446.1	-5.42	0.040164	Pla2g5	phospholipase A2, group V
1443943_at	Mm.173697.1	-12.66	0.001041	Plag1	pleiomorphic adenoma gene 1
1438588_at	Mm.132189.1	-8.15	0.048362	Plagl1	pleiomorphic adenoma gene-like 1
1422138_at	Mm.4183.1	2.5	0.034351	Plau	plasminogen activator, urokinase
1430666_at	Mm.133334.1	2.17	0.044906	Plb1	phospholipase B1
1431892_a_at	Mm.54993.1	3.07	0.021981	Plcd3	phospholipase C, delta 3
1437030_at	Mm.100350.1	-2.97	0.01797	Plcd4	phospholipase C, delta 4
1437842_at	Mm.133794.1	-5	0.027034	Plcxd1	phosphatidylinositol-specific phospholipase C, X domain containing 1
1433678_at	Mm.203915.1	2.37	0.009496	Pld4	phospholipase D family, member 4
1436128_at	Mm.182164.1	-5.29	0.03107	Plekha8	pleckstrin homology domain containing, family A (phosphoinositide binding
1416178_a_at	Mm.26633.1	2.01	0.048364	Plekhb1	pleckstrin homology domain containing, family B (evectins) member 1
1460332_at	Mm.34145.1	-5.34	0.043025	Pln	phospholamban
1416686_at	Mm.79983.1	-2.36	0.011063	Plod2	procollagen lysine, 2-oxoglutarate 5-dioxygenase 2
1416687_at	Mm.79983.1	-2.99	0.02807	Plod2	procollagen lysine, 2-oxoglutarate 5-dioxygenase 2
1460269_at	Mm.213024.1	3.64	0.032673	Pnmt	phenylethanolamine-N-methyltransferase
1416573_at	Mm.203556.1	2.06	0.004898	Pofut2	protein O-fucosyltransferase 2
1447864_s_at	Mm.90592.1	5.43	0.037052	Pogk	pogo transposable element with KRAB domain
1424220_a_at	Mm.103707.1	3.54	0.007547	Porcn	porcupine homolog (Drosophila)
1422220_at	Mm.4726.1	-9.71	0.018054	Pou1f1	POU domain, class 1, transcription factor 1
1431092_at	Mm.200527.1	-2.15	0.04622	Ppp1r12c	protein phosphatase 1, regulatory (inhibitor) subunit 12C
1425414_at	Mm.150540.1	-2.51	0.017057	Ppp1r16b	protein phosphatase 1, regulatory (inhibitor) subunit 16B
1425725_s_at	Mm.3785.2	-2.09	0.004298	Ppp2r5c	protein phosphatase 2, regulatory subunit B (B56), gamma isoform
1455996_x_at	Mm.141646.2	2.8	0.031592	Prap1	proline-rich acidic protein 1
1457197_at	Mm.133396.1	-5.12	0.02928	Prkacb	protein kinase, cAMP dependent, catalytic, beta
1434325_x_at	Mm.9334.2	-3.88	0.003401	Prkar1b	protein kinase, cAMP dependent regulatory, type I beta
1427562_a_at	Mm.1266.2	-2.65	0.011248	Prkca	protein kinase C, alpha
1443144_at	Mm.207496.1	-12.45	0.016074	Prkcb	protein kinase C, beta
1429287_a_at	Mm.1270.2	4.21	0.049945	Prl	prolactin
1435642_at	Mm.40741.1	-3.01	0.011522	Prr18	proline rich region 18
1433459_x_at	Mm.14410.2	-9.75	0.006229	Prss2	protease, serine, 2
1450802_at	Mm.207081.1	3.99	0.025579	Prss28	protease, serine, 28
1420279_at	Mm.56997.1	-3.93	0.027124	Prss58	protease, serine 58
1438333_at	Mm.103497.1	-2.07	0.012161	Prtg	protogenin homolog (Gallus gallus)
1416240_at	Mm.2246.1	2.19	0.045645	Psmb7	proteasome (prosome, macropain) subunit, beta type 7
1421410_a_at	Mm.57174.1	-2.8	0.000583	Pstpip2	proline-serine-threonine phosphatase-interacting protein 2
1445703_at	Mm.39614.1	-5.33	0.013425	Ptchd1	patched domain containing 1
1431518_at	Mm.61213.1	-4.39	0.01233	Ptchd3	patched domain containing 3
1449739_at	Mm.201436.1	10.37	0.004381	Ptdss1	phosphatidylserine synthase 1
1446331_at	Mm.49651.1	-7.99	0.019467	Ptgfr	prostaglandin F receptor
1436448_a_at	Mm.2792.2	2.37	0.00029	Ptgs1	prostaglandin-endoperoxide synthase 1
1417092_at	Mm.3542.1	-8.35	0.004644	Pth1r	parathyroid hormone 1 receptor
1440082_at	Mm.103015.1	-8.66	0.04812	Ptk2	PTK2 protein tyrosine kinase 2
1448254_at	Mm.3063.1	7.42	0.033211	Ptn	pleiotrophin
1445362_at	Mm.209904.1	-3.56	0.019785	Ptpdc1	protein tyrosine phosphatase domain containing 1
1445767_at	Mm.129782.1	5.82	0.030915	Ptprd	protein tyrosine phosphatase, receptor type, D
1423277_at	Mm.27856.1	-4.23	0.030111	Ptprk	protein tyrosine phosphatase, receptor type, K
1422541_at	Mm.37854.1	2.74	0.034552	Ptprm	protein tyrosine phosphatase, receptor type, M
1457257_x_at	Mm.40477.1	-4.71	0.000043	Pvrl3	poliovirus receptor-related 3
1425597_a_at	Mm.2655.3	-5.8	0.011681	Qk	quaking
1438252_at	Mm.27035.2	4.36	0.038275	Qsox1	quiescin Q6 sulfhydryl oxidase 1
1425285_a_at	Mm.34867.2	2.99	0.002277	Rab27a	RAB27A, member RAS oncogene family
1439610_at	Mm.100092.1	-11.78	0.000467	Rab27b	RAB27b, member RAS oncogene family
1437762_at	Mm.132695.1	2.09	0.049326	Rab39	RAB39, member RAS oncogene family
1437107_at	Mm.193647.1	-3.29	0.047803	Rab6b	RAB6B, member RAS oncogene family
1449331_a_at	Mm.1272.1	2.3	0.010823	Rapsn	receptor-associated protein of the synapse
1428538_s_at	Mm.28231.1	-2.51	0.017641	Rarres2	retinoic acid receptor responder (tazarotene induced) 2
1456491_at	Mm.39720.2	-2.36	0.028767	Rbm24	RNA binding motif protein 24
1458624_at	Mm.101192.2	-2.39	0.024395	Rbm24	RNA binding motif protein 24
1416297_s_at	Mm.2553.1	-6.76	0.002348	Reg3b	regenerating islet-derived 3 beta
1433416_at	Mm.158691.1	-4.33	0.010915	Rfx2	regulatory factor X, 2 (influences HLA class II expression)
1439779_at	Mm.80604.1	-6.03	0.007608	Rgs17	regulator of G-protein signaling 17
1440618_at	Mm.100473.1	4.35	0.048979	Rhbdl2	rhomboid, veinlet-like 2 (Drosophila)
1419061_at	Mm.27701.1	-4.55	0.038167	Rhod	ras homolog gene family, member D
1449028_at	Mm.28954.1	2.42	0.043211	Rhou	ras homolog gene family, member U
1434628_a_at	Mm.35465.4	-5.82	0.023195	Rhpn2	rhophilin, Rho GTPase binding protein 2
1427580_a_at	Mm.23086.1	3.03	0.046557	Rian	RNA imprinted and accumulated in nucleus
1435667_at	Mm.40425.1	-4.4	0.005238	Rims1	regulating synaptic membrane exocytosis 1
1426368_at	Mm.3069.1	4.47	0.004258	Rin2	Ras and Rab interactor 2
1452300_at	Mm.78312.1	-11.31	0.000158	Rmdn1	regulator of microtubule dynamics 1
1455197_at	Mm.100439.1	-7.36	0.014269	Rnd1	Rho family GTPase 1
1443407_at	Mm.215848.1	-4.83	0.030743	Rnf13	ring finger protein 13
1448714_at	Mm.26153.1	-2.1	0.001743	Rngtt	RNA guanylyltransferase and 5'-phosphatase
1458229_at	Mm.213321.1	-8.49	0.003583	Robo2	roundabout homolog 2 (Drosophila)
1457128_at	Mm.204166.1	-2.3	0.006018	Ror2	receptor tyrosine kinase-like orphan receptor 2
1424034_at	Mm.8858.2	-6.46	0.007464	Rora	RAR-related orphan receptor alpha
1458472_at	Mm.209933.1	-3.85	0.025894	Rptor	regulatory associated protein of MTOR, complex 1
1430784_a_at	Mm.24372.1	-6.2	0.006824	Rptor	regulatory associated protein of MTOR, complex 1
1431164_at	Mm.158423.1	-5.26	0.022357	Rragd	Ras-related GTP binding D
1440073_at	Mm.209088.1	-12.93	0.000896	Rrm1	ribonucleotide reductase M1
1417643_at	Mm.12743.1	-6.44	0.003955	Rsph1	radial spoke head 1 homolog (Chlamydomonas)

1455893_at	Mm.6149.1	10.22	0.006479	Rspo2	R-spondin 2 homolog (Xenopus laevis)
1430497_at	Mm.160121.1	4.83	0.022479	Rxra	retinoid X receptor alpha
1447400_at	Mm.213621.1	4.54	0.043799	Ryk	receptor-like tyrosine kinase
1424542_at	Mm.3925.1	5.39	0.03135	S100a4	S100 calcium binding protein A4
1419394_s_at	Mm.21567.1	4.44	0.018898	S100a8	S100 calcium binding protein A8 (calgranulin A)
1424152_at	Mm.28388.1	5.47	0.01514	Sall4	sal-like 4 (Drosophila)
1437887_at	Mm.101115.2	-10.21	0.007212	Samd5	sterile alpha motif domain containing 5
1438615_x_at	Mm.133762.1	-2.04	0.019709	Sapcd2	suppressor APC domain containing 2
1445644_at	Mm.151693.1	-5.99	0.010404	Sarm1	sterile alpha and HEAT/Armadillo motif containing 1
1430318_at	Mm.20155.1	-6.5	0.001916	Sat2	spermidine/spermine N1-acetyl transferase 2
1423366_at	Mm.95735.1	-2.77	0.00155	Scd3	stearoyl-coenzyme A desaturase 3
1428006_at	Mm.68989.1	-2.12	0.013462	Scfd1	Sec1 family domain containing 1
1450276_a_at	Mm.2416.1	2.01	0.000642	Scin	scinderin
1439554_at	Mm.83648.1	-2.43	0.012815	Scmh1	sex comb on midleg homolog 1
1436084_at	Mm.41130.1	-4.87	0.001144	Scrt1	scratch homolog 1, zinc finger protein (Drosophila)
1440930_a_at	Mm.45098.1	-4.47	0.040655	Scrt2	scratch homolog 2, zinc finger protein (Drosophila)
1419481_at	Mm.1461.1	2.47	0.012247	Sell	selectin, lymphocyte
1420508_at	Mm.12903.1	-2.54	0.028437	Sema3f	sema domain, immunoglobulin domain (Ig), (semaphorin) 3F
1437422_at	Mm.54181.1	-2.31	0.029633	Sema5a	sema domain, seven thrombospondin repeats (semaphorin) 5A
1435963_at	Mm.40254.1	3.33	0.036771	Sema5b	sema domain, seven thrombospondin repeats(semaphorin) 5B
1450419_at	Mm.199675.1	7.52	0.007791	Serhl	serine hydrolase-like
1434382_at	Mm.45132.1	4.68	0.004866	Serinc2	serine incorporator 2
1435887_at	Mm.38201.1	4.6	0.007216	Serpina11	serine (or cysteine) peptidase inhibitor, clade A (alpha-1 antiproteinase
1458882_at	Mm.30916.1	7.1	0.005857	Serpinb8	serine (or cysteine) peptidase inhibitor, clade B, member 8
1457200_at	Mm.218365.1	2.69	0.038583	Serpinb9d	serine (or cysteine) peptidase inhibitor, clade B, member 9d
1420378_at	Mm.1321.1	-3.7	0.043637	Sftpd	surfactant associated protein D
1455512_at	Mm.41587.1	-2.21	0.015404	Shisa6	shisa homolog 6 (Xenopus laevis)
1449967_at	Mm.4774.1	-6.11	0.046728	Sim1	single-minded homolog 1 (Drosophila)
1458683_at	Mm.37579.1	3.65	0.029579	Sirpb1a	signal-regulatory protein beta 1A
1427277_at	Mm.4645.1	-5.69	0.000778	Six1	sine oculis-related homeobox 1
1441925_at	Mm.169489.1	5.83	0.028298	Slc15a4	solute carrier family 15, member 4
1417280_at	Mm.2656.1	-9.73	0.000065	Slc17a1	solute carrier family 17 (sodium phosphate), member 1
1428986_at	Mm.41613.1	2.86	0.048754	Slc17a7	solute carrier family 17 (sodium-dependent inorganic phosphate cotransporter)
1426595_at	Mm.19301.1	-2.48	0.009105	Slc18a1	solute carrier family 18 (vesicular monoamine), member 1
1422203_at	Mm.57020.1	2.6	0.0044	Slc18a3	solute carrier family 18 (vesicular monoamine), member 3
1460386_a_at	Mm.24741.3	3.24	0.036626	Slc1a1	solute carrier family 1 (neuronal/epithelial high affinity glutamate transporter
1453004_at	Mm.23932.1	-8.35	0.048634	Slc22a23	solute carrier family 22, member 23
1417072_at	Mm.30090.1	-6.91	0.027396	Slc22a6	solute carrier family 22 (organic anion transporter), member 6
1424308_at	Mm.86818.1	-2.12	0.032339	Slc24a3	solute carrier family 24 (sodium/potassium/calcium exchanger), member 3
1425948_a_at	Mm.41116.2	-4.41	0.049702	Slc25a30	solute carrier family 25, member 30
1425915_at	Mm.219530.1	-5.07	0.042944	Slc26a8	solute carrier family 26, member 8
1456457_at	Mm.7444.2	-2.43	0.032573	Slc32a1	solute carrier family 32 (GABA vesicular transporter), member 1
1456764_at	Mm.40542.1	-2.69	0.030148	Slc35f3	solute carrier family 35, member F3
1429523_a_at	Mm.22983.1	4.06	0.004742	Slc39a5	solute carrier family 39 (metal ion transporter), member 5
1454344_at	Mm.159055.1	-4.7	0.013939	Slc44a1	solute carrier family 44, member 1
1418395_at	Mm.100741.1	3.18	0.04083	Slc47a1	solute carrier family 47, member 1
1417150_at	Mm.3907.1	-7.37	0.027327	Slc6a4	solute carrier family 6 (neurotransmitter transporter, serotonin), member 4
1420148_at	Mm.206905.1	2.16	0.009657	Slc6a6	solute carrier family 6 (neurotransmitter transporter, taurine), member 6
1450703_at	Mm.4676.1	-5.59	0.004714	Slc7a2	solute carrier family 7 (cationic amino acid transporter, y+ system), member 2
1439369_x_at	Mm.21587.4	-3.45	0.029908	Slc9a3r2	solute carrier family 9 (sodium/hydrogen exchanger), member 3 regulator 2
1449844_at	Mm.103665.1	4.18	0.000478	Slco1a1	solute carrier organic anion transporter family, member 1a1
1449203_at	Mm.190930.1	-7.22	0.007049	Slco1a5	solute carrier organic anion transporter family, member 1a5
1450165_at	Mm.42124.1	-3.2	0.000752	Slfn2	schlafen 2
1437037_x_at	Mm.29827.2	4.91	0.042599	Snd1	staphylococcal nuclease and tudor domain containing 1
1422310_at	Mm.219465.1	-3.12	0.049445	Snn	stannin
1416360_at	Mm.33721.1	-4.43	0.012278	Snx18	sorting nexin 18
1417633_at	Mm.2407.1	5.99	0.020994	Sod3	superoxide dismutase 3, extracellular
1429053_at	Mm.33421.1	-4.39	0.027028	Soga2	SOGA family member 2
1425369_a_at	Mm.1493.2	-2.27	0.010701	Sox10	SRY-box containing gene 10
1449135_at	Mm.2878.1	2.2	0.032076	Sox18	SRY-box containing gene 18
1458332_x_at	Mm.140916.1	5.09	0.029817	Sox4	SRY-box containing gene 4
1431958_at	Mm.159631.1	-5.45	0.011684	Spag16	sperm associated antigen 16
1417020_at	Mm.45833.1	-3.38	0.016145	Spata4	spermatogenesis associated 4
1456866_x_at	Mm.45613.1	8.13	0.0386	Spatc1l	spermatogenesis and centriole associated 1 like
1427834_at	Mm.206796.1	-6.19	0.016758	Spi16	serine protease inhibitor 16
1458174_at	Mm.215883.1	2.05	0.025104	Spice1	spindle and centriole associated protein 1
1458203_at	Mm.208723.1	-5.38	0.035237	Spire1	spire homolog 1 (Drosophila)
1450653_at	Mm.23520.1	12.54	0.021043	Spz1	spermatogenic leucine zipper 1
1428372_at	Mm.1075.1	2.77	0.002759	St5	suppression of tumorigenicity 5
1417617_at	Mm.3947.1	-4.89	0.046262	St6galnac2	ST6 (alpha-N-acetyl-neuraminyl-2,3-beta-galactosyl-1,3)
1459427_at	Mm.204724.1	-3.39	0.013698	Stam	signal transducing adaptor molecule (SH3 domain and ITAM motif) 1
1416977_at	Mm.45048.1	-5.63	0.028207	Stam2	signal transducing adaptor molecule (SH3 domain and ITAM motif) 2
1459437_at	Mm.207721.1	-3.38	0.021631	Stard9	START domain containing 9
1445186_at	Mm.213334.1	-7.9	0.000591	Stc2	stanniocalcin 2
1446071_at	Mm.39696.1	-9.65	0.048293	Steap2	six transmembrane epithelial antigen of prostate 2
1456818_at	Mm.44386.1	4.7	0.04443	Stk32a	serine/threonine kinase 32A
1439938_at	Mm.77769.1	-2.12	0.033481	Stk38	serine/threonine kinase 38
1457778_at	Mm.126705.1	-3.98	0.000533	Stox2	storkhead box 2
1437190_at	Mm.20114.1	5.1	0.022425	Styk1	serine/threonine/tyrosine kinase 1
1433714_at	Mm.29330.1	-5.07	0.024124	Sult4a1	sulfotransferase family 4A, member 1
1433374_at	Mm.159980.1	-5.17	0.016569	Supt7l	suppressor of Ty 7-like
1437387_at	Mm.184472.1	-2.75	0.006312	Susd5	sushi domain containing 5
1434800_at	Mm.39888.1	2.54	0.000099	Sv2b	synaptic vesicle glycoprotein 2 b
1428685_at	Mm.46102.1	-4.5	0.039954	Syce1	synaptonemal complex central element protein 1
1425217_a_at	Mm.30717.1	-4.62	0.013552	Synj2	synaptojanin 2
1449206_at	Mm.20942.1	-3.48	0.00582	Sypl2	synaptophysin-like 2
1429314_at	Mm.218599.1	-4.77	0.016688	Syt11	synaptotagmin XI
1417708_at	Mm.4824.1	-5.11	0.000292	Syt3	synaptotagmin III
1423323_at	Mm.154045.1	7.65	0.005657	Tacstd2	tumor-associated calcium signal transducer 2
1435675_at	Mm.41119.1	-8.43	0.000923	Tbc1d12	TBC1D12: TBC1 domain family, member 12
1457186_at	Mm.131416.1	-2.6	0.02934	Tbl1xr1	transducin (beta)-like 1X-linked receptor 1
1447660_at	Mm.180612.1	7.84	0.00953	Tbl3	transducin (beta)-like 3
1453351_at	Mm.117009.1	-3.65	0.047833	Tbx20	T-box 20
1456033_at	Mm.148483.1	2.52	0.030639	Tbx4	T-box 4
1419222_at	Mm.4545.1	2.71	0.025962	Tbxa2r	thromboxane A2 receptor
1420423_at	Mm.103652.1	-3.42	0.015583	Tcl1b4	T cell leukemia/lymphoma 1B, 4
1437482_at	Mm.129498.1	-3.11	0.005842	Tecrl	trans-2,3-enoyl-CoA reductase-like
1456941_at	Mm.38000.1	-2.04	0.032014	Tert	telomerase reverse transcriptase
1424246_a_at	Mm.88645.1	2.51	0.03389	Tes	testis derived transcript
1418744_s_at	Mm.26378.1	-5.27	0.046387	Tesc	tescalcin

1420436_x_at	Mm.78551.1	-7.66	0.002348	Tex21	testis expressed gene 21
1418147_at	Mm.3629.1	4.93	0.030619	Tfap2c	transcription factor AP-2, gamma
1417455_at	Mm.1291.1	2.2	0.031502	Tgfb3	transforming growth factor, beta 3
1420893_a_at	Mm.197552.1	-2.41	0.030841	Tgfbr1	transforming growth factor, beta receptor I
1431211_s_at	Mm.180200.1	-5.3	0.009774	Them5	thioesterase superfamily member 5
1436423_at	Mm.116681.1	-5.25	0.00168	Themis	thymocyte selection associated
1423135_at	Mm.3951.1	2.8	0.035038	Thy1	thymus cell antigen 1, theta
1426352_s_at	Mm.4363.2	-6.67	0.000856	Tial1	Tia1 cytotoxic granule-associated RNA binding protein-like 1
1441819_x_at	Mm.200556.1	-2.06	0.005089	Timm50	translocase of inner mitochondrial membrane 50
1460227_at	Mm.8245.1	-2.11	0.036956	Timp1	tissue inhibitor of metalloproteinase 1
1429111_at	Mm.33997.1	2.86	0.004007	Tln2	talin 2
1421626_at	Mm.219585.1	-10.1	0.004529	Tmc1	transmembrane channel-like gene family 1
1434252_at	Mm.205458.1	4.25	0.013748	Tmcc3	transmembrane and coiled coil domains 3
1436916_at	Mm.32665.1	-6.79	0.038946	Tmem108	transmembrane protein 108
1451344_at	Mm.41681.1	2.44	0.002069	Tmem119	transmembrane protein 119
1428074_at	Mm.8569.1	-2.36	0.00722	Tmem158	transmembrane protein 158
1448924_at	Mm.10068.1	-2.34	0.003337	Tmem186	transmembrane protein 186
1420192_at	Mm.196501.2	4.12	0.04874	Tmem191c	transmembrane protein 191C
1438914_at	Mm.205569.3	6.03	0.0027	Tmem206	transmembrane protein 206
1429261_at	Mm.45147.1	-2.91	0.039353	Tmem238	transmembrane protein 238
1436283_at	Mm.44237.1	-11.07	0.041806	Tmem52b	transmembrane protein 52B
1417895_a_at	Mm.25295.1	-2.95	0.012293	Tmem54	transmembrane protein 54
1452943_at	Mm.37515.1	-8.9	0.003449	Tmem65	transmembrane protein 65
1420726_x_at	Mm.139078.1	-5.8	0.040915	Tmlhe	trimethyllysine hydroxylase, epsilon
1422038_a_at	Mm.156947.1	6.26	0.000641	Tnfrsf22	tumor necrosis factor receptor superfamily, member 22
1450798_at	Mm.183266.1	-2.85	0.044532	Tnxb	tenascin XB
1458032_at	Mm.136648.1	-4.21	0.026393	Tpd52l2	tumor protein D52-like 2
1425028_a_at	Mm.646.2	4.63	0.020898	Tpm2	tropomyosin 2, beta
1449996_a_at	Mm.17306.1	2.19	0.01983	Tpm3	tropomyosin 3, gamma
1447284_at	Mm.194294.1	2.39	0.017634	Trem1	triggering receptor expressed on myeloid cells 1
1427507_at	Mm.179733.1	-4.82	0.048587	Trim17	tripartite motif-containing 17
1441487_at	Mm.153117.1	-3.21	0.018364	Trim2	tripartite motif-containing 2
1446295_at	Mm.124121.1	-2.06	0.013558	Trim24	tripartite motif-containing 24
1453599_at	Mm.180272.1	-5.33	0.045388	Trim71	tripartite motif-containing 71
1443125_at	Mm.209265.1	-16.53	0.030105	Trip12	thyroid hormone receptor interactor 12
1459581_at	Mm.208871.1	-4.07	0.014117	Trp63	transformation related protein 63
1449431_at	Mm.8027.1	22.29	0.034009	Trpc6	transient receptor potential cation channel, subfamily C, member 6
1442174_at	Mm.46030.1	-2.13	0.003954	Tspan18	tetraspanin 18
1441877_x_at	Mm.181855.1	-3.03	0.039053	Tssc1	tumor suppressing subtransferable candidate 1
1458963_at	Mm.123362.1	-6.38	0.035635	Ttc39b	tetratricopeptide repeat domain 39B
1442762_at	Mm.197455.1	-2.85	0.029817	Ttll10	tubulin tyrosine ligase-like family, member 10
1454608_x_at	Mm.2108.3	-5.22	0.001244	Ttr	transthyretin
1452571_at	Mm.195098.1	-2.48	0.001534	Tuba-rs1	tubulin alpha, related sequence 1
1456031_at	Mm.21840.4	-11.03	0.007171	Tubb4b	tubulin, beta 4B class IVB
1417276_at	Mm.133800.1	5.16	0.008741	Tulp2	tubby-like protein 2
1425785_a_at	Mm.3264.2	-3.26	0.046339	Txk	TXK tyrosine kinase
1425249_a_at	Mm.2901.3	-3.9	0.042551	Tyro3	TYRO3 protein tyrosine kinase 3
1416745_x_at	Mm.27969.1	-2.41	0.011276	Uap1	UDP-N-acetylglucosamine pyrophosphorylase 1
1450285_at	Mm.89974.1	-5.68	0.014264	Uba1y	ubiquitin-activating enzyme, Chr Y
1420392_at	Mm.55982.1	-2.81	0.026707	Ubl4b	ubiquitin-like 4B
1420658_at	Mm.6254.1	3.56	0.028047	Ucp3	uncoupling protein 3 (mitochondrial, proton carrier)
1429836_at	Mm.40497.1	-3.19	0.010713	Uggt2	UDP-glucose glycoprotein glucosyltransferase 2
1450133_at	Mm.26794.1	4.82	0.021563	Ugt2a3	UDP glucuronosyltransferase 2 family, polypeptide A3
1439624_at	Mm.100151.1	-7.83	0.000848	Ugt2b35	UDP glucuronosyltransferase 2 family, polypeptide B35
1419592_at	Mm.24430.1	-3.34	0.017776	Unc5c	unc-5 homolog C (C. elegans)
1429950_at	Mm.81831.1	-9.66	0.023304	Unc5cl	unc-5 homolog C (C. elegans)-like
1445777_at	Mm.125644.1	-9.12	0.017393	Upf2	UPF2 regulator of nonsense transcripts homolog (yeast)
1437227_at	Mm.96110.1	2.1	0.016579	Urm1	ubiquitin related modifier 1 homolog (S. cerevisiae)
1452442_at	Mm.52047.1	-2.36	0.001959	Usp13	ubiquitin specific peptidase 13 (isopeptidase T-3)
1423778_at	Mm.34721.1	3.17	0.02855	Usp20	ubiquitin specific peptidase 20
1451105_at	Mm.22856.1	5.16	0.004965	Vash2	vasohibin 2
1447586_at	Mm.132721.1	3.98	0.021432	Vcan	versican
1421717_at	Mm.160379.1	-10.88	0.000008	Vmn1r58	vomeronasal 1 receptor 58
1421716_at	Mm.160378.1	-2.4	0.028641	Vmn1r65	vomeronasal 1 receptor 65
1447845_s_at	Mm.95361.1	-4.25	0.043719	Vnn1	vanin 1
1421050_at	Mm.220847.1	2.73	0.033854	Vps25	vacuolar protein sorting 25 (yeast)
1458471_at	Mm.164251.1	-5.65	0.040786	Wac	WW domain containing adaptor with coiled-coil
1459762_x_at	Mm.94136.1	8.84	0.02842	Wdr12	WD repeat domain 12
1453693_at	Mm.219475.1	2.56	0.022029	Wdr20b	WD repeat domain 20b
1459664_at	Mm.99983.1	-8.31	0.008172	Wdr31	WD repeat domain 31
1443924_at	Mm.32899.1	-9.19	0.007451	Wnk3	WNK lysine deficient protein kinase 3
1422228_at	Mm.558.1	-4.83	0.000659	Wnt8a	wingless-related MMTV integration site 8A
1441316_at	Mm.208150.1	-10.93	0.000969	Wnt8b	wingless related MMTV integration site 8b
1420008_s_at	Mm.203700.1	5.01	0.023217	Wwc1	WW, C2 and coiled-coil domain containing 1
1437320_s_at	Mm.4049.2	-10.94	0.038589	Xpa	xeroderma pigmentosum, complementation group A
1446826_at	Mm.186527.1	6.7	0.005858	Xpo7	exportin 7
1456843_at	Mm.194855.1	-4.5	0.007026	Yes1	Yamaguchi sarcoma viral (v-yes) oncogene homolog 1
1421614_at	Mm.7984.1	5.79	0.011467	Zan	zonadhesin
1419874_x_at	Mm.34106.2	6.1	0.045234	Zbtb16	zinc finger and BTB domain containing 16
1447042_at	Mm.103136.1	-6.45	0.004184	Zbtb34	zinc finger and BTB domain containing 34
1441787_at	Mm.105083.1	6.3	0.012973	Zc3h12c	zinc finger CCCH type containing 12C
1419955_at	Mm.219491.1	-7.58	0.022936	Zfand3	zinc finger, AN1-type domain 3
1420649_at	Mm.4270.1	-2.1	0.016435	Zfhx3	zinc finger homeobox 3
1421454_at	Mm.103750.1	3.81	0.032034	Zfp108	zinc finger protein 108
1421711_at	Mm.103751.1	-9.88	0.007632	Zfp109	zinc finger protein 109
1441619_at	Mm.183579.1	-4.6	0.004532	Zfp14	zinc finger protein 14
1430664_at	Mm.139257.1	-2.53	0.034614	Zfp169	zinc finger protein 169
1435087_at	Mm.22616.1	-2.9	0.048926	Zfp362	zinc finger protein 362
1451034_at	Mm.28161.1	-5.71	0.03168	Zfp36l2	zinc finger protein 36, C3H type-like 2
1418865_at	Mm.14099.1	3.61	0.048158	Zfp385a	zinc finger protein 385A
1451816_at	Mm.76755.1	-5.16	0.048879	Zfp451	zinc finger protein 451
1423836_at	Mm.21082.1	-2.7	0.036996	Zfp503	zinc finger protein 503
1423835_at	Mm.21082.1	-6.35	0.02487	Zfp503	zinc finger protein 503
1454555_at	Mm.159370.1	-4.77	0.004877	Zfp572	zinc finger protein 572
1439542_at	Mm.30269.1	3.39	0.005521	Zfp651	zinc finger protein 651
1442770_at	Mm.120791.1	4.75	0.014474	Zfp652	zinc finger protein 652
1445274_at	Mm.39257.1	-7.37	0.000043	Zfp781	zinc finger protein 781
1437925_at	Mm.3254.2	-5.08	0.020048	Zfp787	zinc finger protein 787
1443931_at	Mm.218258.1	-5.9	0.032843	Zfp882	zinc finger protein 882
1428033_at	Mm.66250.1	6.23	0.016284	Zfp937	zinc finger protein 937

1423424_at	Mm.4265.1	-3.68	0.036814	Zic3	zinc finger protein of the cerebellum 3
1425666_at	Mm.157721.1	-10.08	0.047294	Zic5	zinc finger protein of the cerebellum 5
1439077_at	Mm.130733.1	-2.27	0.000187	Zxda	zinc finger, X-linked, duplicated A
