# Supplementary material for: The miR-23a~27a~24-2 microRNA cluster buffers transcription and signaling pathways during hematopoiesis
Source: PLoS Genet. 2017 Jul 13;13(7):e1006887. doi: 10.1371/journal.pgen.1006887 (PMC5531666; doi:10.1371/journal.pgen.1006887)
Supplement: S3 Table — Two unique MiR-24 overexpressing 70Z/3 cell lines were generated through limiting dilution along with a control line infected with empty retrovirus. Cell lines were analyzed for genome wide RNA expression by microarray analysis using Affymetrix Mouse Genome 430 2.0 Arrays. Genes differentially regulated >2 fold between control and miR-24 overexpressing cell lines are shown. (PDF) [file pgen.1006887.s003.pdf]

Supplementary Table 3. Genes significantly changed in miR-27a overexpressing 70Z/3 Pre B Cells

Transcript Cluster ID		Fold Change	ANOVA p-value	Gene Symbol	Description
1418004_a_at	Mm.28385.1	21.56	0.000025	Tmem176b	transmembrane protein 176B
1427455_x_at	Mm.104747.15	15.22	0.000129	Igk-V28; Igkc; Igkj1;	immunoglobulin kappa chain variable 28 (V28); immunoglobulin kappa constantkappa chain variable
1423909_at	Mm.27061.1	10.39	0.00018	Tmem176a	transmembrane protein 176A
1452417_x_at	Mm.104747.14	9.48	0.000303	Igk-V28; Igkc; Igkj1;	immunoglobulin kappa chain variable 28 (V28); immunoglobulin kappa constantkappa chain variable
1427660_x_at	Mm.104747.19	9.2	0.000088	Igk-V28; Igkc; Igkj1;	immunoglobulin kappa chain variable 28 (V28); immunoglobulin kappa constantkappa chain variable
1431609_a_at	Mm.46354.4	8.58	0.049738	Acp5	acid phosphatase 5, tartrate resistant
1441811_x_at	Mm.200272.1	8.25	0.000035	Tmem176a	transmembrane protein 176A
1452463_x_at	Mm.104747.17	7.51	0.000186	Igkv8-30	immunoglobulin kappa chain variable 8-30
1450699_at	Mm.636.1	7.4	0.000349	Selenbp1	selenium binding protein 1
1425603_at	Mm.153276.1	7.28	0.000379	Tmem176a	transmembrane protein 176A
1450883_a_at	Mm.18628.1	6.47	0.026933	**Cd36**	**CD36 antigen**
1451446_at	Mm.29636.1	6.11	0.014043	Antxr1	anthrax toxin receptor 1
1448471_a_at	Mm.30144.1	5.89	0.000108	Ctla2a	cytotoxic T lymphocyte-associated protein 2 alpha
1428444_at	Mm.27159.1	5.67	0.000939	**Asb2**	**ankyrin repeat and SOCS box-containing 2**
1417580_s_at	Mm.196558.1	5.62	0.001009	Selenbp1	selenium binding protein 1
1456250_x_at	Mm.14455.3	4.75	0.005076	Tgfbi	transforming growth factor, beta induced
1416811_s_at	Mm.30144.1	4.73	0.000143	Ctla2a; Ctla2b	cytotoxic T lymphocyte-associated protein 2 alpha; cytotoxic T lymphocyte-associated protein 2 beta
1428391_at	Mm.24563.1	4.43	0.017217	Rab3il1	RAB3A interacting protein (rabin3)-like 1
1452217_at	Mm.203866.1	4.17	0.000916	Ahnak	AHNAK nucleoprotein (desmoyokin)
1433678_at	Mm.203915.1	4.11	0.000208	Pld4	phospholipase D family, member 4
1453304_s_at	Mm.204648.1	4.06	0.004633	**Ly6e**	**lymphocyte antigen 6 complex, locus E**
1421182_at	Mm.30700.1	4.02	0.026458	Clec1b	C-type lectin domain family 1, member b
1424542_at	Mm.3925.1	3.93	0.021203	S100a4	S100 calcium binding protein A4
1456741_s_at	Mm.15571.8	3.91	0.030044	Gpm6a	glycoprotein m6a
1416589_at	Mm.35439.1	3.82	0.019812	Sparc	secreted acidic cysteine rich glycoprotein
1436996_x_at	Mm.45436.2	3.8	0.011241	**Lyz1**	**lysozyme 1**
1423547_at	Mm.45436.1	3.71	0.011714	**Lyz2**	**lysozyme 2**
1448005_at	Mm.24385.1	3.57	0.00032	Sash1	SAM and SH3 domain containing 1
1424631_a_at	Mm.14438.1	3.34	0.041594	Ighg	Immunoglobulin heavy chain (gamma polypeptide)
1427713_x_at	Mm.37811.3	3.34	0.009927	**Pou2f2**	**POU domain, class 2, transcription factor 2**
1439426_x_at	Mm.45436.3	3.31	0.007669	**Lyz1**	**lysozyme 1**
1416521_at	Mm.42829.1	3.24	0.039587	Sepw1	selenoprotein W, muscle 1
1424680_at	Mm.66334.1	3.08	0.003613	Fam26e	family with sequence similarity 26, member E
1428284_at	Mm.60166.1	3.06	0.047149	8430427H17Rik	RIKEN cDNA 8430427H17 gene
1433942_at	Mm.215129.1	3.05	0.001413	Myo6	myosin VI
1434109_at	Mm.24472.1	3.05	0.001927	Sh3bgrl2	SH3 domain binding glutamic acid-rich protein like 2
1426442_at	Mm.178672.1	3	0.000532	Gpm6a	glycoprotein m6a
1422537_a_at	Mm.1466.1	2.97	0.044157	**Id2**	**inhibitor of DNA binding 2**
1426388_s_at	Mm.3860.3	2.97	0.009445	Ryk	receptor-like tyrosine kinase
1431648_at	Mm.52592.1	2.95	0.002481	Meiob	meiosis specific with OB domains
1448123_s_at	Mm.14455.1	2.91	0.009713	Tgfbi	transforming growth factor, beta induced
1460585_x_at	Mm.220974.2	2.9	0.000272	Pisd	phosphatidylserine decarboxylase
1457270_at	Mm.152121.1	2.89	0.031301	Gas7	growth arrest specific 7
1417149_at	Mm.3705.1	2.87	0.004215	P4ha2	procollagen-proline, 2-oxoglutarate 4-dioxygenase (proline 4-hydroxylase), alpha II polypeptide
1456616_a_at	Mm.726.3	2.87	0.003938	Bsg	basigin
1451344_at	Mm.41681.1	2.86	0.042915	Tmem119	transmembrane protein 119
1428509_at	Mm.100720.1	2.76	0.009424	Myo1e	myosin IE
1417235_at	Mm.18526.1	2.75	0.018267	Ehd3	EH-domain containing 3
1418641_at	Mm.1781.1	2.73	0.002335	**Lcp2**	**lymphocyte cytosolic protein 2 (slp76)**
1422906_at	Mm.196728.1	2.71	0.001559	Abcg2	ATP-binding cassette, sub-family G (WHITE), member 2
1416239_at	Mm.3217.1	2.65	0.032563	Ass1	argininosuccinate synthetase 1
1418128_at	Mm.157091.1	2.58	0.0023	Adcy6	adenylate cyclase 6
1455203_at	Mm.35159.1	2.57	0.002273	A930003A15Rik	RIKEN cDNA A930003A15 gene
1416767_a_at	Mm.27647.1	2.57	0.018613	Smim14	small integral membrane protein 14
1425133_s_at	Mm.200929.1	2.56	0.015174	Rab3il1	RAB3A interacting protein (rabin3)-like 1
1423802_at	Mm.214549.1	2.55	0.001668	Camkv	CaM kinase-like vesicle-associated
1450545_a_at	Mm.25620.1	2.55	0.041359	Dntt	deoxynucleotidyltransferase, terminal
1420150_at	Mm.219560.1	2.54	0.000146	Spsb1	splA/ryanodine receptor domain and SOCS box containing 1
1417756_a_at	Mm.2183.1	2.54	0.005289	**Lsp1**	**lymphocyte specific 1**
1415922_s_at	Mm.2769.1	2.5	0.049132	Marcksl1	MARCKS-like 1
1455925_at	Mm.63232.1	2.46	0.032317	Prdm8	PR domain containing 8
1449757_x_at	Mm.204633.1	2.45	0.017619	Dntt	deoxynucleotidyltransferase, terminal
1438540_at	Mm.91728.1	2.45	0.002505	Col25a1	collagen, type XXV, alpha 1
1419331_at	Mm.33402.1	2.38	0.00899	**Cdh17**	**cadherin 17**
1436448_a_at	Mm.2792.2	2.36	0.009268	Ptgs1	prostaglandin-endoperoxide synthase 1
1429681_a_at	Mm.142511.2	2.32	0.027821	Tecr	trans-2,3-enoyl-CoA reductase
1421255_a_at	Mm.38551.1	2.31	0.003862	Cabp1	calcium binding protein 1
1422580_at	Mm.43.1	2.31	0.013001	Myl4	myosin, light polypeptide 4
1455265_a_at	Mm.181709.3	2.31	0.026643	Rgs16	regulator of G-protein signaling 16
1455451_at	Mm.22056.1	2.27	0.034012	Kctd14	potassium channel tetramerisation domain containing 14
1426260_a_at	Mm.42472.3	2.26	0.014711	LOC100048662; LO	UDP-glucuronosyltransferase 1-9-like; UDP-glucuronosyltransferase 1-8-like; polypeptide A10;
1451527_at	Mm.46016.1	2.25	0.00046	Pcolce2	procollagen C-endopeptidase enhancer 2
1430700_a_at	Mm.9277.2	2.25	0.006273	Pla2g7	phospholipase A2, group VII (platelet-activating factor acetylhydrolase, plasma)
1423414_at	Mm.2792.1	2.24	0.004993	Ptgs1	prostaglandin-endoperoxide synthase 1
1455215_at	Mm.39722.1	2.24	0.005865	Pianp	PILR alpha associated neural protein
1426538_a_at	Mm.222.7	2.22	0.042617	**Trp53**	**transformation related protein 53**
1415973_at	Mm.30059.1	2.2	0.0388	Marcks	myristoylated alanine rich protein kinase C substrate
1437217_at	Mm.135084.1	2.19	0.001528	Ankrd6	ankyrin repeat domain 6
1419219_at	Mm.137441.1	2.19	0.000913	Cyp4f18	cytochrome P450, family 4, subfamily f, polypeptide 18
1449925_at	Mm.12876.1	2.18	0.000839	Cxcr3	chemokine (C-X-C motif) receptor 3
1418448_at	Mm.257.1	2.18	0.000489	Rras	Harvey rat sarcoma oncogene, subgroup R
1425531_at	Mm.41486.1	2.18	0.049945	Znhit1	zinc finger, HIT domain containing 1
1426201_at	Mm.220163.1	2.18	0.000131	Igkv4-72	immunoglobulin kappa chain variable 4-72
1426261_s_at	Mm.42472.3	2.18	0.024631	LOC100048662; LO	UDP-glucuronosyltransferase 1-9-like; UDP-glucuronosyltransferase 1-8-like
1449380_at	Mm.4926.1	2.18	0.004419	Pacsin1	protein kinase C and casein kinase substrate in neurons 1
1437463_x_at	Mm.14455.2	2.15	0.025969	Tgfbi	transforming growth factor, beta induced
1448878_at	Mm.206736.1	2.15	0.010911	Mxd3	Max dimerization protein 3
1437226_x_at	Mm.2769.6	2.14	0.035655	Marcksl1	MARCKS-like 1
1449374_at	Mm.8543.1	2.12	0.020687	Pipox	pipecolic acid oxidase
1436230_at	Mm.30498.1	2.11	0.000739	Gpr114	G protein-coupled receptor 114
1439060_s_at	Mm.35817.4	2.1	0.000752	Wipi1	WD repeat domain, phosphoinositide interacting 1
1429893_at	Mm.27109.1	2.1	0.016618	Il17rd	interleukin 17 receptor D
1450183_a_at	Mm.200936.1	2.09	0.049791	Sh2b3	SH2B adaptor protein 3
1451950_a_at	Mm.89474.5	2.09	0.01192	Cd80	CD80 antigen
1451427_a_at	Mm.46628.2	2.08	0.015647	Egfl7	EGF-like domain 7
1440355_at	Mm.33018.1	2.07	0.001123	Kctd12b	potassium channel tetramerisation domain containing 12b
1416289_at	Mm.37371.1	2.06	0.045134	Plod1	procollagen-lysine, 2-oxoglutarate 5-dioxygenase 1
1424256_at	Mm.121647.1	2.06	0.029069	Rdh12	retinol dehydrogenase 12
1426063_a_at	Mm.4362.2	2.06	0.004625	Gem	GTP binding protein (gene overexpressed in skeletal muscle)

1433930_at	Mm.116888.1	2.06	0.020279	Hpse	heparanase
1424634_at	Mm.40713.1	2.05	0.011017	Tceal1	transcription elongation factor A (SII)-like 1
1418284_at	Mm.133919.1	2.04	0.03983	Vps72	vacuolar protein sorting 72 (yeast)
1424633_at	Mm.40329.1	2.04	0.015087	Camk1g	calcium/calmodulin-dependent protein kinase I gamma
1436216_s_at	Mm.22788.2	2.04	0.028121	Inf2	inverted formin, FH2 and WH2 domain containing
1422952_at	Mm.214512.1	2.03	0.027231	Sapcd1	suppressor APC domain containing 1
1439622_at	Mm.36661.1	2.03	0.003273	Rassf4	Ras association (RalGDS/AF-6) domain family member 4
1424783_a_at	Mm.42472.1	2.02	0.016848	LOC100048662; LO	UDP-glucuronosyltransferase 1-9-like;polypeptide A10; polypeptide A6A; UDP glucuronosyltrans
1455257_at	Mm.87150.1	2.02	0.032332	Itgb3	integrin beta 3
1429764_at	Mm.34131.1	2.01	0.006754	Fam101b	family with sequence similarity 101, member B
1451428_x_at	Mm.46628.2	2.01	0.031097	Egfl7	EGF-like domain 7
1417623_at	Mm.4168.1	-2.01	0.036113	Slc12a2	solute carrier family 12, member 2
1426542_at	Mm.41423.1	-2.01	0.008443	Endod1	endonuclease domain containing 1
1442103_at	Mm.103120.1	-2.01	0.012685	Nipbl	Nipped-B homolog (Drosophila)
1444004_at	Mm.25284.1	-2.01	0.049646	Thoc2	THO complex 2
1435342_at	Mm.24877.1	-2.02	0.021372	Kcnk6	potassium inwardly-rectifying channel, subfamily K, member 6
1422005_at	Mm.807.1	-2.03	0.01522	Eif2ak2	eukaryotic translation initiation factor 2-alpha kinase 2
1442656_at	Mm.154685.1	-2.03	0.033809	Elovl6	ELOVL family member 6, elongation of long chain fatty acids (yeast)
1417754_at	Mm.39006.1	-2.05	0.010254	Topors	topoisomerase I binding, arginine/serine-rich
1417961_a_at	Mm.3288.1	-2.05	0.00745	Trim30a	tripartite motif-containing 30A
1426371_at	Mm.147529.1	-2.05	0.034473	Far1	fatty acyl CoA reductase 1
1443330_at	Mm.103952.1	-2.06	0.016783	D1Ertd646e	DNA segment, Chr 1, ERATO Doi 646, expressed
1455980_a_at	Mm.11982.1	-2.06	0.007855	Gas2l3	growth arrest-specific 2 like 3
1416482_at	Mm.3679.1	-2.07	0.020528	Ttc3	tetratricopeptide repeat domain 3
1431686_a_at	Mm.87312.3	-2.07	0.015628	Gmfb	glia maturation factor, beta
1452115_a_at	Mm.3794.2	-2.07	0.023382	Plk4	polo-like kinase 4
1441548_at	Mm.139174.1	-2.07	0.041021	Frmd4b	FERM domain containing 4B
1456257_at	Mm.26272.1	-2.07	0.010109	Fam126b	family with sequence similarity 126, member B
1450276_a_at	Mm.2416.1	-2.08	0.000235	Scin	scinderin
1445824_at	Mm.61078.1	-2.08	0.002335	Zfp458	zinc finger protein 458
1426914_at	Mm.25300.1	-2.09	0.000148	Marveld2	MARVEL (membrane-associating) domain containing 2
1425687_at	Mm.1993.2	-2.1	0.010526	Cflar	CASP8 and FADD-like apoptosis regulator
1451828_a_at	Mm.143689.2	-2.1	0.000565	Acsl4	acyl-CoA synthetase long-chain family member 4
1452187_at	Mm.46706.1	-2.1	0.031029	Rbm5	RNA binding motif protein 5
1455388_at	Mm.21539.1	-2.1	0.03427	Pcmtd1	protein-L-isoaspartate (D-aspartate) O-methyltransferase domain containing 1
1452741_s_at	Mm.41718.1	-2.1	0.017271	Gpd2	glycerol phosphate dehydrogenase 2, mitochondrial
1455251_at	Mm.87506.1	-2.11	0.011512	Itga1	integrin alpha 1
1430047_at	Mm.44754.1	-2.12	0.045832	Ankrd32	ankyrin repeat domain 32
1446929_at	Mm.215982.1	-2.12	0.016286	D130062J21Rik	RIKEN cDNA D130062J21 gene
1415823_at	Mm.193096.1	-2.13	0.011168	Scd2	stearoyl-Coenzyme A desaturase 2
1454823_at	Mm.30489.1	-2.14	0.034419	Wdr37	WD repeat domain 37
1425014_at	Mm.3535.1	-2.15	0.002435	Nr2c2	nuclear receptor subfamily 2, group C, member 2
1427682_a_at	Mm.1353.2	-2.15	0.001595	Egr2	early growth response 2
1417069_a_at	Mm.87312.1	-2.16	0.011798	Gmfb	glia maturation factor, beta
1417783_at	Mm.30114.1	-2.16	0.022229	Als2	amyotrophic lateral sclerosis 2 (juvenile)
1422018_at	Mm.42157.1	-2.16	0.01013	Hivep2	human immunodeficiency virus type I enhancer binding protein 2
1456494_a_at	Mm.3288.3	-2.16	0.00241	Trim30a; Trim30d	tripartite motif-containing 30A; tripartite motif-containing 30D
1418027_at	Mm.34988.1	-2.17	0.037645	Exo1	exonuclease 1
1421299_a_at	Mm.200634.1	-2.17	0.012411	**Lef1**	**lymphoid enhancer binding factor 1**
1449292_at	Mm.141567.1	-2.17	0.025747	Rb1cc1	RB1-inducible coiled-coil 1
1429327_at	Mm.46705.1	-2.17	0.012707	Nemf	nuclear export mediator factor
1429633_at	Mm.30876.1	-2.17	0.032574	Lcor	ligand dependent nuclear receptor corepressor
1419480_at	Mm.1461.1	-2.18	0.00992	Sell	selectin, lymphocyte
1423162_s_at	Mm.39505.1	-2.18	0.023145	Spred1	sprouty protein with EVH-1 domain 1, related sequence
1417065_at	Mm.181959.1	-2.19	0.021514	**Egr1**	**early growth response 1**
1437883_s_at	Mm.79960.2	-2.19	0.034604	Pan3	PAN3 polyA specific ribonuclease subunit homolog (S. cerevisiae)
1444320_at	Mm.35520.1	-2.19	0.022202	Ddhd2	DDHD domain containing 2
1415824_at	Mm.193096.1	-2.2	0.00436	Scd2	stearoyl-Coenzyme A desaturase 2
1435363_at	Mm.11768.1	-2.2	0.000119	Plekhg1	pleckstrin homology domain containing, family G (with RhoGef domain) member 1
1423546_at	Mm.12236.1	-2.21	0.035044	Zfp207	zinc finger protein 207
1431096_at	Mm.158856.1	-2.21	0.042979	Ints8	integrator complex subunit 8
1437917_at	Mm.44063.1	-2.23	0.01635	D530037H12Rik	RIKEN cDNA D530037H12 gene
1418023_at	Mm.28256.1	-2.24	0.00189	Naa15	N(alpha)-acetyltransferase 15, NatA auxiliary subunit
1436746_at	Mm.27341.1	-2.24	0.033779	Wnk1	WNK lysine deficient protein kinase 1
1438719_at	Mm.100184.1	-2.25	0.018351	Map3k2	mitogen-activated protein kinase kinase kinase 2
1440729_at	Mm.83041.1	-2.25	0.022503	Eps15	epidermal growth factor receptor pathway substrate 15
1427683_at	Mm.1353.2	-2.26	0.00253	**Egr2**	**early growth response 2**
1432686_at	Mm.159245.1	-2.27	0.031573	4833406M21Rik	RIKEN cDNA 4833406M21 gene
1439158_at	Mm.132569.1	-2.27	0.002092	Tlk1	tousled-like kinase 1
1443314_at	Mm.166033.1	-2.28	0.042722	Katnbl1	katanin p80 subunit B like 1
1452360_a_at	Mm.95879.2	-2.3	0.032302	**Kdm5a**	**lysine (K)-specific demethylase 5A**
1459923_at	Mm.24058.1	-2.31	0.004499	Bex6	brain expressed gene 6
1416190_a_at	Mm.28375.1	-2.31	0.003504	Sec61a1	Sec61 alpha 1 subunit (S. cerevisiae)
1425686_at	Mm.1993.2	-2.31	0.016969	Cflar	CASP8 and FADD-like apoptosis regulator
1436311_at	Mm.249.1	-2.31	0.025206	Gemin5	gem (nuclear organelle) associated protein 5
1419277_at	Mm.218478.1	-2.32	0.001431	Usp48	ubiquitin specific peptidase 48
1449615_s_at	Mm.215181.1	-2.32	0.04915	Hdlbp	high density lipoprotein (HDL) binding protein
1430996_at	Mm.196190.1	-2.32	0.006192	Etnk1	ethanolamine kinase 1
1445198_at	Mm.131877.1	-2.33	0.020593	Kdm6a	lysine (K)-specific demethylase 6A
1459137_at	Mm.138861.1	-2.34	0.022124	Pml	promyelocytic leukemia
1453988_a_at	Mm.28366.2	-2.37	0.004986	Ide	insulin degrading enzyme
1425923_at	Mm.16469.2	-2.38	0.026293	Mycn	v-myc myelocytomatosis viral related oncogene, neuroblastoma derived (avian)
1437158_at	Mm.29384.2	-2.39	0.004927	Nipbl	Nipped-B homolog (Drosophila)
1439180_at	Mm.31924.1	-2.39	0.009148	Ino80d	INO80 complex subunit D
1417755_at	Mm.39006.1	-2.41	0.00155	Topors	topoisomerase I binding, arginine/serine-rich
1424325_at	Mm.21177.1	-2.44	0.032687	Esco1	establishment of cohesion 1 homolog 1 (S. cerevisiae)
1424598_at	Mm.157743.1	-2.45	0.01781	Ddx6	DEAD (Asp-Glu-Ala-Asp) box polypeptide 6
1435163_at	Mm.27433.1	-2.45	0.006844	Zfp871	zinc finger protein 871
1421186_at	Mm.6272.1	-2.46	0.007203	Ccr2	chemokine (C-C motif) receptor 2
1430618_at	Mm.90014.1	-2.46	0.039257	2610020C07Rik	RIKEN cDNA 2610020C07 gene
1439706_at	Mm.185024.1	-2.46	0.025494	A330106F07Rik	RIKEN cDNA A330106F07 gene
1447649_x_at	Mm.70400.1	-2.46	0.004087	Dnajc1	DnaJ (Hsp40) homolog, subfamily C, member 1
1427319_at	Mm.100634.1	-2.49	0.023604	A230046K03Rik	RIKEN cDNA A230046K03 gene
1422160_at	Mm.14573.1	-2.51	0.001887	H2-T24	histocompatibility 2, T region locus 24
1434357_a_at	Mm.16710.3	-2.51	0.020827	Kpnb1	karyopherin (importin) beta 1
1433242_at	Mm.159649.1	-2.51	0.001256	5830415B17Rik	RIKEN cDNA 5830415B17 gene
1458924_at	Mm.134930.1	-2.51	0.046734	D430013B06Rik	RIKEN cDNA D430013B06 gene
1443522_s_at	Mm.132762.1	-2.52	0.026371	Phip	pleckstrin homology domain interacting protein
1440104_at	Mm.193920.1	-2.55	0.046102	Ranbp2	RAN binding protein 2
1437719_x_at	Mm.100634.2	-2.56	0.038062	A230046K03Rik	RIKEN cDNA A230046K03 gene

1460729_at	Mm.6710.1	-2.56	0.036769	Rock1	Rho-associated coiled-coil containing protein kinase 1
1441907_s_at	Mm.138523.1	-2.56	0.011248	**Cd93**	**CD93 antigen**
1417784_at	Mm.30114.1	-2.57	0.003494	Als2	amyotrophic lateral sclerosis 2 (juvenile)
1450051_at	Mm.10141.1	-2.57	0.00207	Atrx	alpha thalassemia/mental retardation syndrome X-linked homolog (human)
1442277_at	Mm.100007.1	-2.57	0.042459	Chka	choline kinase alpha
1442312_at	Mm.214658.1	-2.57	0.009646	Tbl1xr1	transducin (beta)-like 1X-linked receptor 1
1446118_at	Mm.154700.1	-2.57	0.0414	D17Ertd165e	DNA segment, Chr 17, ERATO Doi 165, expressed
1433589_at	Mm.28524.1	-2.59	0.004171	Fam21	family with sequence similarity 21
1425581_s_at	Mm.221162.1	-2.63	0.005691	Galnt7	UDP-N-acetyl-alpha-D-galactosamine: polypeptide N-acetylgalactosaminyltransferase 7
1437221_at	Mm.115282.1	-2.64	0.018164	Rrm2b	ribonucleotide reductase M2 B (TP53 inducible)
1437222_x_at	Mm.115282.1	-2.64	0.026167	Rrm2b	ribonucleotide reductase M2 B (TP53 inducible)
1441238_at	Mm.103018.1	-2.65	0.033231	Pds5a	PDS5, regulator of cohesion maintenance, homolog A (S. cerevisiae)
1457186_at	Mm.131416.1	-2.65	0.003415	Tbl1xr1	transducin (beta)-like 1X-linked receptor 1
1440315_at	Mm.132809.1	-2.66	0.006798	Mbnl1	muscleblind-like 1 (Drosophila)
1445881_at	Mm.176095.1	-2.67	0.010863	2310035P21Rik	RIKEN cDNA 2310035P21 gene
1418126_at	Mm.3370.1	-2.69	0.042901	Ccl5	chemokine (C-C motif) ligand 5
1422748_at	Mm.37676.1	-2.69	0.031341	Zeb2	zinc finger E-box binding homeobox 2
1418230_a_at	Mm.29097.1	-2.71	0.03831	Lims1	LIM and senescent cell antigen-like domains 1
1451793_at	Mm.25227.1	-2.71	0.034829	Klhl24	kelch-like 24
1445068_at	Mm.214913.1	-2.72	0.022882	Malt1	mucosa associated lymphoid tissue lymphoma translocation gene 1
1447984_at	Mm.34935.1	-2.74	0.00831	D1Ertd75e	DNA segment, Chr 1, ERATO Doi 75, expressed
1442513_at	Mm.123943.1	-2.74	0.031357	BC016423	cDNA sequence BC016423
1439450_x_at	Mm.100634.3	-2.77	0.02563	A230046K03Rik	RIKEN cDNA A230046K03 gene
1441988_at	Mm.216238.1	-2.77	0.006273	Ppm1k	protein phosphatase 1K (PP2C domain containing)
1454299_at	Mm.206828.1	-2.77	0.021277	4833422B07Rik	RIKEN cDNA 4833422B07 gene
1417792_at	Mm.4503.1	-2.8	0.018184	Zfml	zinc finger, matrin-like
1421008_at	Mm.24045.1	-2.81	0.037554	Rsad2	radical S-adenosyl methionine domain containing 2
1426541_a_at	Mm.41423.1	-2.81	0.018929	Endod1	endonuclease domain containing 1
1437118_at	Mm.132701.1	-2.83	0.001648	Usp7	ubiquitin specific peptidase 7
1456382_at	Mm.46236.1	-2.84	0.032766	Atad1	ATPase family, AAA domain containing 1
1457731_at	Mm.128872.1	-2.84	0.033409	Snapc3	small nuclear RNA activating complex, polypeptide 3
1459791_at	Mm.70400.1	-2.86	0.002651	Dnajc1	DnaJ (Hsp40) homolog, subfamily C, member 1
1442489_at	Mm.155160.1	-2.92	0.026129	D1Ertd564e	DNA segment, Chr 1, ERATO Doi 564, expressed
1455682_at	Mm.82640.1	-2.92	0.009179	Abl2	v-abl Abelson murine leukemia viral oncogene 2 (arg, Abelson-related gene)
1427311_at	Mm.26671.1	-2.93	0.048597	Bptf	bromodomain PHD finger transcription factor
1433598_at	Mm.27476.1	-2.97	0.02979	Arglu1	arginine and glutamate rich 1
1451569_at	Mm.3535.1	-3	0.005088	Nr2c2	nuclear receptor subfamily 2, group C, member 2
1423597_at	Mm.33083.1	-3.05	0.000367	Atp8a1	ATPase, aminophospholipid transporter (APLT), class I, type 8A, member 1
1421187_at	Mm.6272.1	-3.08	0.014781	Ccr2	chemokine (C-C motif) receptor 2
1440417_at	Mm.122725.1	-3.21	0.01637	D19Ertd409e	DNA segment, Chr 19, ERATO Doi 409, expressed
1416661_at	Mm.2238.1	-3.29	0.040535	Eif3a	eukaryotic translation initiation factor 3, subunit A
1417831_at	Mm.26412.1	-3.29	0.017545	Smc1a	structural maintenance of chromosomes 1A
1456498_at	Mm.122709.2	-3.32	0.000186	**Itga4**	**integrin alpha 4**
1418191_at	Mm.27498.1	-3.39	0.039387	Usp18	ubiquitin specific peptidase 18
1457493_at	Mm.196766.1	-3.39	0.003903	Pten	phosphatase and tensin homolog
1453512_at	Mm.167748.1	-3.53	0.025494	5830407P18Rik	RIKEN cDNA 5830407P18 gene
1426259_at	Mm.29566.1	-3.73	0.014164	Pank3	pantothenate kinase 3
1455016_at	Mm.30550.1	-3.73	0.041514	Prpf38b	PRP38 pre-mRNA processing factor 38 (yeast) domain containing B
1440924_at	Mm.49800.1	-3.83	0.026532	Kif20b	kinesin family member 20B
1438519_at	Mm.63354.1	-4.36	0.00763	Klhl24	kelch-like 24
1438515_at	Mm.169614.1	-4.48	0.007198	Zfp207	zinc finger protein 207
1437322_at	Mm.66278.1	-4.6	0.021771	Rbm4	RNA binding motif protein 4
1437581_at	Mm.132591.1	-6.29	0.008802	Zfp800	zinc finger protein 800
