# Supplementary figures and images for: The miR-23a~27a~24-2 microRNA cluster buffers transcription and signaling pathways during hematopoiesis
Source: PLoS Genet. 2017 Jul 13;13(7):e1006887. doi: 10.1371/journal.pgen.1006887 (PMC5531666; doi:10.1371/journal.pgen.1006887)

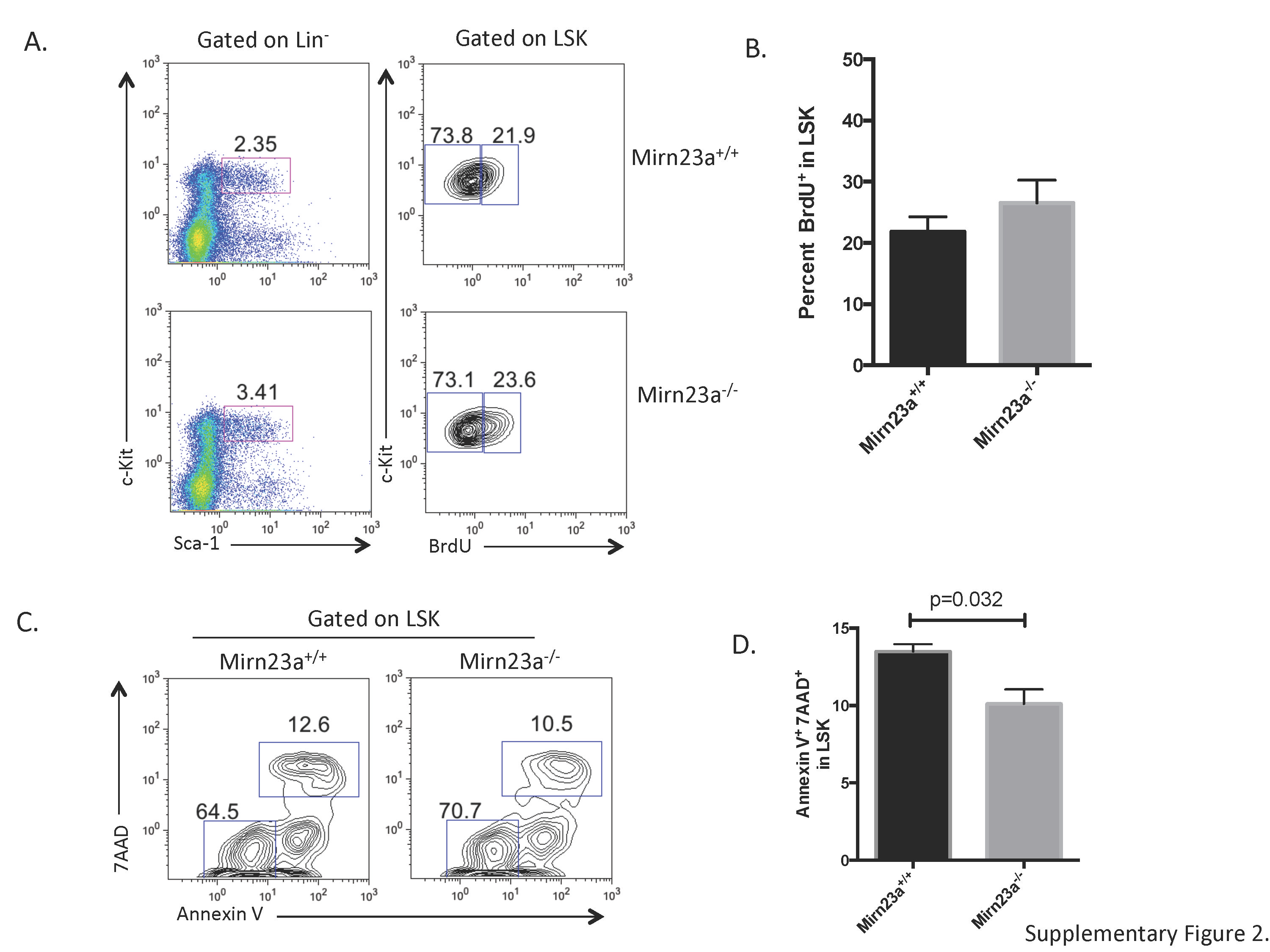

Supplement: S2 Fig — A) Wildtype and mirn23a-/- mice were injected intraperitoneally with BrdU 16 hours prior to sacrifice. Bone marrow was then harvested and cells were fixed and permeabilized then analyzed for LSK and BrdU expression by FACS. Representative plots are shown. B) No significant differences were observed between wildtype and mirn23a-/- mice. C) Bone marrow was harvested from wildtype and mirn23a-/- mice and stained for LSK surface markers along with annexin V and 7AAD. Representative plots are shown. D) A slight decrease was observed in mirn23a-/- mice that was statistically significant. Statistical analysis done by unpaired students t-test. (TIFF) [file pgen.1006887.s005.tiff]

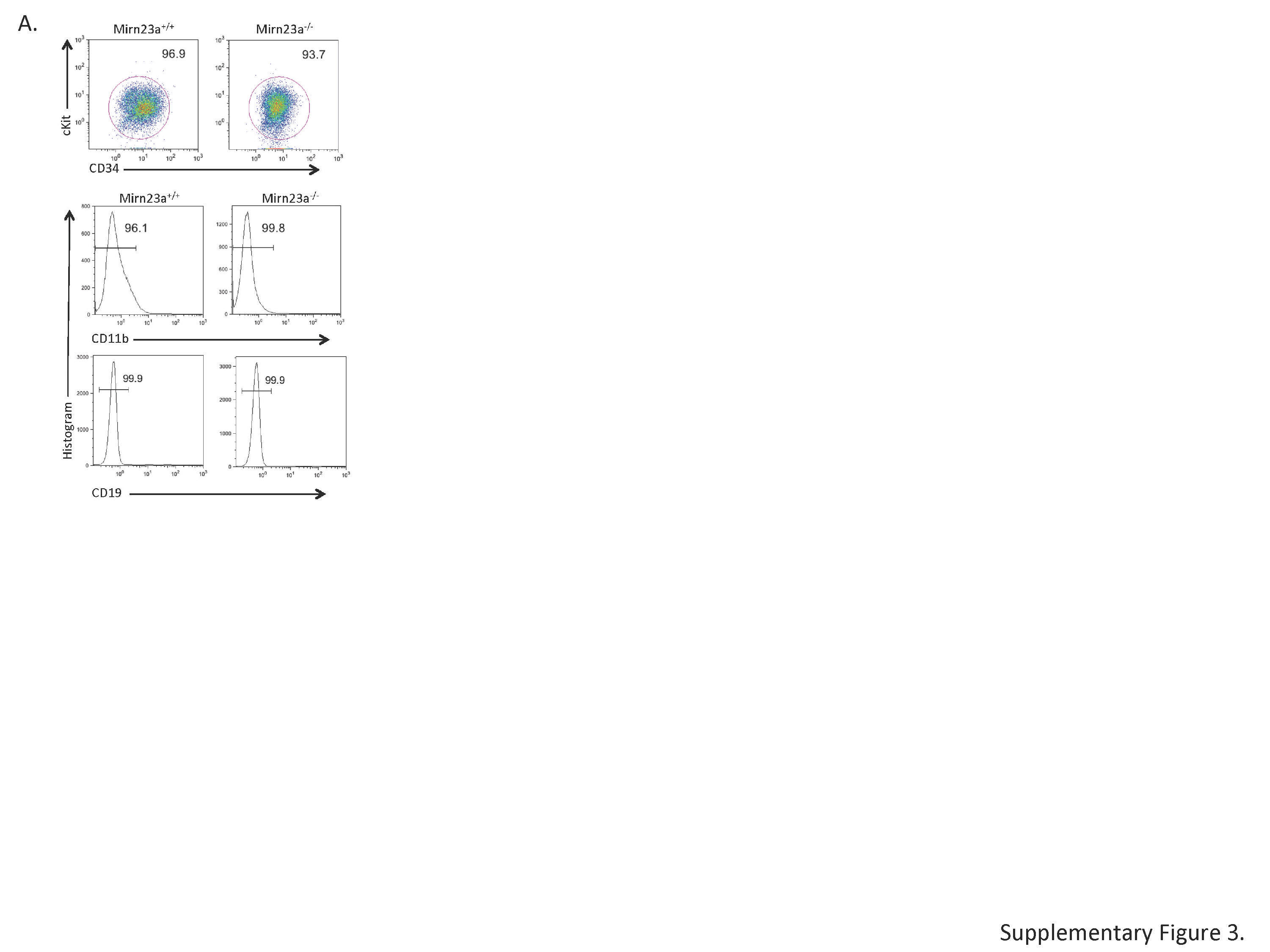

Supplement: S3 Fig — A) EML cells derived from wildtype and mirn23a-/- mice express stem cell markers and do not express any committed lineage markers. (TIFF) [file pgen.1006887.s006.tiff]

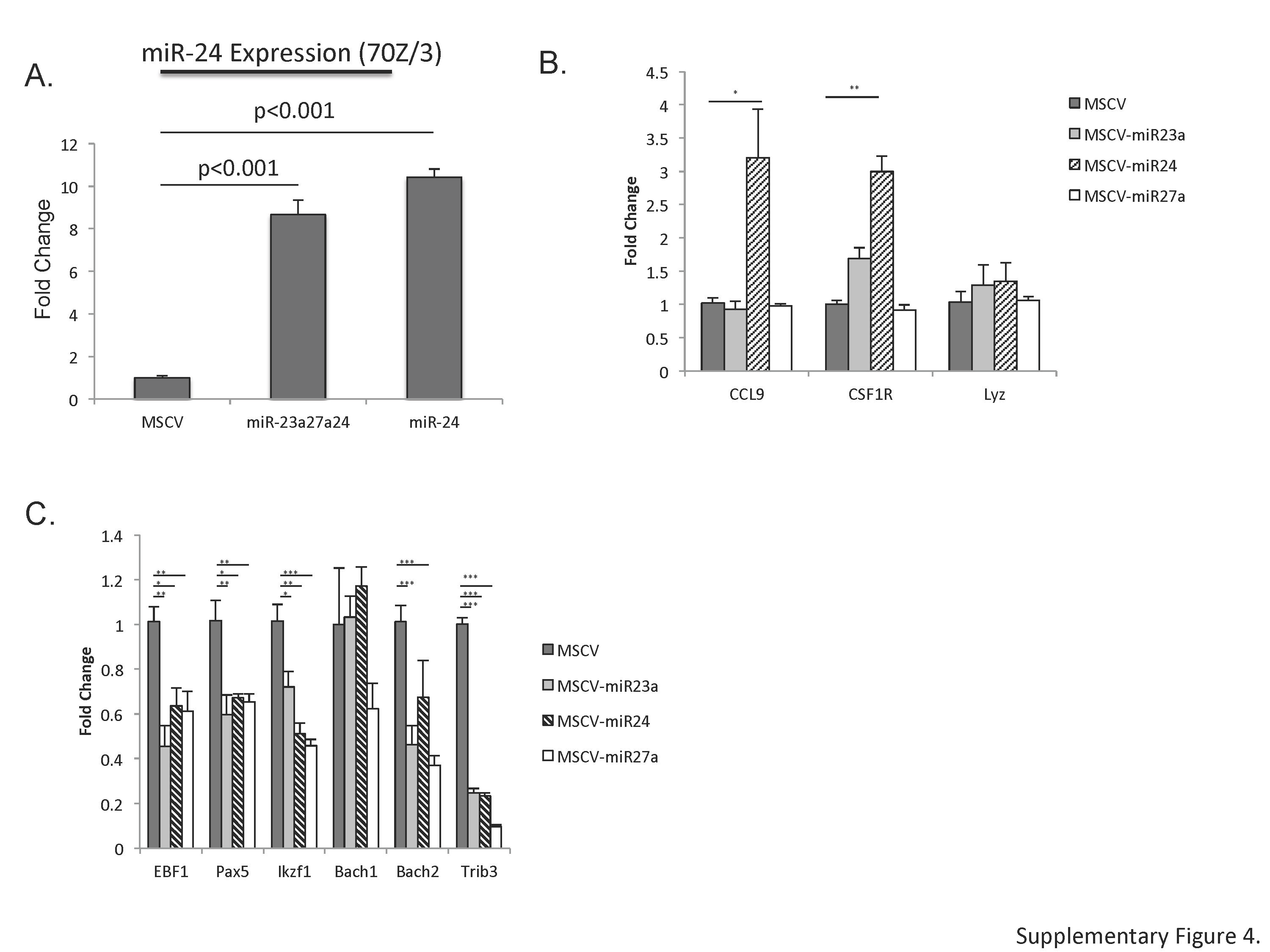

Supplement: S4 Fig — A) 70Z/3 pre B cells were transduced with control, mirn23a cluster, or miR-24 alone overexpression vectors. MiR-24 expression was analyzed by qRT-PCR to validate overexpression. B) The contribution of each individual miRNA to myeloid gene expression was evaluated by qRTPCR. MiR-24 had the ability to upregulate critical myeloid genes Ccl9 and Csf1r. C) The contribution of each individual miRNA to lymphoid gene expression was evaluated by qRTPCR. All three miRNAs had the ability to significantly downregulate lymphoid gene expression. Statistical analysis done by unpaired students t-test. (TIFF) [file pgen.1006887.s007.tiff]

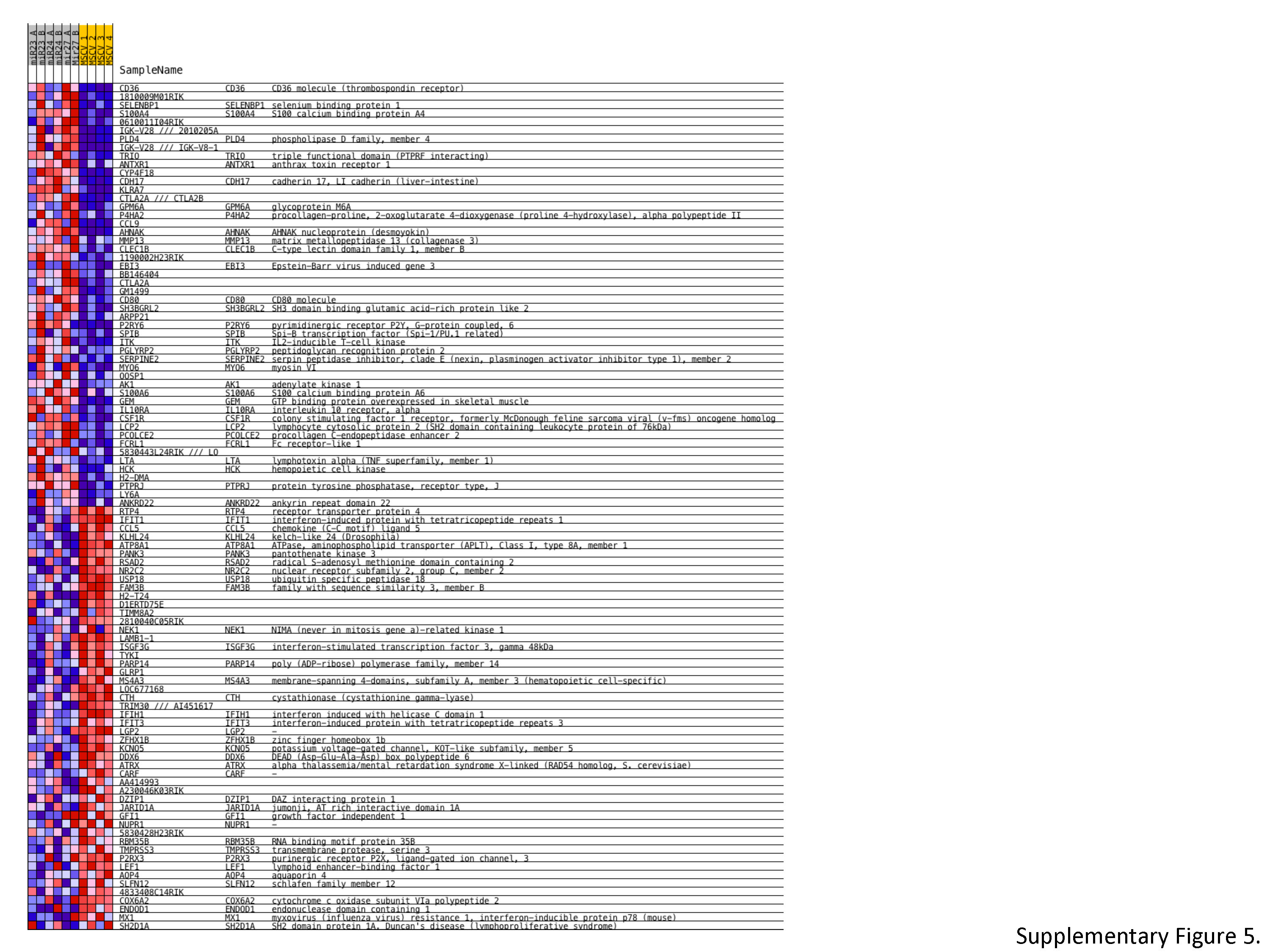

Supplement: S5 Fig — Two unique lines for each miRNA and a control line infected with empty retrovirus were generated in 70Z/3 cells and analyzed for genome wide RNA expression by microarray analysis using Affymetrix Mouse Genome 430 2.0 Arrays. A heat map of a ranked list of the top 50 upregulated and downregulated genes in the miRNA expressing 70Z/3 lines compared to the empty vector expressing lines is shown. (TIFF) [file pgen.1006887.s008.tiff]

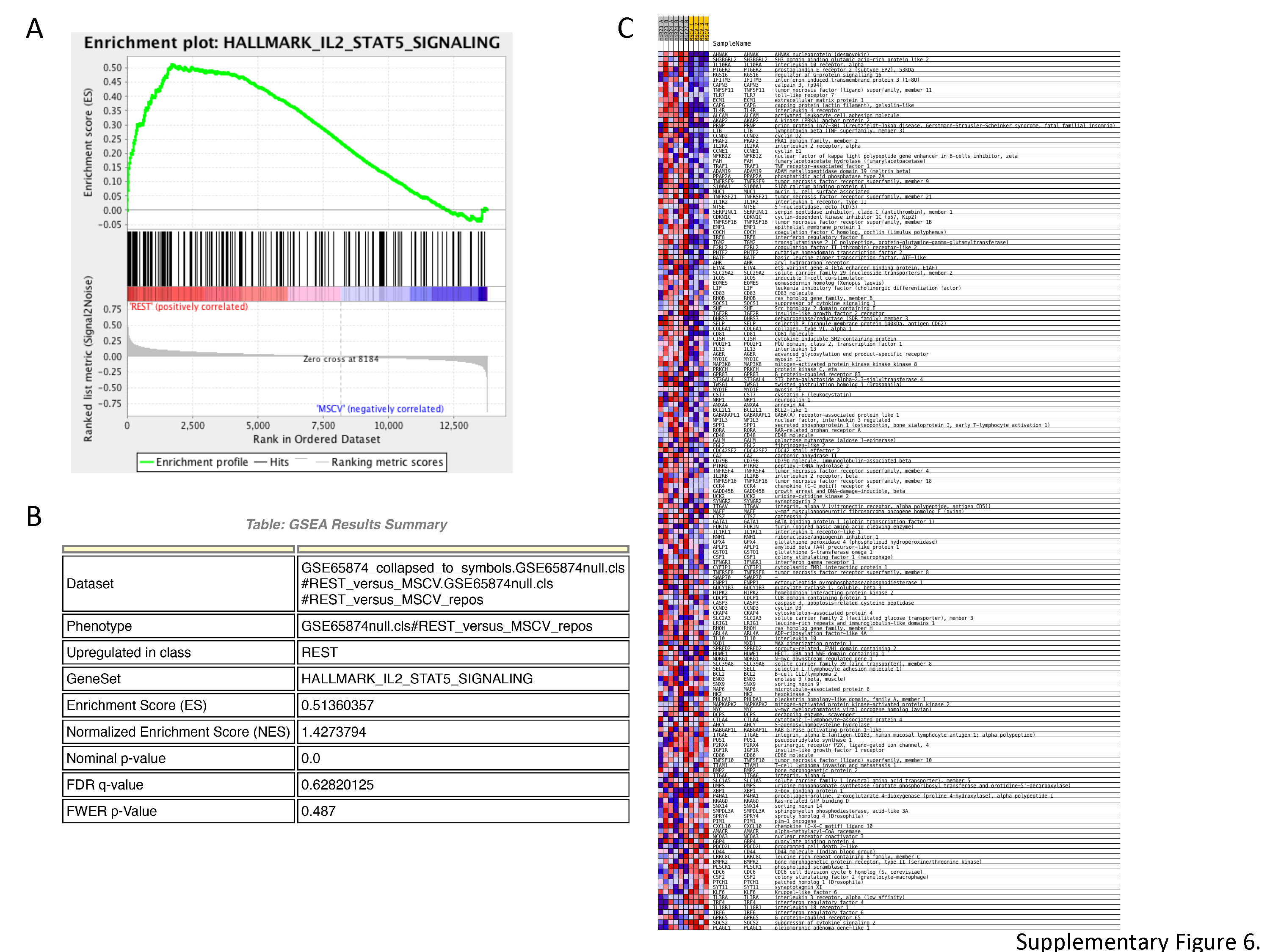

Supplement: S6 Fig — A) A gene set enrichment analysis was performed on the microarray data from 70Z/3 cells overexpressing individual components of the mirn23a cluster. The enrichment plot for IL2/Stat5 signaling is shown. B) Results summary for IL2/Stat5 signaling from the gene set enrichment analysis. C) Heat map showing the individual components of the IL2/Stat5 signaling pathways affected by miR-23a, miR-24, or miR-27a expression. (TIFF) [file pgen.1006887.s009.tiff]

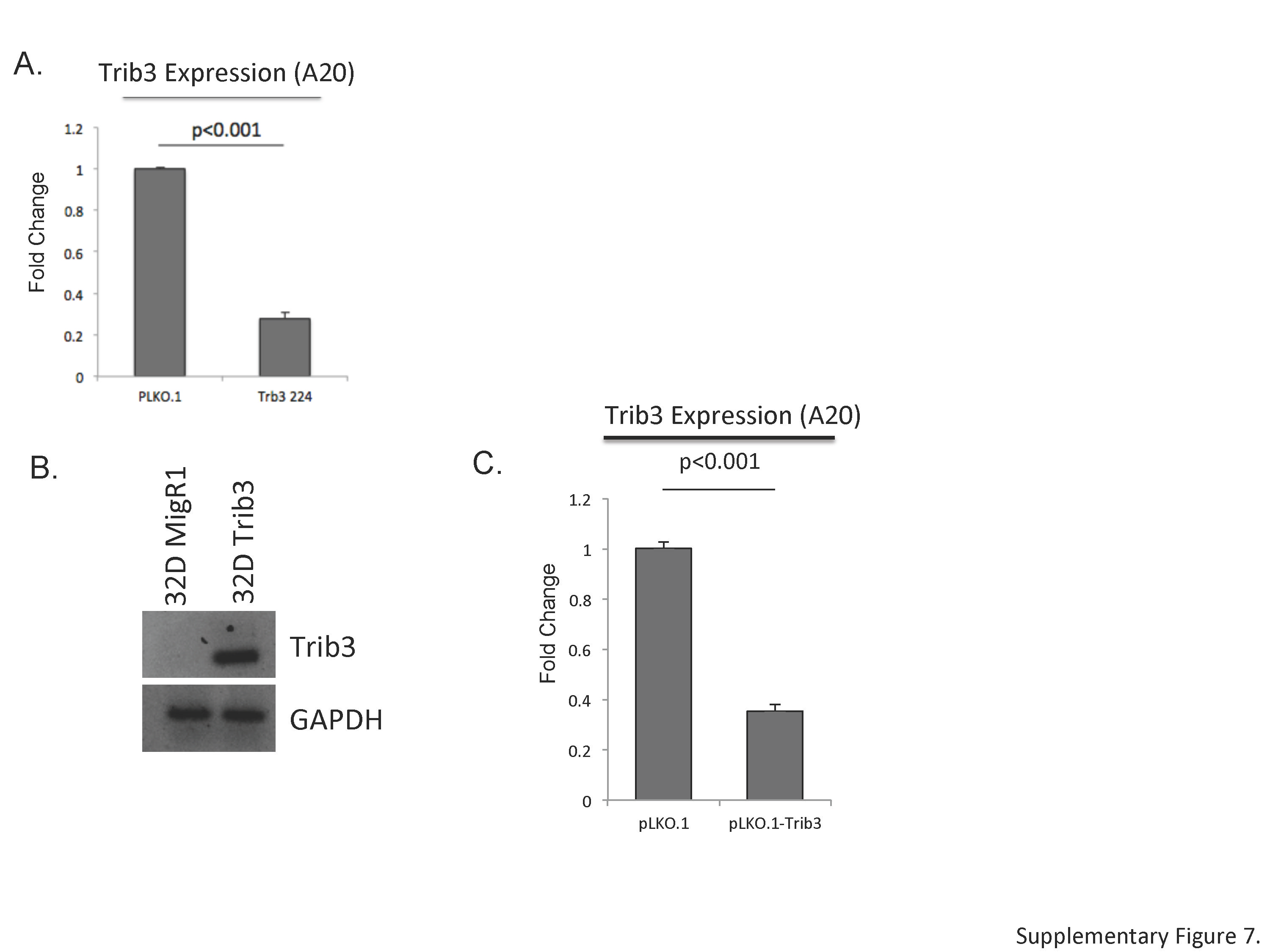

Supplement: S7 Fig — A) 70Z/3 pre B cells were transduced with control or Trib3 shRNA knockdown vectors. Trib3 knockdown was confirmed by qRT-PCR. B) 32Dcl cells were transduced with control or Trib3 overexpression vectors. Confirmation of Trib3 overexpression was done using RT-PCR. C) A20 cells were transduced with control or Trib3 knockdown vectors. Confirmation of Trib3 knockdown was done using qRT-PCR. Statistical analysis done by unpaired students t-test. (TIFF) [file pgen.1006887.s010.tiff]

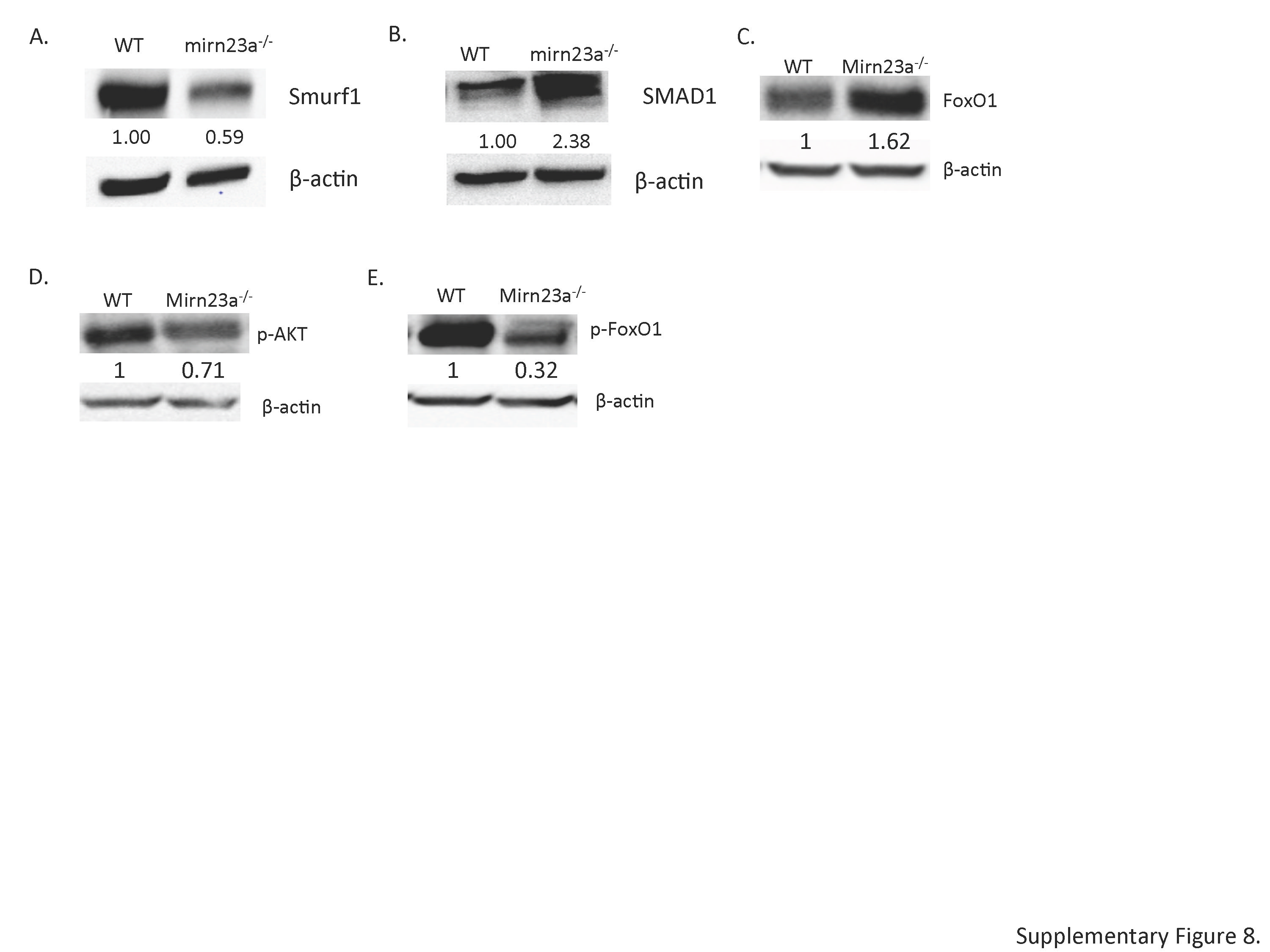

Supplement: S8 Fig — Whole cell lysates were collected from primary multipotent EML cell lines derived from wildtype and mirn23a-/- mice. Numbers designate fold change in protein levels as determined by densitometry. A) Protein expression of Smurf1 was analyzed by immunoblot in wildtype and mirn23a-/- EML cells. B) SMAD1 protein expression was analyzed by immunoblot in wildtype or mirn23a-/- EMLs. C) FoxO1 protein expression was analyzed in wildtype and mirn23a-/- EML cells. D) p-AKT protein expression was analyzed by immunoblot. E) p-FoxO1 protein expression was analyzed by immunoblot. (TIFF) [file pgen.1006887.s011.tiff]
